# Beyond Associations Between Color and Emotion: A Scoping Review of Task-Based Evidence on Color Properties, Emotional Attention, and Cognitive Control

**DOI:** 10.3390/bs16101769

**Published:** 2026-09-28

**Authors:** Chaosheng Tang, Tianning Gu, Junyi Hua, Xinying Zhan, Tianyi Chu, Shengming Yu

**Affiliations:** 1 School of Psychology, Shanghai University of Sport, 650 Qingyuanhuan Road, Yangpu District, Shanghai 200438, China; 2 School of Intelligent Sports Engineering, Shanghai University of Sport, 650 Qingyuanhuan Road, Yangpu District, Shanghai 200438, China; amaogu7@gmail.com (T.G.); 13764478638@163.com (J.H.); zxyzxk1234562025@outlook.com (X.Z.); 18930989335@163.com (T.C.)

**Keywords:** color, colour, hue, saturation, lightness, emotional attention, cognitive control, scoping review

## Abstract

Color may influence emotional cognition through perceptual salience, learned affective meaning, and response compatibility, yet task-based evidence has not been synthesized. Reported under the Preferred Reporting Items for Systematic Reviews and Meta-Analyses (PRISMA) extension for Scoping Reviews (PRISMA-ScR) and the PRISMA extension for reporting literature searches (PRISMA-S), we conducted a scoping review of experiments that varied color during emotional attention, cognitive control, or emotional information processing. Five database groups were searched on 26 July 2026 and rerun as a pre-submission update on 17 August 2026; three completed citation-searching rounds were also used. Independent screening yielded 299 included reports. Full-text charting organized these reports into 294 study families and 4923 descriptive result rows. Reported result directions varied across color properties, task roles, emotional materials, comparators, and response levels. Most families contained detected and null results, while 44 internally conflicting rows were retained for descriptive analysis. Although 569 rows from 43 families provided directly weightable effect estimates, no cluster met prespecified homogeneity criteria, so effects were not pooled. In applied settings, color sometimes changed gaze, local task performance, or affect, but transfer to beliefs, sustained attention, or general performance was inconsistent. This descriptive mapping does not establish a general performance benefit or impairment for any named color. Conflict and inhibition paradigms rarely manipulated saturation or lightness independently, leaving a specific design gap in research on color and cognitive control.

## 1. Introduction

Color can change what people notice, how quickly they respond, and which response they select when tasks involve emotional information or an explicit affective context. These effects matter outside the laboratory because color is used in websites, graphic warnings, workplaces, educational robots, reading aids, and clinical attention tasks (Byrne et al., 2019; De Ruyter & Van Dantzig, 2019; Domínguez-Borràs et al., 2013; Fang et al., 2026; A. Ludlow et al., 2008; Venni & Bétrancourt, 2020). Yet an effect attributed to the labels red or blue may reflect several variables at once. Hue, saturation, lightness, luminance, chromatic contrast, learned meaning, and response mapping often vary together (Elliot & Maier, 2014; Meier et al., 2012). Named-color comparisons are legitimate experimental categories. Attributing an effect to a specific physical color property requires reported coordinates, calibrated presentation, and control of the remaining dimensions (Jonauskaite & Mohr, 2025). This measurement problem is especially important in emotional attention and cognitive control. A faster response or a longer fixation may result from perceptual salience, affective congruence, or response compatibility, and each explanation requires a different experimental contrast (Elliot & Maier, 2014).

Most research on color and emotion has examined abstract associations, preferences, or subjective ratings. Jonauskaite and Mohr (2025) synthesized 132 English-language peer-reviewed articles published between 1895 and 2022, involving 42,266 participants from 64 countries. That review deliberately excluded cognitive task performance and associations tied to specific situations. Its scope leaves open whether color changes attentional capture, disengagement, interference, or inhibition when people process emotional material. A review of daytime light effects also found that attention and affect can overlap while remaining distinct outcomes (Bjerrum et al., 2024). Individual studies have manipulated color in emotional Stroop, flanker, go/no-go, dot-probe, and visual-search tasks for decades (Y. Cao et al., 2021; Chamaret, 2012; Ciccone, 2018; A. Jiang et al., 2026). Their findings remain difficult to combine because the tasks assign color different roles and measure different processes. A color main effect in an emotional task is insufficient evidence of emotional modulation unless the design includes an emotional contrast or tests the interaction between color and emotion.

Following JBI (formerly the Joanna Briggs Institute) guidance (Peters et al., 2024), this scoping review maps experiments that explicitly varied color and measured emotional attention, cognitive control, or the processing of emotional information. The scope includes tasks with emotional stimuli and tasks explicitly linked to affective meaning or an emotional state. For this review, emotional attention refers to attentional selection, capture, holding, or disengagement involving emotionally relevant information. Attention, control, and emotional information processing are examined together because a color cue can attract attention, interfere with a response, or change an appraisal within the same task. The review maps these outcomes separately. For each study family, the review records the tested color property, the role of color in the task, its relation to the emotional material, and the measured outcome. It assesses whether the design separates perceptual salience, learned affective meaning, arousal, and response compatibility. It also examines whether stimulus reporting permits reproduction and whether comparable studies could support quantitative synthesis. Applied findings are interpreted through a narrower transfer question: does a change in gaze, reaction time, or physiology correspond to a functional outcome in the intended setting? A laboratory effect supports practical use only when it is confirmed in the target population under the relevant device, illumination, and task conditions.

## 2. Materials and Methods

### 2.1. Review Design, Protocol, and Reporting

We conducted a scoping review following the JBI Manual for Evidence Synthesis (Peters et al., 2024). We followed the Preferred Reporting Items for Systematic Reviews and Meta-Analyses Extension for Scoping Reviews (PRISMA-ScR) for the review report (Tricco et al., 2018) and the PRISMA extension for reporting literature searches (PRISMA-S) for the search report (Rethlefsen et al., 2021).

The English protocol was frozen locally on 26 July 2026, before formal database searching, screening, and data charting. The protocol specified the review questions, eligibility criteria, information sources, screening process, charting fields, and descriptive synthesis plan. Dated amendments recorded all later changes to database syntax, accessible database coverage, eligible report types, search updating, and citation searching. Eligibility amendment EA-01 required researchers to manipulate, assign, or experimentally control an eligible visible color condition. Observational color predictors alone therefore did not meet the eligibility criteria. Database-source amendment DBA-01 documented the accessible EBSCOhost replacement layer (Section 2.4). Search-strategy amendments SSA-01 and SSA-02 documented the frozen Scopus branches and revised OpenAlex route. Search-update amendment SUA-01 documented that the source groups were not rerun during extraction and citation searching. Search-update amendment SUA-02 documented the pre-submission rerun on 17 August 2026 and its integration with the frozen baseline. Eligibility amendment EA-02 added complete English undergraduate honors theses and original empirical book chapters with complete methods and results. It also required a retrospective audit of earlier report-type exclusions (Section 2.3). Citation-chasing amendments (CCA-01 to CCA-04) documented the temporary pause and resumption, search direction, seed sets, deduplication rules, and stopping rule for citation searching (Section 2.5). Each dated change log recorded the reason for the amendment and its expected effect. No amendment changed a completed independent reviewer judgment.

The protocol was registered in Open Science Framework (OSF) Registries under identifier zbx74 on 2 August 2026. The registration is now publicly accessible. It followed formal database searching, deduplication, title-and-abstract screening, full-text screening, and the charting pilot. It preceded formal full-review charting and evidence synthesis. It is a post-screening, pre-formal-charting transparency record. It does not constitute a prospective preregistration. The registration can be accessed at https://osf.io/zbx74/, accessed on 22 September 2026.

### 2.2. Review Questions

The primary question concerned reported effects of experimentally varied color properties on emotional attention, cognitive control, and emotional information processing. Secondary questions examined how the evidence was distributed across color dimensions, color roles, tasks, emotional materials, populations, contexts, and measurement modalities. They also asked how reported patterns varied across these features. Further questions concerned proposed mechanisms, reporting completeness, and the feasibility of meta-analysis.

### 2.3. Eligibility Criteria

We included original experimental and quasi-experimental studies with human participants of any age. Clinical and non-clinical samples were eligible, as were laboratory, online, field, interface, and environmental settings. Researchers had to manipulate, assign, or experimentally control a visible color condition. Eligible properties included hue, saturation, chroma, lightness, brightness, luminance, named colors, and reported color coordinates. Color could define a target, cue, distractor, response feature, background, object, scene, interface, or illumination condition. We excluded naturally occurring color measures used only as observational predictors and post hoc color analyses without a prespecified experimental condition.

An eligible result had to estimate a planned color main effect, a specific color contrast, or a color-by-emotion effect. The result had to concern attention, cognitive control, decision, memory, emotional information processing, or a task-linked affective outcome. Eligible outcomes included response time, accuracy, errors, choice, memory, eye movements, pupillometry, autonomic measures, electroencephalography (EEG), event-related potential (ERP) measures, hemodynamic measures, and self-reported affect within an eligible emotional–cognitive design. The report also had to include emotional stimuli or meaning, valence, arousal, discrete emotion, mood, anxiety, fear, threat, stress, or another explicit affective component. Additional criteria applied when the affective component was only a manipulation check or parallel outcome in a task without emotional material. The report had to state a conceptual link between the color-related emotional state and the cognitive outcome or analyze their relationship.

We excluded pure color–emotion association judgments and color-preference studies without an eligible task. We also excluded studies in which color appeared only as a response label, material description, uncontrolled observation, or unrelated control. Passive light exposure was ineligible without an emotional–cognitive process. Reviews, protocols, editorials, commentaries, abstracts without a complete report, and non-human studies were excluded.

Eligible publication types comprised peer-reviewed journal articles, complete English doctoral and master’s theses, and full conference papers. Eligibility amendment EA-02, decided by the project lead on 5 August 2026, added complete English undergraduate honors theses and original empirical book chapters with extractable methods and results. One complete English undergraduate honors thesis (Olsen, 2010) and one original empirical book chapter (Langeslag et al., 2011) met the content criteria and were included under this amendment. A retrospective audit returned four earlier report-type exclusions, all complete original empirical book chapters, to renewed independent dual full-text screening. All four were excluded on content grounds. Only reports with an available English full text were eligible. This English-only scope was confirmed on 26 July 2026, and the review did not include Chinese-language literature. We applied no publication-year limit.

### 2.4. Information Sources and Database Searches

The formal database search was completed on 26 July 2026. We searched five source groups from inception through that date: PubMed, OpenAlex, Scopus, ProQuest Dissertations & Theses Global, and an EBSCOhost layer. The EBSCOhost layer comprised Academic Search Ultimate, MEDLINE, Education Resources Information Center (ERIC), and OpenDissertations. We identify this four-database set as an EBSCOhost replacement layer. The verified institutional entitlement did not include American Psychological Association (APA) PsycINFO, and we report this access gap as a limitation. APA PsycINFO, Web of Science, and Google Scholar were never formal sources, and they contributed no records. Pilot searches of Chinese databases informed decisions about scope but contributed no records to the formal review.

The searches returned 27,690 records. PubMed returned 2934 records. OpenAlex returned 14,270 unique works. Scopus returned 5672 records across a frozen main branch and a mutually exclusive supplementary branch. ProQuest returned 1510 records. The EBSCOhost layer returned 3304 unique accession numbers.

The strategy combined color terms with terms for emotion and cognitive tasks. Color terms covered American and British spellings, color dimensions, grayscale terms, and named colors. Emotional terms covered valence, arousal, mood, anxiety, fear, threat, stress, and discrete emotions. Task and outcome terms covered attention, cognitive control, conflict, interference, inhibition, Stroop, Flanker, dot probe, visual search, lexical decision, priming, memory, eye tracking, EEG, and ERP. In accordance with PRISMA-S (Rethlefsen et al., 2021), we retained each source’s native syntax, full query text, search date, result count, and access notes. Source exports were left unchanged after export.

Under amendment SUA-01, we did not rerun the five database source groups during the next 19 days of extraction and citation searching. A developmental PubMed and OpenAlex rerun was kept only as audit material. Its records did not enter the formal record set, screening, PRISMA counts, or synthesis. Citation searching continued through 13 August 2026 (Section 2.5). Under amendment SUA-02, we reran the five source groups on 17 August 2026 before submission. The update produced 556 source rows with stable identifiers. Cross-source deduplication removed 43 rows, leaving 513 update entities. Exact comparison with the frozen 19,088-record baseline removed 51 records, leaving 462 for screening. Reviewers excluded 455 records at title and abstract screening and classified one duplicate candidate. Six records entered full-text resolution. Five English full texts were obtained and assessed. One SSRN record was resolved through formal-version verification under exclusion code FT02. Five records were excluded, and one report was included. The included report contributed seven verified result rows. The update was merged with the frozen initial search and did not replace it.

### 2.5. Citation Searching

Citation searching followed four versioned amendments (CCA-01 to CCA-04) and comprised three completed screening rounds. CCA-01 used the reports included at that time as anchors. Reconciliation with the database set and supplemental anchor routes produced 7575 deduplicated candidates. After a temporary pause, CCA-02 resumed the search with seven additional anchors and contributed 222 candidates that did not overlap with the first set. The combined CCA-01 and CCA-02 set contained 7797 records for title and abstract screening. Reviewers excluded 7259 records and classified eight as administrative duplicate candidates. They sought 530 reports for retrieval. Of these, 60 were not retrieved, one was an administrative duplicate, and one was an invalid or damaged citation. Reviewers assessed 468 full texts, excluded 378, and included 90 unique reports.

CCA-03 used these 90 newly included reports as forward and backward citation anchors. OpenAlex returned 12,003 citation edges and 11,813 source candidate rows. Exact deduplication produced 8316 entities. Removing 1675 entities already present in the review left 6641 records for title and abstract screening. Reviewers excluded 6180 records and classified 255 as duplicates. They sought 206 reports. Among them, 52 were not retrieved, and 17 used previously acquired full texts. Reviewers assessed 137 newly acquired reports, excluded 86, and initially included 51. A later identity audit found that one included record had been paired with an ineligible full text. Removing that report left 50 unique new reports from CCA-03.

CCA-04 was the final round. It used the 51 reports initially included in CCA-03 as backward-citation anchors. We searched backward citations only and did not conduct a fourth round. Local reference extraction from legally obtained full texts was reconciled with OpenAlex referenced_works. Project-level exact deduplication against the formal database records, all included reports, and all earlier citation-searching candidates yielded 3097 entities. Of these, 1294 had enough abstract information for eligibility screening. The other 1803 lacked the title-and-abstract information required for eligibility assessment. Under the documented stopping rule, we retained them as an unassessed no-abstract administrative hold set. They were neither screened nor classified as excluded. Two human reviewers independently screened the 1294 records. Their consensus excluded 1285 records and advanced nine reports to full-text assessment. Five reports were excluded, and four were initially included. Bibliographic and full-text checks showed that one initially included report duplicated a report from an earlier round. Its removal left three unique new reports from CCA-04.

The three citation-searching rounds added 143 unique reports: 90 from CCA-01 and CCA-02, 50 from CCA-03, and three from CCA-04. Together with the 155 database-branch reports, they yielded 298 reports before the pre-submission update. The citation-searching branch contained 263 records classified as duplicates at screening. These comprised 8 from CCA-01 and CCA-02 and 255 from CCA-03. CCA-04 contributed no screening-stage duplicates. Its 1803-record no-abstract hold remained a separate administrative category outside the screening denominator. We retained round-specific counts because later rounds reused earlier records and full texts.

### 2.6. Record Management and Study Selection

Each record retained its source database and native identifier. We normalized digital object identifier (DOI) values to lowercase canonical strings and preserved PubMed identifier (PMID) values and other native identifiers. Automatic deduplication used exact DOI, exact PMID, and normalized title-plus-year rules. Reviewers assessed similar titles, and fuzzy similarity never caused automatic deletion.

Automatic rules removed 8192 database records. Reviewer consensus removed another 410 fuzzy duplicate candidates, leaving 19,088 records for title and abstract screening. C.T. was a psychology student. T.G. was a student in Data Science and Big Data Technology and provided technical support for literature searching and screening. Before formal screening, C.T. and T.G. piloted the eligibility labels and discussed their application to establish a shared interpretation of the criteria. C.T. and T.G. independently applied the same frozen eligibility criteria at both screening stages. They then compared their decisions and resolved disagreements through discussion to reach consensus. The reviewers excluded 18,035 records during title and abstract screening and sought 1053 full texts. They could not retrieve 213 reports. These were recorded as an administrative retrieval outcome and kept separate from eligibility exclusions. Reviewers assessed 840 full texts, excluded 685 with one primary reason for each, and included 155 reports from the database branch.

Citation-searching and update records underwent the same eligibility assessment, dual independent screening, and consensus resolution. Administrative non-retrieval, duplicate classifications, the no-abstract hold, and eligibility exclusions remained distinct categories. Table S7 reports primary full-text exclusion reasons by search branch. The initial database and citation-searching branches yielded 298 unique reports, and the pre-submission update added one report. The flow is final: screening and the planned update are complete.

### 2.7. Data Charting and Row Granularity

We piloted the charting form on 19 reports. The pilot produced 101 result rows and was used to calibrate field definitions, source locators, and row granularity. The final form contained 91 fields. C.T. completed primary extraction, and T.G. checked the retained content against the full text. They resolved discrepancies before each freeze.

The review distinguished nine nested units. A *record* was any bibliographic entry identified by a search method before eligibility assessment. A *report*, or *publication report*, was one publication sought, retrieved, and assessed for eligibility, such as a journal article, thesis, full conference paper, or book chapter. A *study family* grouped reports that used the same participants, data, or overlapping samples. Within a report, an *experiment* was one reported study, and a *sample* was one distinct participant group within an experiment. A *contrast* was one planned color comparison. An *outcome* was one measured variable under a defined orientation, and a *time window* was one measurement occasion or epoch. These units were not interchangeable. One report could contain several experiments and samples. One study family could contain several reports, such as a thesis and its derived journal articles. A multi-sample report could link to two study families. Study families were the primary denominator for evidence counts.

The ninth unit was the atomic charting unit, termed a *result row*. Each result row represented one unique combination of seven fields: study family, report, experiment, sample, contrast, outcome, and time window. We split a compound row when it contained multiple independently interpretable contrasts or outcomes. Color main effects and color-by-emotion interactions were charted in separate rows. A color main effect was not interpreted as evidence of emotion-specific modulation. We removed a row when it duplicated another algebraic result, lacked an eligible color effect, or could not be supported by the source. Coverage audits added missing independent results. The seven-field key had to be unique in the combined file.

The form recorded bibliographic data, design, sample, color property, color values, color space, color role, display or medium, comparator, luminance and contrast control, emotional component, task, cognitive process, measurement modality, outcome, model, statistic, effect estimate, uncertainty, result direction, proposed mechanism, author interpretation, evidence quotation, and source location. We coded missing information as not reported and did not infer it from other statistics. The only exception was a prespecified transformation later evaluated for meta-analysis feasibility (Section 2.11). Automated tools and generative artificial intelligence (AI) assisted in candidate extraction, but reviewers checked all retained content against the full text. Each retained result preserved either a literal Results passage or an identifiable table or figure extract. It also retained a page, table, figure, or section locator. Corrections were written back only after review.

### 2.8. Source Conflicts and Final Verification

Reviewers compared Results text, tables, figures, and supplements whenever values or conclusions differed within a source. They preserved both conflicting passages. They corrected a value only when the source showed a resolvable label error, transcription error, or reporting convention. Notes documented rounding, sample denominators, model definitions, and the reason for each adopted value. Reviewers did not resolve a source-internal contradiction by recalculating an author’s result or allowing a Discussion statement to overwrite Results. Human adjudication preserved the conflicting source statements and assigned the row a verified-conflict status. These rows were eligible only for descriptive mapping. Uncertain directions were coded as unclear, and unavailable numerical values as not reported. Rows without eligible evidence, duplicate rows, and rows outside the review scope received a rejected status.

The citation-searching extraction table contained 4101 candidate result rows. The final review classified 3867 as verified, 133 as verified with correction, 48 as verified source-internal conflicts, and 53 as rejected. All 100 issue records raised during verification were closed. The database-branch extraction contained 980 verified rows. Four of the 48 conflict rows belonged to reports later superseded under the canonical-report rules (Section 2.9). The final descriptive set therefore contained 44 source-internal conflict rows.

### 2.9. Report Deduplication and Study-Family Mapping

We audited report relationships across batches using DOI, normalized title, authorship, year, full-text hash, sample size, design, and stimulus details. Report-level supersession kept every included publication in the bibliographic inventory but selected one preferred source for each duplicated result set. Overlapping publications were therefore not counted as independent evidence.

We applied four supersession rules. First, we identified a later-indexed duplicate record of the *Fertile Green* report. Its 18 rows were superseded in favor of the canonical record (Lichtenfeld et al., 2012). Second, a 2020 conference chapter described the same 24-participant, four-color, within-participant functional near-infrared spectroscopy (fNIRS) sample, protocol, and results as the completed International Conference on Information Systems (ICIS) report. Its 10 rows were superseded, and both reports were assigned to the same study family (Nissen, 2020a, 2020b). Third, a doctoral thesis reused two samples already reported in journal articles. Experiment E01 mapped to the hue experiment, and experiment E02 mapped to the chroma experiment. The corresponding article rows superseded the 76 thesis rows (Duan, 2017; Duan et al., 2018a, 2018b). Fourth, an earlier book chapter shared its 296-participant sample with a more complete journal report. The journal report superseded the chapter’s eight rows and carried the study family forward (J.-E. Pelet & Papadopoulou, 2010; J.-É. Pelet & Papadopoulou, 2012).

The initial extraction sources and the pre-submission update contained 5088 raw result rows before supersession. The four rules removed 112 rows, comprising 108 non-conflict rows and four source-conflict rows. This left 4976 canonical all-status rows. Removing the 53 rejected rows left 4923 rows for descriptive synthesis. This set comprised 4879 non-conflict candidates assessed for quantitative feasibility (Section 2.11) and 44 human-adjudicated source-internal conflict rows restricted to descriptive use. The bibliographic inventory retained all 299 included reports. Appendix A cites each report, and Table S1 links it to its study family. The canonical result set contained 294 result-bearing preferred reports representing 294 study families.

### 2.10. Reporting Completeness

We did not conduct a formal risk-of-bias assessment or assess certainty of evidence. We calculated no aggregate quality score and excluded no study solely because of methodological quality. We mapped interpretability and reproducibility fields separately. These covered randomization, counterbalancing, blinding, sample-size rationale, missing data, color values, color space, display or medium, luminance control, contrast control, funding, and conflicts of interest. We calculated completeness at the study-family level. A field counted as reported if at least one report in the family provided usable information. Reporting completeness does not establish methodological quality or certainty of evidence.

We summarized the recorded content of funding and conflict-of-interest fields by study family. Funding categories distinguished support sources, personal financial support, explicit statements of no external or specific funding, unspecified funding mentions, and unavailable information. The conflict-of-interest indicator included explicit absence declarations and disclosed financial relationships, whether or not they appeared under a separate conflict-of-interest heading. We displayed financial-relationship disclosures without a separate declaration as their own category. Commercial affiliation alone did not count as a funding or conflict-of-interest declaration. Targeted full-text checks resolved 19 ambiguous field entries in 18 families. Explicit statements of no funding or no competing interests counted as reported information. Table S2 preserves the submitted text, corrected text, and source locations.

### 2.11. Synthesis and Meta-Analysis Feasibility

As specified in the frozen protocol, the primary synthesis combined descriptive evidence mapping with structured narrative synthesis. We mapped reports to study families before counting them. Each family was assigned to one primary theme based on its task, outcome, color role, and study aim. The six themes were attention and cognitive control, emotion perception and decision-making, memory and learning, environmental lighting and context, emotion and physiological response, and other cognitive and behavioral tasks. Non-exclusive descriptors could overlap across families. These descriptors included color dimension, color role, measurement modality, and candidate mechanism. For the color-role-by-modality map, we paired roles and modalities within the same result row, then deduplicated them by study family. We interpreted result direction relative to the extracted contrast and outcome orientation. The labels were positive, negative, no detectable difference, mixed, and unclear. Positive and negative did not have a uniform numerical or beneficial meaning across outcomes. A no-detectable-difference code does not establish an effect of zero. Supplementary Methods S8 documents the coding rules, source-specific statistical criteria, and family summaries with examples from Table S3. Direction counts were descriptive and did not constitute vote-counting evidence for an average causal effect. Source-conflict rows remained in the descriptive evidence map but were excluded from all quantitative pooling candidates.

We summarized the population and context fields already retained in Table S3 by study family. Country, setting, and reported mean-age descriptors could overlap. We preserved descriptions of clinical status without inferring diagnoses or health from student or community recruitment. Numerical sample-size summaries were restricted to families with one charted experiment-sample key and one unambiguous analyzed participant count. Other sample-size descriptions remained as text. Table S2 retains the original descriptions, derived categories, and source locations.

We evaluated meta-analysis feasibility after extraction without fitting a pooled model. A potentially poolable cluster required at least three non-overlapping study families, defined as having no known participant or data overlap. It also required a common color contrast, a common outcome and estimand, compatible designs, computable sampling variances, and a manageable dependence structure. A directly usable row had either a reported effect with a standard error or confidence interval or a correlation with a sample size that allowed for derivation of Fisher-z variance. We classified test statistics that required design assumptions as potentially convertible, and they did not enter the direct-effect gate. Among 4879 non-conflict candidate rows, 569 rows from 43 study families contained directly weightable effects. Another 1190 rows were potentially convertible to a common metric. Only one coarse cluster reached three families with directly weighted effects. It combined correlations and mean differences, unrelated environmental and affective outcomes, 230 distinct contrasts, and many dependent effects from one family. It failed the manual homogeneity audit. No cluster met the prespecified requirements. We therefore conducted no pooled meta-analysis and retained the approved descriptive mapping and structured narrative synthesis.

### 2.12. Use of Automation and Generative AI

Scripts supported record management, deduplication, validation, vocabulary normalization, table generation, and numerical checks. Between January 2026 and August 2026, the authors used OpenAI ChatGPT (GPT-5.6, sol variant) and OpenAI Codex CLI. These tools supported search-term development, record-management and analysis code, preparation of candidate charting, consistency checks, visualization, error detection, and manuscript language drafting. Throughout the review, automation and generative AI only sorted, flagged, organized, and cross-checked records and drafts. These tools did not make final eligibility decisions, act as independent screeners, substitute for human judgment, or verify extracted results. C.T. and T.G. made all screening decisions and checked the source evidence for every retained charting item. The authors reviewed the final analysis and manuscript and remain responsible for their accuracy.

## 3. Results

### 3.1. Evidence Base and Unit of Analysis

The review included 299 unique publication reports. These reports represented 294 study families and yielded 4923 descriptive result rows. Figure 1 shows the identification and screening process. Searches across the five database groups returned 27,690 records. Automatic deduplication removed 8192 records, and reviewer consensus removed another 410 fuzzy-duplicate candidates. This left 19,088 records for title and abstract screening. Reviewers excluded 18,035 records and sought 1053 full texts. Of these, 213 could not be retrieved. Reviewers assessed 840 full texts and excluded 685 with a primary reason. The database branch contributed 155 included reports. Three rounds of citation searching added 143 unique reports: 90 from rounds CCA-01/02, 50 from round CCA-03, and 3 from the final backward-searching round (CCA-04). The 17 August update yielded 556 source rows with stable identifiers, 513 cross-source entities, and 462 records after exact baseline deduplication. Reviewers excluded 455 records at title and abstract screening, classified one duplicate candidate, resolved six reports at full-text screening, excluded five, and included one. The final evidence base therefore contained 299 reports. No screening or update step remains pending.

The review uses three analytic units. A *report* is the publication unit retrieved and screened. A *study family* combines reports that share participants, samples, or data and prevents duplicate counting of the same evidence. Some reports belonged to the same family, while one report with multiple samples contributed to two families. This mapping explains why 299 reports formed 294 families. A *result row* is one unique combination of study family, report, experiment, sample, contrast, outcome, and time window. Result rows are charting units, not independent studies. Their number describes extraction volume rather than evidence strength. Unless noted otherwise, prevalence estimates and thematic counts use the 294 study families as the denominator. Result-row counts are reported separately.

The initial extraction batches and the pre-submission update contained 5088 raw result rows. The database branch contributed 980 verified rows. Citation searching contributed 4101 candidate rows, including 3867 verified rows, 133 verified with correction, 48 verified as internally conflicting, and 53 rejected. The update contributed seven verified rows. Four canonical-report rules then superseded 112 duplicate or shared-sample rows. A duplicate record of the *Fertile Green* report contributed 18 rows, which were superseded by the canonical record (Lichtenfeld et al., 2012). A 2020 conference chapter reported the same 24-participant, four-color, within-participant fNIRS sample, protocol, and results as the completed ICIS conference report. Its 10 rows were superseded, and both reports were assigned to one family (Nissen, 2020a, 2020b). A doctoral thesis reused two samples previously reported in journal articles. Experiment E01 mapped to the hue experiment, and E02 mapped to the chroma experiment. The 76 thesis rows were therefore superseded by the article rows (Duan, 2017; Duan et al., 2018a, 2018b). An earlier book chapter shared a 296-participant sample with a more complete journal report. Its eight rows were superseded, and the journal report carried the family forward (J.-E. Pelet & Papadopoulou, 2010; J.-É. Pelet & Papadopoulou, 2012). These decisions left 4976 rows in the canonical all-status ledger. Removing the 53 rejected rows produced the 4923 rows used in the descriptive synthesis. The frozen citation-search batch contained 48 internally conflicting rows. Four were removed through the supersession rules, leaving 44 source-conflict rows in the canonical descriptive ledger. These rows remained in the descriptive map but were ineligible for quantitative analysis. The resulting candidate pool contained 4879 non-conflict rows. Section 3.11 shows that only 569 rows from 43 of the 294 families carried directly weightable effects. This limited coverage led to a descriptive synthesis.

Each family belonged to one of six mutually exclusive themes (Table 1). Emotion perception and decision-making was the largest, with 97 of 294 families (33.0%) and 1620 result rows. Environmental lighting and context included 64 families (21.8%) and 1760 rows. Attention and cognitive control included 46 families (15.6%) and 479 rows. Emotion and physiological response included 36 families (12.2%) and 372 rows. Memory and learning included 34 families (11.6%) and 406 rows. Other cognitive and behavioral tasks included 17 families (5.8%) and 286 rows. The family counts total 294, and the row counts total 4923. Row volume indicates charting density and does not measure evidence strength.

### 3.2. Population and Study Contexts

Table 2 summarizes the population, setting, and design information retained in Table S3. Reported sample mean ages were available for 194 families and ranged from 5.5 to 77.5 years. The records explicitly described healthy or nonclinical participants in 186 families. Another 16 families recorded a clinical or health condition, with or without comparison participants. The most frequently recorded countries were the United States (67 families) and China (57). Laboratory or controlled-room descriptions appeared in 218 families, online settings in 31, and schools or classrooms in 30. For the 149 families with one charted sample and an unambiguous analyzed N, the median was 42 participants, with an interquartile range (IQR) of 24 to 100. The remaining sample-size descriptions were retained in Table S2 without summing repeated outcomes or potentially overlapping samples.

### 3.3. Color Properties, Functional Roles, and Measurement Coverage

The 294 families were published from 1921 to 2026, with most appearing recently. Sixteen families (5.4%) appeared before 2000, and 24 (8.2%) appeared between 2000 and 2009. Another 125 (42.5%) appeared between 2010 and 2019, and 129 (43.9%) appeared between 2020 and 2026 (Figure 2). For display, each family was assigned to one of four mutually exclusive search-route categories: 152 database-only, 140 citation-searching-only, one represented in both initial routes, and one added only by the pre-submission update. The database and citation branches covered 153 and 141 families, respectively, with one family shared by both. Journal articles accounted for 239 of 294 families (81.3%). The remaining sources were 28 full conference papers, 12 doctoral theses, 11 master’s theses, 3 book chapters, and 1 undergraduate honors thesis. The coverage estimates below use family-level multi-label counts with 294 families as the denominator. A family could have several values within one category, so category counts may exceed 294.

Most studies examined hue or named colors. Figure 3 maps color categories by primary theme and shows mutually exclusive family-level result patterns. Hue appeared in 208 of 294 families (70.7%), and named colors appeared in 168 (57.1%). Physical specification was much less common. Color coordinates appeared in 56 families (19.0%), saturation in 46 (15.6%), and lightness in 41 (13.9%). Chroma appeared in 31 (10.5%), brightness in 26 (8.8%), and luminance in 23 (7.8%). Two families (0.7%) were coded for color temperature, both within the environmental theme. Coverage of non-hue dimensions also differed by theme. Saturation appeared in 17 of 97 emotion-perception families and 9 of 34 memory families. It appeared in only 6 of 46 attention families and 6 of 64 environmental families. Color coordinates were most common in the environmental and emotion-perception themes, with 22 families in each. The evidence therefore describes which color was shown more often than how saturated, light, or bright it was. This imbalance constrains the theme-level syntheses from Section 3.4, Section 3.5, Section 3.6, Section 3.7, Section 3.8 and Section 3.9.

Color also served different functions within the experiments. Background color appeared in 86 of 294 families (29.3%), object color in 58 (19.7%), and illumination in 57 (19.4%). Interface color appeared in 43 (14.6%), scene color in 41 (13.9%), target color in 38 (12.9%), and cue color in 34 (11.6%). Response features appeared in 10 families (3.4%), and distractors appeared in 9 (3.1%). These roles depended on the theme. Illumination dominated the environmental theme (36 of 64 families) and was rare elsewhere. Targets and cues clustered in attention and cognitive control, with 13 of 46 and 10 of 46 families, respectively. Backgrounds and interfaces were most common in emotion perception and decision-making, with 32 of 97 and 25 of 97 families. A nominal color could therefore be the object being judged, an irrelevant surround, or a feature that carried the response. Functional role was a major source of variation in the mapped results.

Behavioral and self-report outcomes dominated the evidence base. Figure 4 links each measurement modality to the color role recorded in the same extracted result row. Behavioral measures appeared in 242 of 294 families (82.3%), and self-report appeared in 174 (59.2%). Physiological and neurophysiological measures were less common. Autonomic measures and EEG each appeared in 30 families (10.2%), ERP in 19 (6.5%), eye tracking in 19 (6.5%), and pupillometry in 9 (3.1%). Their distribution also varied by theme. EEG occurred most often in emotion and physiological response (11 of 36 families), as did ERP (8 of 36). Eye tracking was most common in attention and cognitive control (6 of 46). Autonomic measures clustered in the environmental theme (16 of 64). The synthesis treats these modalities as distinct response levels. A neural, autonomic, or gaze effect does not establish behavioral benefit, and a null behavioral result does not rule out a physiological response.

Family-level direction patterns were calculated after excluding the 44 source-conflict rows. Most families—210 of 294 (71.4%)—contained both detected and null results. Here, “detected” denotes the stored positive, negative, or mixed codes described in Supplementary Methods S8. Another 37 families (12.6%) contained mixed or opposing detected directions. Thirty-two (10.9%) contained only positively coded results, and 13 (4.4%) were null only. One family was negative only, and one was unclear or descriptive only. Positive and negative preserve the recorded orientation of each outcome and do not indicate benefit or harm across unlike measures. Section 3.4, Section 3.5, Section 3.6, Section 3.7, Section 3.8 and Section 3.9 describe these patterns within each theme.

### 3.4. Attention and Cognitive Control

Forty-six study families examined attention or cognitive control. They contributed 479 of the 4923 verified descriptive result rows and represented 15.6% of all families. This theme is the focus of the review because it addresses a specific gap. Emotion perception and decision-making is larger, with 97 families and 1620 rows. The systematic review by Jonauskaite and Mohr (2025) synthesized context-free associations between color and emotion. It excluded color effects on cognition and task performance. Individual studies have long used color in emotional Stroop, go/no-go, flanker, dot-probe, and visual-search tasks, but those studies estimate different processes. Explicit conflict and inhibition designs seldom manipulated saturation or lightness independently of named color. This leaves a design-intersection gap. The present synthesis records each family’s color contrast, the role of color in the task, its relation to the emotional material, and the measured response level.

The cognitive-control submapping used only frozen extraction fields. It classified 18 families as core cognitive control and 28 as general attention. This classification concerns the task’s control demands. The general-attention subset also includes tasks with emotional content. Forty-two families already had native core or general labels. The remaining four had no native subset label and were classified with a prespecified fallback rule. The rule required either a canonical conflict or inhibition task family or a frozen process label for interference, inhibition, or response conflict. None of the four met these conditions, so all entered general attention. Outcome favorability played no part in the classification. Some core families still placed the control-relevant contrast within a noncanonical paradigm. Examples include a dot-probe study carrying an inhibition process label (S. Müller et al., 2016), a depletion-priming study labeled for interference and inhibition despite having no conflict manipulation (Bertrams et al., 2015), and a cancellation task that combined colored backgrounds with affective music (Simoës-Perlant et al., 2026). Separating these subsets avoids treating orienting, capture, vigilance, conflict, and inhibition as one effect. The synthesis also distinguishes abstract color–emotion associations, experienced emotion, attentional orienting and disengagement, perceptual salience, conflict and inhibition, response selection, and neural or physiological response.

The task labels confirm this heterogeneity. Families could have more than one label, so the counts are not additive. Visual search appeared in 14 families, dot-probe in 7, emotional Stroop in 6, and memory-related attention tasks in 4. Decision or classification, go/no-go, lexical decision, and priming each appeared in three families. One family used a flanker task. Seventeen had an “other” task label because the taxonomy did not cover every paradigm. Hue appeared in 32 families and named colors in 27. Saturation appeared in six, while brightness, color coordinates, lightness, and luminance each appeared in four. Color served as a target in 13 families, a cue in 10, a background in 8, an object feature in 7, a response feature in 6, a distractor in 5, and illumination in 4. Behavioral outcomes appeared in 42 families and self-report in 17. Eye tracking appeared in six, ERP in four, EEG in two, and autonomic measures and pupillometry in one each. At the row level, 163 results were positive, 73 were negative, 176 showed no detectable difference, 65 were mixed, and 2 were unclear. These direction codes preserve the original outcome orientation and are not pooled estimates. At the family level, 28 families combined detected and null results. Seven had mixed detected directions, seven were positive only under the recorded orientations, and four were null only. One family contained verified source-conflict rows (Ioan et al., 2007). Only those rows were restricted to description.

#### 3.4.1. Core Cognitive Control

Eighteen families measured conflict, interference, inhibition, or response selection. Color rarely had the same function across studies. Ink or font color carried the response in color-naming and lexical variants (Meier et al., 2004; Xu & Zhang, 2019; T. Zhu et al., 2025). Facial color was the emotional object (Park & Choi, 2025). Priming color appeared as a background and target (Kang et al., 2022), and interface color appeared as a background (F. Wang et al., 2025). Synesthetic photism colors were naming targets (Perry & Henik, 2013). Named colors were naming targets under manipulated software luminosity (Ioan et al., 2007). Other roles included scene color in a flanker setting (Michaelis, 2013), a singleton distractor (Moran & Moser, 2015), and gain- or loss-associated cues in a dot-probe task carrying an inhibition label (S. Müller et al., 2016). Brightness served as the response dimension in metaphorical congruency judgments (Huang et al., 2018, 2022; Meier et al., 2004). The remaining designs used color primes after ego depletion (Bertrams et al., 2015), background color with music in a cancellation task (Simoës-Perlant et al., 2026), or colored illumination in three go/no-go families (Ciccone, 2018; Iwakiri et al., 1999; Y. Zhu et al., 2017). A paradigm label alone therefore does not identify what the color manipulation estimates.

Six families used emotional Stroop designs. Reported patterns differed across the tested color–emotion relations. Table S3 provides the contrast-specific statistics summarized below. In one family, blue and red primes carried associations with sadness and anger. Prime color interacted with target emotion for reaction time (F(4, 156) = 16.832, *p* < 0.001). The report also found main effects of prime color on reaction time and accuracy. In the incongruent condition, responses to sad targets were faster after blue than red primes. Red primes facilitated responses to angry faces. A PR-650 spectrophotometer matched luminance to $100\;{\mathrm{cd/m}}^{2}$, which isolates the hue contrast from luminance. All seven verified rows were positively coded (Kang et al., 2022).

Another family colored the emotional face itself. Color, emotion, and priming had no main effects. Color interacted with emotion for reaction time (F(1, 37) = 5.464, *p* = 0.025) and accuracy (F(1, 37) = 12.153, *p* = 0.001). Red favored anger accuracy (t(38) = −2.570, *p* = 0.014), and yellow favored happiness accuracy (t(38) = 3.286, *p* = 0.002). The study did not measure luminance. All five rows were positively coded (Park & Choi, 2025).

A third family combined a valence-word Stroop task and a color-word Stroop task with ERP and eye tracking. Incongruent coloring, defined as positive words in green and negative words in red, slowed responses relative to congruent coloring (F(1, 38) = 8.121, *p* < 0.01). It also changed N170, N2c, and late positive complex (LPC) amplitudes. Product evaluation changed only under high cognitive load (F(1, 46) = 8.52, *p* < 0.01). Behavioral directions were mixed across nine rows (Xu & Zhang, 2019). In grapheme-color synesthesia, the emotional valence of the conflict altered interference. The group-by-congruity interaction on color-naming time was significant (F(3, 30) = 4.9, *p* < 0.007). Synesthetes also showed a valence-by-congruity interaction (F(2, 4) = 8.8, *p* < 0.0001). Incongruent negative pairings produced the fastest naming, and seven of eight rows were coded as mixed (Perry & Henik, 2013).

One color-naming family recorded only hemodynamic outcomes for positive and negative colors shown with emotional words. Three of four verified fNIRS rows showed no detectable difference. One showed a color effect on oxygenated hemoglobin at 850 nm (coefficient = −0.020, *p* = 0.012). No behavioral row was recorded. This was therefore a case of neural modulation without a recorded behavioral counterpart. The frozen vocabulary placed fNIRS outside the neural category and coded these outcomes as behavior and other (T. Zhu et al., 2025). The sixth family varied the interface background across five named colors and reported Lab color-space lightness (L) values. The omnibus reaction-time effect was mixed. Yellow backgrounds were associated with slower responses and lower accuracy. NASA Task Load Index (NASA-TLX) frustration and mental demand also differed by color. The five rows did not report exact test statistics (F. Wang et al., 2025). Across the six emotional Stroop families, reported patterns varied across color-emotion relations and outcomes. Their direction followed the color-emotion relation made relevant by the task.

Another family measured Stroop interference without emotional stimuli. The study also collected Symptom Checklist-90-Revised (SCL-90-R) symptom scores. It examined the association between depression scores and red-specific Stroop interference. Participants named red, green, and blue shown at full or halved software luminosity. Color interacted with gender for Stroop interference (F = 7.4, *p* < 0.001). Red produced about 50 ms more interference than pooled green and blue in men (t = 2.8, *p* < 0.01) but about 55 ms less in women (t = 2.6, *p* < 0.01). Dim colors slowed naming of incongruent color words by about 40 ms (t = −3.2, *p* < 0.001) (Ioan et al., 2007). The authors called the full colors equiluminant based on hue–saturation–lightness (HSL) luminosity but made no photometric measurement. This was the only attention family with verified source-conflict rows. Only those rows entered the descriptive synthesis without quantitative eligibility.

One family embedded a flanker and Attention Network Test design in color-manipulated environmental photographs. Neither scene color nor environment type affected executive-control reaction time, state anxiety, or picture preference (F(3, 107) = 0.62, *p* = 0.60). The scenes depicted urban, foliage, or aquatic environments. Five of six rows showed no detectable difference. A time-by-color interaction on backwards digit span did not reach the threshold (F(3, 107) = 2.60, *p* = 0.083) (Michaelis, 2013). This was the clearest null pattern in the core set. It shows that control effects observed with laboratory stimuli did not automatically extend to scene color.

Three go/no-go families used illumination rather than an object or target feature. Their results concerned arousal and alertness more than uniform inhibition benefits. One design crossed illuminance at 200 or 1200 lx with correlated color temperature. Illuminance affected change in alertness (F(1, 56) = 11.72, *p* = 0.001, d = 0.91). It interacted with color temperature for go/no-go reaction time (F(1, 56) = 7.15, *p* = 0.010, d = 0.70). The interaction also affected two-back reaction time (F(1, 56) = 7.41, *p* = 0.009, d = 0.74) and facial-emotion judgment time (F(1, 56) = 5.21, *p* = 0.026, d = 0.63) (Y. Zhu et al., 2017).

Another family compared red, blue, green, and yellow light with white light. Color affected go/no-go reaction time (F(4, 136) = 17.35, *p* < 0.001) and self-reported impulsiveness (F(4, 96) = 7.14, *p* < 0.001). EEG band power also differed for alpha (F(4, 16) = 24.33, *p* < 0.001), beta (F(4, 16) = 14.28, *p* < 0.001), and theta (F(4, 16) = 5.63, *p* = 0.005). The risk-taking effect was smaller (F(4, 136) = 2.8, *p* = 0.029). Luminosity varied with color, which the authors identified as a limitation. The nine rows included positive, negative, no-difference, and unclear codes (Ciccone, 2018).

The third family recorded a brain electrical response, i.e., contingent negative variation (CNV), during go/no-go performance under light sources that differed in color temperature and rendering index. For lamps with a color rendering index of 88, the early CNV amplitude at the frontal midline electrode (Fz) was larger under 6500 K light than under 3000 to 3700 K light (*p* < 0.01). Relative alpha energy was lower under 6500 K light in the same comparison (*p* < 0.01). These neural differences were not accompanied by a statistically detected difference in mean reaction time (Iwakiri et al., 1999). In all three families, illuminance or spectral composition was the manipulated factor. Their rows estimate lighting effects on inhibitory task performance rather than effects of a named color alone.

Three families examined metaphorical brightness–valence correspondence rather than stimulus–response incompatibility. One family manipulated congruency between bright and positive or dark and negative stimuli. It used valence judgment, brightness judgment, priming, and a go/no-go variant intended to increase valence activation. The congruent versus incongruent reaction-time contrast reached *p* < 0.05 for valence judgment, priming, and go/no-go but not brightness judgment. Three of four rows were positively coded (Huang et al., 2018). A larger family crossed white or black font with word valence on a constant 50% gray background. Font color interacted with word valence for response latency (F = 7.78, *p* = 0.013) and accuracy (F = 12.78, *p* = 0.002) in the evaluative experiments. Only 6 of 46 verified rows reached *p* < 0.05, while 25 showed no detectable difference. This family was therefore mostly null, with interactions confined to particular tasks (Meier et al., 2004).

The third family placed brightness-valence congruency within a task-set manipulation. Brightness affected reaction time (β = −1.854, t = −2.473, *p* = 0.013). This effect was qualified by congruency proportion by brightness (β = 1.889, t = 2.520, *p* = 0.012) and valence by brightness (β = 1.757, t = 2.343, *p* = 0.019). A three-way interaction among congruency proportion, valence, and brightness was also present (β = 2.409, t = 3.213, *p* = 0.001). The congruency effect varied with the proportion of congruent trials and stimulus-onset asynchrony (β = 1.609, t = 2.147, *p* = 0.032) (Huang et al., 2022). None of the three families reported photometric calibration. Their brightness manipulations therefore represent coarse white versus black contrasts. These results show that learned color-affect mappings can support conflict paradigms without an irrelevant color dimension competing for the response. Strategic and temporal conditions changed the size of the effect.

The remaining core families placed control-relevant contrasts in noncanonical paradigms. A visual-search family measured distractor interference as the reaction-time cost of a present versus absent color singleton. It used the N2-posterior-contralateral (N2pc) component to measure active suppression. Responses were slower when a distractor was present (F(2, 242) = 65.94, *p* < 0.001). Trait anxiety interacted with distractor condition for reaction time (F(2, 236) = 20.12, *p* < 0.001) and N2pc amplitude (F(2, 236) = 12.33, *p* < 0.001). Trait anxiety correlated with the ipsilateral reaction-time cost (r = 0.49, *p* < 0.001) and inversely with the N2pc cost (r = −0.33, *p* < 0.0012). All seven behavioral and ERP rows were positively coded (Moran & Moser, 2015).

The core dot-probe family was classified by its frozen inhibition label. It paired colors with monetary gain and loss and separated engagement from disengagement. The validity effect was large (F(1, 52) = 16.19, *p* < 0.001, $\eta_{p}^{2}$ = 0.237). Disengagement from valent cues exceeded the baseline (F(1, 52) = 6.65, *p* = 0.013, $\eta_{p}^{2}$ = 0.113). Post hoc validity effects were present for gain ($d_{z}$ = 0.46, *p* < 0.002) and loss cues ($d_{z}$ = 0.33, *p* = 0.021) (S. Müller et al., 2016). Its congruency labels refer to cue validity, engagement, and disengagement rather than response conflict. The results therefore concern learned-value attention.

In the depletion-priming family, ego-depleted participants solved fewer arithmetic problems after red than gray primes. Adjusted means were 2.81 and 5.71 (F(1, 30) = 5.83, *p* = 0.02). They also solved fewer intelligence-test items (F(1, 35) = 4.35, *p* = 0.04) and reported more distraction by worries (F(1, 60) = 5.84, *p* = 0.02). The design contained no conflict manipulation, so these rows estimate priming effects on task performance rather than inhibition (Bertrams et al., 2015). The cancellation-task family paired a yellow or gray background with joyful or sad music. Participants crossed out more targets on yellow (49.23 versus 47.51, F(1, 248) = 19.852, *p* = 0.001). The music-color congruent condition exceeded the incongruent condition (55.16 versus 43.20, F(3, 248) = 8.09, *p* = 0.001). Self-reported valence also changed (F(1, 248) = 280.552, *p* = 0.001). Stimulus preparation standardized luminance and contrast (Simoës-Perlant et al., 2026).

Four core families combined behavioral outcomes with EEG or ERP (Ciccone, 2018; Iwakiri et al., 1999; Moran & Moser, 2015; Xu & Zhang, 2019). One further family recorded fNIRS under the frozen “other” code (T. Zhu et al., 2025). Color altered interference costs, congruency interactions, or neural indices. The direction depended on the relevant color relation, and no consistent improvement in performance was evident. In two families, neural modulation had no behavioral counterpart (Iwakiri et al., 1999; T. Zhu et al., 2025). Candidate mechanism labels for the core families included affective congruency (Huang et al., 2018; Kang et al., 2022; Park & Choi, 2025; Perry & Henik, 2013; Xu & Zhang, 2019), conflict modulation (Ciccone, 2018; Huang et al., 2022; Ioan et al., 2007; Meier et al., 2004; Moran & Moser, 2015; Perry & Henik, 2013; Xu & Zhang, 2019; T. Zhu et al., 2025), arousal (Ciccone, 2018; Iwakiri et al., 1999; Michaelis, 2013; F. Wang et al., 2025), and perceptual salience (Iwakiri et al., 1999; Moran & Moser, 2015; F. Wang et al., 2025). These labels do not establish causal chains. A label was directly supported when its frozen evidence basis referred to a targeted test or manipulation. Otherwise, it retained the primary authors’ interpretation.

#### 3.4.2. Orienting, Search, and Sustained Attention

The other 28 families measured orienting, capture, holding and disengagement, vigilance, or perceptual decision without response conflict or inhibition. They are reported separately because capture and search speed differ from conflict resolution.

Twelve of the 14 visual-search families belonged to this subset. Salient or emotionally diagnostic target colors often accelerated search. In participants with neglect, a red-tinted pop-out target was found faster than a neutral identity target (1370 versus 2022 ms, t(26) = 12.11, *p* < 0.0001). It was also found faster than fearful or happy expression targets (t(26) = 5.78 and 3.46, both *p* < 0.005). The faces came from a luminance-normalized standardized set (N. Lucas & Vuilleumier, 2008). A red target link on a small display was found faster than a black link (F(1, 90) = 20.318, *p* < 0.001). Red also received higher dominance and likability ratings (F(1, 90) = 4.542, *p* = 0.036, and F(1, 90) = 4.939, *p* = 0.029). All three rows were positively coded (Y. Cao et al., 2021).

Red-tinted cues guided search more efficiently than neutral identity cues (1526 versus 2344 ms, t = −7.46, *p* < 0.0001). They also outperformed fearful and happy expression cues (both *p* < 0.0001). Eight of nine rows were negatively coded under the recorded reaction-time orientation (Domínguez-Borràs et al., 2020). Colored targets were found faster than emotional targets (946 versus 1110 ms, F(1, 20) = 28.38, *p* < 0.001). The condition omnibus was significant (F(4, 80) = 130.15, *p* < 0.001), as was the emotional/non-emotional-by-condition interaction (F(1, 20) = 13.04, *p* = 0.002) (Santos et al., 2010).

Task-irrelevant colors preferred for particular valences increased target processing during association (F(1, 34) = 31.47, *p* < 0.001). Individual color-valence preference predicted valence-dependent target facilitation (r = 0.63, *p* < 0.001). A color-by-valence interaction remained during testing (F(1, 179.05) = 8.98, *p* = 0.003). A chroma meter set the colors (Stanković et al., 2024). Colors associated with monetary reward or loss biased additional-singleton capture. Singleton type had a main effect (F(4, 83) = 55.17, *p* < 0.001), qualified by a singleton-by-group interaction (F(4, 83) = 2.71, *p* = 0.035). The colors were matched for luminance (P. Müller & Wentura, 2023). Attentional-bias training toward or away from a red singleton reduced its reaction-time cost after feature-mode rather than singleton-mode training (5.22 versus 20.40 ms, F(1, 55) = 7.28, *p* = 0.009). Training also interacted with trait anxiety in change in state anxiety (F(1, 55) = 5.24, *p* = 0.03) (Moser et al., 2015).

Three families separated color attributes instead of comparing named colors. A virtual-reality stowage task varied label saturation at 0, 15, 45, and 90 percent. Colored rather than white labels affected negative affect (F(1, 49) = 4.92, *p* = 0.031, $\eta_{p}^{2}$ = 0.091). Saturation level affected positive and negative affect (F(3, 147) = 30.24 and 13.26, both *p* < 0.001). Colored labels also improved search accuracy (F(1, 49) = 8.50, *p* < 0.01) (A. Jiang et al., 2026). An eye-tracking and pupillometry family compared children with autism spectrum disorder and typically developing children across hue, luminance, and saturation series at $80\;{\mathrm{cd/m}}^{2}$. The autism group had less total fixation time (11,970 versus 16,897 ms, F = 11.13, q < 0.001, false discovery rate (FDR) correction) and fewer fixations (F = 5.88, q = 0.027). Phasic pupil coupling to luminance transitions was also attenuated. Static pupil diameter did not differ (*p* = 0.93). Fourteen of 20 rows were negatively coded (W. Jiang & Liu, 2025). A scene-viewing family varied hue and saturation. Its color contrasts did not reliably attract fixations beyond their grayscale correlates. One row was mixed, four showed no difference, and none reached significance (Chamaret, 2012).

Applied viewing studies also produced outcome-specific effects. Harmonious rather than disharmonious website color increased composition ratings (t(32) = 2.093, *p* = 0.044) and pragmatic-quality ratings (t(32) = 2.44, *p* = 0.020). Disharmonious color drew more fixations (t(28) = −2.77, *p* = 0.009) and improved recall (t(32) = −2.04, *p* = 0.0497). Stimulus preparation controlled brightness (Venni & Bétrancourt, 2020). Full-color graphic warning labels increased gaze duration relative to black-and-white labels (B = −6.78, standard error (SE) = 2.26, *p* = 0.01). They did not change beliefs or intentions (Byrne et al., 2019).

Six families used dot-probe or related orienting tasks without conflict demands. Lightness-matched red and green cues had International Commission on Illumination (CIE) lightness–chroma–hue (LCh) coordinates, with L ≈ 61. Red slowed target responses (F(1, 30) = 5.34, *p* = 0.03) and interacted with cue-target spatial congruency (F(1, 30) = 5.02, *p* = 0.03). In this design, congruency meant spatial validity rather than response conflict. Emotion interacted with color for LPC amplitude (F(1, 40) = 9.35, *p* < 0.001), with simple effects for red and anger and for green and happiness (T. Xia et al., 2018). Emotionally diagnostic hues reduced attentional capture and holding relative to non-diagnostic hues. Hue diagnosticity interacted with validity (F(1, 49) = 5.29, *p* = 0.03) and had a main effect (F(1, 49) = 7.13, *p* = 0.01). The study measured image chromaticity and luminance. All nine rows were positively coded (McMenamin et al., 2013).

A large family crossed object color with facial expression. Color, expression, and target face interacted for attentional adhesion (F(2, 156) = 3.07, *p* = 0.048, $\eta_{p}^{2}$ = 0.02). Within red, angry and happy faces held attention longer than neutral faces (t(78) = 2.64, *p* = 0.01, and t(78) = 2.19, *p* = 0.03). There was no color main effect, and 19 of 26 rows showed no detectable difference (Buechner et al., 2014). Red rather than non-red cues produced lateralized ERP responses in an emotional context. Their behavioral counterparts were mixed and lacked reported statistics, so the neural modulation did not indicate a uniform behavioral advantage (Kuniecki et al., 2015). In participants with migraine, chromatic rather than achromatic aversive patterns affected dot-probe reaction time (*p* = 0.006, $\eta_{p}^{2}$ = 0.27). Color also interacted with visual-discomfort group (*p* = 0.002, $\eta_{p}^{2}$ = 0.34) (Hine & White, 2021). Another family presented highly saturated red, lightly saturated red, or gray before emotional image pairs. Reaction times did not differ by saturation, and saturation did not interact with phobia strength. Both verified rows showed no difference (Thoksakis, 2022).

Four families measured sustained or selective attention in applied settings. A dynamic-lighting intervention specified illuminance at 150 and 1500 lx. Compared with standard office lighting, it did not change concentration or N-back performance. Seven of nine rows showed no detectable difference. The intervention did change negative valence in facial expression (t = −2.54, *p* = 0.015) and eye-contact avoidance (t = 5.8, *p* = 0.016) (De Ruyter & Van Dantzig, 2019). In educational robotics, social-robot headband color affected the total attention duration of autistic children (F = 6.539, *p* = 0.001). Post hoc contrasts favored warm over cool headbands, but exact statistics were not reported (Fang et al., 2026). Colored reading overlays interacted with diagnosis. Children with autism spectrum disorder read more words per minute with their selected overlay than without it, whereas controls showed the opposite pattern. The overlay-by-group interaction was F(1, 34) = 29.7, *p* < 0.001. Completion time showed a parallel interaction (F(1, 50) = 12.170, *p* = 0.001) (A. Ludlow et al., 2008).

The largest orienting family conditioned a red triangle as an aversive signal in a patient with visual extinction. Contralesional miss rates for conditioned red fell from 68% before conditioning to 41% afterward ($\chi^{2}$ = 4.04, *p* = 0.045). The reduction was stronger on bilateral trials (Yates $\chi^{2}$ = 19.1, *p* < 0.0001). Skin-conductance responses were larger for conditioned red than for unconditioned blue and green stimuli (F(1, 54) = 17.4, *p* < 0.001). Seen conditioned red stimuli also produced a right anterior fusiform blood-oxygen-level-dependent (BOLD) response (z = 3.19, *p* < 0.001). All triangles were equiluminant, but hue and conditioning status were bundled in one stimulus. The contrast therefore estimates affective learning about a color rather than a pure hue effect (Domínguez-Borràs et al., 2013).

Other attention families further limited the pattern. Flanker errors increased subsequent rapid serial visual presentation (RSVP) target misses from 10.27% to 21.78% (F = 22.083, *p* < 0.001). Across the color-related contrasts in this family, seven rows were negatively coded, four were mixed, and 14 showed no difference (Houtman & Notebaert, 2013). Poster viewing under different color temperatures produced three no-difference eye-tracking rows (Yu, 2024). An uncategorized attention task that varied hue, saturation, and brightness produced 13 no-difference rows (L. Zhao et al., 2024).

Three families used lexical decision or priming. In lexical decision, colored valence words produced a color-by-lexicality interaction (F(2, 244) = 10.297, *p* < 0.001) and a color-by-valence interaction (F(4, 488) = 2.513, *p* = 0.04). Responses were faster to green than red words (*p* = 0.01). Color also affected accuracy (F(2, 244) = 6.557, *p* = 0.002). Row directions were divided between mixed and positive (Bortolotti et al., 2024). Color primes for emotion words affected lexical judgment time (F(1, 35) = 4.94, *p* < 0.03). The effect was qualified by relatedness by color (F(1, 35) = 5.96, *p* < 0.02) and word by color (F(1, 35) = 7.84, *p* < 0.01). The authors characterized support for their hypotheses as trends (Killelea, 1987). Subliminal facial expressions on red or white backgrounds produced no detectable effect on impressions. Heterochromatic flicker photometry equated the backgrounds for luminance. Both verified rows in this direct test of the red-intensification hypothesis were null (Schakner, 1981).

Within general attention, color modulated attention when it was salient, emotionally diagnostic, or associated with learned value (Y. Cao et al., 2021; N. Lucas & Vuilleumier, 2008; McMenamin et al., 2013; P. Müller & Wentura, 2023; Santos et al., 2010; T. Xia et al., 2018). It did not alter sustained attention, orienting after pretrial saturation, poster viewing, or several priming and viewing outcomes (De Ruyter & Van Dantzig, 2019; Schakner, 1981; Thoksakis, 2022; Yu, 2024; L. Zhao et al., 2024). Four families were uniformly positively coded (Y. Cao et al., 2021; Fang et al., 2026; Hine & White, 2021; McMenamin et al., 2013). All four uniformly no-difference families in the theme also belonged to this subset (Schakner, 1981; Thoksakis, 2022; Yu, 2024; L. Zhao et al., 2024). Some null families documented photometric control or illuminance (De Ruyter & Van Dantzig, 2019; Schakner, 1981). Their null results cannot be attributed simply to failed stimulus control.

#### 3.4.3. Cross-Cutting Synthesis

Only 18 of the 46 families measured conflict, inhibition, interference, or response selection (Bertrams et al., 2015; Ciccone, 2018; Huang et al., 2018, 2022; Ioan et al., 2007; Iwakiri et al., 1999; Kang et al., 2022; Meier et al., 2004; Michaelis, 2013; Moran & Moser, 2015; S. Müller et al., 2016; Park & Choi, 2025; Perry & Henik, 2013; Simoës-Perlant et al., 2026; F. Wang et al., 2025; Xu & Zhang, 2019; T. Zhu et al., 2025; Y. Zhu et al., 2017). Even within these families, control demands ranged from canonical stimulus–response incompatibility to learned-value cueing and metaphorical congruency. The other 28 families measured general attention. A summary that treats all 46 as evidence about cognitive control would overstate the number that engaged control processes.

Color occupied every functional role in the attention theme. It was a target in 13 families, a cue in 10, a background in 8, an object feature in 7, a response feature in 6, a distractor in 5, and illumination in 4. These roles were associated with different effects. Target colors mainly produced capture and facilitation (Y. Cao et al., 2021; A. Jiang et al., 2026; N. Lucas & Vuilleumier, 2008; Santos et al., 2010; T. Xia et al., 2018). Distractor colors produced interference costs related to anxiety and control demand (Moran & Moser, 2015; Moser et al., 2015; P. Müller & Wentura, 2023). Cue colors produced validity, engagement, and disengagement effects (Domínguez-Borràs et al., 2020; McMenamin et al., 2013; S. Müller et al., 2016). Background and interface colors affected arousal, workload, and affective tone (Simoës-Perlant et al., 2026; F. Wang et al., 2025). Response-feature colors produced congruency and metaphorical-conflict effects (Bortolotti et al., 2024; Huang et al., 2018; Meier et al., 2004; Xu & Zhang, 2019; T. Zhu et al., 2025). Illumination affected alertness and broad performance measures rather than color-specific inhibition (Ciccone, 2018; Iwakiri et al., 1999; Y. Zhu et al., 2017).

Attribute coverage was uneven. Saturation appeared in six families (Byrne et al., 2019; Chamaret, 2012; A. Jiang et al., 2026; W. Jiang & Liu, 2025; Thoksakis, 2022; L. Zhao et al., 2024). Lightness, brightness, and luminance each appeared in four. The frozen fields identify which dimensions were involved, but independent manipulation can be determined only from each contrast. Factorial attribute variation occurred in the virtual-reality saturation series (A. Jiang et al., 2026); the pretrial saturation levels in one dot-probe family (Thoksakis, 2022); and the hue, luminance, and saturation series in the eye-tracking family (W. Jiang & Liu, 2025). All three belonged to general attention. Within core cognitive control, only three metaphorical-congruency families manipulated brightness independently, and none used photometric calibration (Huang et al., 2018, 2022; Meier et al., 2004). No core family independently manipulated saturation. One core family matched luminance photometrically at $100\;{\mathrm{cd/m}}^{2}$ with a spectrophotometer (Kang et al., 2022). Figure 5 maps these task and attribute intersections. Seven families compared named colors without coordinates (Bortolotti et al., 2024; Fang et al., 2026; Hine & White, 2021; Kuniecki et al., 2015; Stanković et al., 2024; Yu, 2024; T. Zhu et al., 2025). Their effects cannot be assigned to a particular photometric property. The unfilled intersection is therefore an explicit conflict design that independently varies saturation or lightness. Existing studies address nearby questions but estimate different quantities.

Fifteen families documented luminance or lightness control, matching, or measurement. The methods included a chroma meter (Stanković et al., 2024), software-based brightness control (Venni & Bétrancourt, 2020), specified illuminance at 150 and 1500 lx (De Ruyter & Van Dantzig, 2019), and fixed luminance at $80\;{\mathrm{cd/m}}^{2}$ (W. Jiang & Liu, 2025). Other families used a luminance-normalized face set (N. Lucas & Vuilleumier, 2008), standardized luminance and contrast (Simoës-Perlant et al., 2026), spectrophotometric matching to $100\;{\mathrm{cd/m}}^{2}$ (Kang et al., 2022), or reported LAB L values (F. Wang et al., 2025). Further methods included heterochromatic flicker photometry (Schakner, 1981), measured luminance and chromaticity (McMenamin et al., 2013), CIE LCh lightness matching (T. Xia et al., 2018), luminance-matched colors (P. Müller & Wentura, 2023), approximately equal luminance and size (Domínguez-Borràs et al., 2020), equiluminant stimuli (Domínguez-Borràs et al., 2013), and lightness- and chroma-matched red and blue (Buechner et al., 2014).

Six more families manipulated illuminance, luminosity, or brightness and reported the levels without equiluminance or photometric calibration (Huang et al., 2022; Ioan et al., 2007; Iwakiri et al., 1999; A. Jiang et al., 2026; Meier et al., 2004; Y. Zhu et al., 2017). Two explicitly lacked control. In one, luminance varied by color and was noted as a limitation (Ciccone, 2018). Another used unmeasured device defaults (Park & Choi, 2025). Two provided only partial control by matching picture size and resolution (Michaelis, 2013) or stating that colorimetric calibration was absent (Santos et al., 2010). The other families did not report luminance control. Their apparent hue effects remain confounded with luminance and salience.

Six families combined behavioral outcomes with EEG or ERP (Ciccone, 2018; Iwakiri et al., 1999; Kuniecki et al., 2015; Moran & Moser, 2015; T. Xia et al., 2018; Xu & Zhang, 2019). One recorded fNIRS hemoglobin responses under the frozen “other” modality (T. Zhu et al., 2025). Another added autonomic and hemodynamic measures to behavior (Domínguez-Borràs et al., 2013). Pupillometry in the eye-tracking family (W. Jiang & Liu, 2025) was oculomotor rather than neural. Behavioral and neural results did not correspond in a simple way. Three families showed neural or physiological modulation without a behavioral counterpart. Early CNV amplitude and alpha power differed by light source, while the sole behavioral row showed no difference (Iwakiri et al., 1999). The fNIRS family detected one color-sensitive channel but recorded no behavior (T. Zhu et al., 2025). Red cues produced lateralized ERP responses in emotional contexts, while two behavioral rows were mixed and lacked statistics (Kuniecki et al., 2015). The remaining dual-outcome families showed convergence across behavior and neural measures, but the directions varied (Ciccone, 2018; Moran & Moser, 2015; T. Xia et al., 2018; Xu & Zhang, 2019). Physiological modulation cannot be called improved attention or control without a supporting behavioral contrast.

The evidence basis also differed across candidate mechanism labels. Perceptual salience was mapped in 19 families, attentional capture in 18, affective congruency in 17, arousal in 13, conflict modulation in 9, embodied or metaphorical mapping in 2, and memory prioritization in 1. Six families had no recorded mechanism (Chamaret, 2012; A. Jiang et al., 2026; N. Lucas & Vuilleumier, 2008; Yu, 2024; L. Zhao et al., 2024; Y. Zhu et al., 2017). Some labels came from targeted tests or manipulations in the source. Examples include gain and loss competition in a dot-probe task (S. Müller et al., 2016), distractor costs and anxiety regressions (Moran & Moser, 2015), an operational measure of attentional adhesion (Buechner et al., 2014), hue-diagnosticity regression (McMenamin et al., 2013), and tests stratified by congruency proportion (Huang et al., 2022). Other labels came from author interpretation. These included a color-in-context account of red cues (Kuniecki et al., 2015), an affective-congruency account of facial-color interactions (Park & Choi, 2025), and a warm-color attractiveness account of robot headbands (Fang et al., 2026).

Affective congruency, arousal, perceptual salience, attentional capture, and conflict modulation recurred across independent families. They can organize the evidence but remain candidate mechanisms. Attention-arousal coupling through pupil-gaze phase locking appeared in one family (W. Jiang & Liu, 2025), so it is consistent with that account but was not replicated. Most designs did not separate competing explanations. A salience test requires controlled luminance and contrast with discriminability measures. A capture test requires cue-validity or distractor-cost contrasts. Affective congruency requires an interaction between color and emotion or a matched versus mismatched contrast. Conflict modulation requires an explicit interference, inhibition, or response-selection contrast.

The attention and cognitive-control evidence does not support a general main effect of color on cognition. Effects depended on the varied attribute, the function of color, its relation to emotional material, and the measured response level. The adjacent themes provide context for these conditional results.

Contributing reports: (Bertrams et al., 2015; Bortolotti et al., 2024; Buechner et al., 2014; Byrne et al., 2019; Y. Cao et al., 2021; Chamaret, 2012; Ciccone, 2018; De Ruyter & Van Dantzig, 2019; Domínguez-Borràs et al., 2013, 2020; Fang et al., 2026; Hine & White, 2021; Houtman & Notebaert, 2013; Huang et al., 2018, 2022; Ioan et al., 2007; Iwakiri et al., 1999; A. Jiang et al., 2026; W. Jiang & Liu, 2025; Kang et al., 2022; Killelea, 1987; Kuniecki et al., 2015; N. Lucas & Vuilleumier, 2008; A. Ludlow et al., 2008; McMenamin et al., 2013; Meier et al., 2004; Michaelis, 2013; Moran & Moser, 2015; Moser et al., 2015; P. Müller & Wentura, 2023; S. Müller et al., 2016; Park & Choi, 2025; Perry & Henik, 2013; Santos et al., 2010; Schakner, 1981; Simoës-Perlant et al., 2026; Stanković et al., 2024; Thoksakis, 2022; Venni & Bétrancourt, 2020; F. Wang et al., 2025; T. Xia et al., 2018; Xu & Zhang, 2019; Yu, 2024; L. Zhao et al., 2024; T. Zhu et al., 2025; Y. Zhu et al., 2017).

### 3.5. Emotion Perception and Decision-Making

Emotion perception and decision-making was the largest theme. It contained 97 study families and 1620 descriptive result rows, or about one-third of all families. Decision and classification tasks appeared in 55 families, recognition tasks in 17, and visual search in 1. Thirty-eight families had judgment or decision tasks coded as “other.” These included facial-expression classification, affective evaluation, consumer and interpersonal appraisal, risk and impulsivity decisions, and choices made through colored interfaces or backgrounds. Hue was examined in 73 families and named colors in 63. Color coordinates appeared in 22 families, lightness in 18, saturation in 17, chroma in 14, brightness in 9, and luminance in 1. Color served most often as a background (32 families), interface feature (25), object property (23), cue (16), or target (15). Fewer families used illumination, scene color, or a response feature. Behavioral outcomes appeared in 76 families and self-report in 48. ERP appeared in five families, EEG in four, and autonomic measures and eye tracking in three each. Pupillometry appeared in two families.

At the row level, 536 results were positive, 167 were negative, and 141 were mixed, while 670 showed no detectable difference and 106 were unclear. Null results were the largest category. At the family level, 72 families combined detected and null findings. Ten had mixed detected directions, five were null only, eight were positive only, one was negative only, and one was unclear. Nine families contained narrative conflicts within the source. Their rows remained in the description but were ineligible for quantitative analysis. Candidate mechanisms included affective congruency in 31 families, arousal in 18, perceptual salience in 16, embodied or metaphorical mapping in 11, attentional capture in 9, and conflict modulation in 7. Thirty-one families had no recorded mechanism. The synthesis separates facial color and expression perception (Section 3.5.1), abstract color–emotion associations and symbolic stimuli (Section 3.5.2), and color in judgment and decision contexts (Section 3.5.3). Section 3.5.4 summarizes the boundary conditions and reporting gaps.

#### 3.5.1. Facial Color and Emotional-Expression Perception

One group of families altered the color of the face itself. Typical manipulations changed chroma or lightness toward redness, pallor, or bluish coloration. Outcomes included expression category, intensity, and recognition. This cluster was the most internally consistent part of the theme. Kato et al. (2022) contributed 27 facial-chroma and lightness rows. All were positive under the recorded orientations, with perceptual salience mapped as the candidate mechanism. Across several independent families, facial redness, pallor, and bluish coloration changed judgments of anger, fear, happiness, and expression intensity (Kato et al., 2022; Minami et al., 2018; Nakajima et al., 2017). Recognition studies using object-level or target-level facial color showed similar patterns (Sakuta & Gyoba, 2006; SEJIMA et al., 2016; Shibusawa et al., 2025). At least three independent families therefore detected sensitivity to facial color. The designs did not distinguish low-level discriminability from affective diagnosticity.

Peripheral and neural findings were more conditional. Some families found pupillary and behavioral differences (Nakakoga, 2021; Zak, 2015; Zak et al., 2015). An EEG and eye-tracking study of colored illumination during face processing yielded more negative than positive rows (Laureanti et al., 2021). ERP research with emotional pictures found differences in evaluative processing without a uniform behavioral main effect (Cano et al., 2009). Null and near-null cases also occurred. A scene-based recognition family reported one null hue row (Fotios et al., 2017). Several small families produced mixed or scattered directions (B. Liu et al., 2026; Mammarella et al., 2016; Nie et al., 2026; Peromaa & Olkkonen, 2019; Yamada et al., 2025).

Another group held the face constant and colored its surround. The largest family crossed four background colors with face gender and expression. For female poser faces, red backgrounds increased the probability of a negative judgment. The green versus red odds ratio was 0.68 (95% confidence interval (CI) [0.56, 0.82]), the achromatic versus red ratio was 0.70 [0.58, 0.85], and the mixed versus red ratio was 0.71 [0.59, 0.86]. The omnibus *p* value was 0.0002. The same family contained 11 null and 2 unclear rows (Gil & Le Bigot, 2015). A companion family from the same group reported only positive rows for background hue in expression recognition (Gil & Le Bigot, 2014). Several smaller families were also positive-dominant (J. Choi et al., 2020; Hui et al., 2024; Liao et al., 2022).

Effects of background color were weaker or more conditional elsewhere. Takei and Imaizumi (2022) detected differences in 4 of 13 rows, and Yasuda (2007) detected a difference in 1 of 5 rows. When color conflicted with expression, incongruence was associated with slower categorization and more errors (Frühholz et al., 2011). This result fits response-selection and interference accounts but does not distinguish them. A target-color family with affective-congruency and conflict-modulation labels likewise contained positive, mixed, and null rows (Kawai et al., 2020). Families that combined colored illumination or backgrounds with self-report, EEG, or autonomic measures also produced mixed directions (X. Li et al., 2026; Y. Li, Fang, Qiu, Yu, et al., 2024; S. Song & Yamada, 2019; Viegas et al., 2024). One ERP family linked target brightness to expression classification but returned more null than positive rows (J. Chen & Song, 2025). Facial color, itself, had comparatively consistent perceptual associations. Colored surrounds changed expression judgments only for particular comparators, face categories, and outcomes.

#### 3.5.2. Symbolic Color Cues in Judgment and Priming Tasks

A separate set of families treated color as an abstract or symbolic carrier of emotion. These studies examined brightness-valence metaphors, color-word priming, and hue cues in classification tasks rather than coloring the emotional stimulus itself. The brightness-valence mapping received the most direct tests. Lakens et al. (2013) contributed 30 rows on the brightness of objects, scenes, and cues. Nineteen were positive and consistent with brightness-valence compatibility. Smaller families showed weaker or directionally unstable patterns for lightness, brightness, and luminance in target, cue, distractor, and response roles (Ishikawa et al., 2018; Jacobson et al., 2024; Meier et al., 2015; Okubo & Ishikawa, 2011). Six of seven rows were mixed in one response-feature family, indicating dependence on the stimulus–response assignment (Lakens, 2010).

Color priming was similarly conditional. A hue-priming family that combined self-report with ERP produced 10 positive and 13 null rows (Lemos et al., 2019). A named-color object-priming family produced 10 mixed and 7 positive rows among 31, showing instability within the study (Zuo & Kawabata, 2024). Word and association judgments added small, mostly positive families (Goodhew & Kidd, 2020), along with scattered or negative results (Blazhenkova et al., 2022; Kanojiya & Zinzuvadiya, 2025; Nguyen et al., 2023).

In classification tasks, color was sometimes embedded in an interface or cue. The usual pattern was a strong emotional-stimulus effect with a smaller, interaction-specific, or absent color effect. Positive-leaning families included interface-cue designs in which hue or saturation affected classification or usability judgments (G. Li & Kawabata, 2021; Shavit et al., 2013). One family placed color in the background, target, and response simultaneously (Ruba et al., 2021). Several cue- or background-based classification families contained a minority of positive rows (Forrester et al., 2025; C. Liu et al., 2018; Whitaker et al., 2015; Young et al., 2013). Other families were negative or null. One target-color family had two negative rows and one null row (Shi et al., 2015). A named-color object family contained only negative rows (C. Li et al., 2024). Interface families were mainly null or unclear (Bengart & Vogt, 2023; Silic & Cyr, 2016; Sivananthan et al., 2021). Families that crossed hue with chroma, lightness, or background role often had mixed directions (Ding et al., 2022, 2023; Hagemann et al., 2008; Sivananthan et al., 2021). These results are consistent with abstract color–emotion congruency in response selection and evaluative classification. Most patterns came from single families, however, and the comparators differed. They included red versus green, color versus achromatic, and saturated versus desaturated conditions. Few designs separated associative congruency from perceptual salience.

#### 3.5.3. Color in Judgment and Decision Contexts

The largest and most varied cluster used color in applied judgments. It covered achievement, risk and impulsivity, consumer appraisal, and interface-mediated choice. In the historically central family, brief red exposure before achievement tasks was associated with poorer performance and avoidance markers (Elliot et al., 2007). A representative row showed worse anagram performance for red than green (planned comparison, $\eta_{p}^{2}$ = 0.06, *p* < 0.05, negative direction), yet 28 of the family’s 50 rows were null and 7 were mixed. The evidence from this family supports a conditional outcome-specific effect rather than a general red impairment. Related families narrowed the claim further. A null-only family of 27 rows, which also had a conflict within the source, found no hue effect in a comparable cue and object design (Smajic et al., 2014). A red-background family distributed its 18 rows across positive, negative, and null outcomes (Maier et al., 2008). Other achievement or aptitude families were mostly null or contained a minority of negative rows (Bedwell et al., 2011; A. K. Ludlow et al., 2013).

Risk and impulsivity decisions formed another group. One hue-background family produced 17 positive rows, 5 negative rows, and 13 unclear rows across impulsivity, arousal, response time, and errors (Duan et al., 2018a). This pattern was compatible with arousal but did not isolate it. In the companion chroma report, four rows contained narrative conflicts within the source, and 27 of 43 rows were unclear. Human review retained the conflict rows for description and excluded them from quantitative analysis (Duan et al., 2018b). Another background-color decision family was mainly negative and null (Chan & Park, 2015).

Consumer and product appraisal included large positive families and clear counterexamples. Bagchi and Cheema (2012) contributed 47 rows on background and interface hue, saturation, and lightness. Nineteen were positive and 10 were negative under an arousal mapping. A red package produced more experienced guilt than a green package, with adjusted means of 2.59 and 1.74 (*p* = 0.005) (Lunardo et al., 2021). That family contained 18 positive and 30 null rows and also had a conflict within the source. The most unbalanced interface and web-color family produced 26 positive rows among 37 (Silic et al., 2017). Other interface-cue and website-appraisal families also leaned positive (Ash et al., 2013; Westerman et al., 2011; Kaspar et al., 2015; G. Li & Kawabata, 2021; Miran et al., 2017; J.-E. Pelet et al., 2013).

Adjacent applied families were mixed or null. Advertising and choice studies of background hue produced as many mixed as positive rows (Pontes & Hoegg, 2019; Pontes & Williams, 2020). Screen- and object-color appraisal families were mainly null (T. Jiang et al., 2022; Krenn, 2014; Nissen & Riedl, 2021). In the football study, uniform hue was manipulated in videos of tackles. Participants judged tackle roughness. The study tested a prediction based on the proposed association between red and aggression. It was included under the criterion for explicit affective meaning within a task-specific judgment (Krenn, 2014). Judgment studies based on illumination or ambiance were wholly or almost wholly null. One 28-row family detected no difference (Mao et al., 2018). Other families that combined eye tracking, autonomic, or behavioral outcomes also had a majority of null rows (Peromaa et al., 2025; Vyas et al., 2025).

The pre-submission update added one digital-news interface family. Red, green, and blue did not differ on the composite trust-related evaluation (F(2, 40) = 2.438, *p* = 0.100). Color effects appeared for two task-completion outcomes and two eye-tracking outcomes, but no adjusted pairwise comparison for total dwell time remained significant. Follow-up interviews linked blue and green with calmness and stability and red with tension or vigilance. The family therefore added task-dependent effects without establishing a stable color ranking (Hao & Wang, 2026).

Smaller applied families produced positive, negative, mixed, and null rows for feedback cues, product objects, scene color, and interface framing (Braun et al., 1995; Chittaro, 2016; Conway et al., 2010; Gnambs, Appel, & Oeberst, 2015; Hsieh et al., 2024; Martin-Kratzer, 2005; Rhodes, 2019; Richards & Fink, 2017; Soldat et al., 1997; Thorstenson, 2014; Tuffentsamer, 2018; Vandermause et al., 2022; L. Wang et al., 2019; Wen et al., 2025). Two families illustrate the range. Identification accuracy for an assigned feedback color was high in one ($\phi^{2}$ = 0.70, *p* < 0.001), but 47 of its 74 rows were null (Richards & Fink, 2017). In another, click behavior tracked cover color at the low end of the tested range (endpoint slope of b = −0.324, *p* < 0.001). That family also had negative, mixed, and null rows and a conflict within the source (Wen et al., 2025). Thus, even the largest families combined condition-specific effects with a majority of null outcomes.

#### 3.5.4. Boundary Conditions, Direction Heterogeneity, and Reporting Gaps

Color effects differed by property, function, emotional relation, comparator, and response level. The processing level is one boundary. An abstract color–emotion association differs from experienced emotion. A shift in expression classification differs from a change in consumer choice. A color main effect also differs from an interaction between color and emotion. Some families combined these levels within one experiment. The comparator provides another boundary. Red versus green contrasts (Elliot et al., 2007; Gil & Le Bigot, 2015; Lunardo et al., 2021) estimate a different effect than color versus achromatic or saturated versus desaturated contrasts. In Gil and Le Bigot (2015), the omnibus result depended on which alternative replaced red. Response levels also diverged within families. Self-report, behavior, ERP, autonomic measures, and pupillometry sometimes moved in different directions (Laureanti et al., 2021; Lemos et al., 2019; Y. Li, Fang, Qiu, Yu, et al., 2024; Vyas et al., 2025). A claim based on one modality therefore does not summarize the entire family.

The mechanism labels reflect these boundaries. Affective congruency appeared in 31 families, arousal in 18, perceptual salience in 16, embodied or metaphorical mapping in 11, attentional capture in 9, and conflict modulation in 7. The last two labels occurred where designs included capture- or conflict-relevant contrasts. Only the facial-color and brightness-valence clusters recurred across at least two independent families as organizing patterns. Even there, the designs did not isolate the proposed pathway.

Reporting was incomplete. Named colors appeared in 63 families, while color coordinates appeared in 22 and luminance in only 1. Most claims therefore concern named hue categories with uncontrolled or partly controlled physical properties. Saturation and lightness appeared in 17 and 18 families, respectively, and were rarely varied independently of hue. Chroma appeared in 14 families. One of these illustrates the interpretive problem: most rows were unclear, and several conflicted (Duan et al., 2018b). Null results were the largest row-level category, with 670 of 1620 rows. Another 106 rows were unclear, and nine families had narrative conflicts within the source. Thirty-one families had no recorded candidate mechanism. Applied decision studies also combined color with product category, selling mechanism, message framing, or interface context. They support conditional judgments within the tested setting but do not establish a general emotional meaning for a hue.

*Contributing report (not otherwise cited in the text above):* (Gil & Le Bigot, 2017).

### 3.6. Memory and Learning

The memory and learning theme contained 34 study families and 406 result rows, representing 11.6% of all families. The studies covered more than five decades. They ranged from an early manipulation of colored scene materials (Farley & Grant, 1973) to recent work on colored interfaces, lighting, and multimedia design (L. Chen et al., 2026; Pak et al., 2026; Qu & Kato, 2026). One family contained internally conflicting descriptive rows (Cömert & Samur, 2025). Those rows were used only in the descriptive synthesis. The theme includes color at encoding, ambient exposure during study or testing, and broad emotional-design interventions. The sections below distinguish color properties and roles, direction patterns, memory stage, and limits on interpretation.

#### 3.6.1. Color Properties, Roles, Tasks, and Outcome Modalities

Hue appeared in 23 families and named color categories in 14. Saturation appeared in 9, brightness in 4, lightness in 3, and color coordinates in 2. Chroma and luminance each appeared in one family. Color was distributed across the learning environment rather than confined to the memoranda. It served as a background in 10 families, an object property in 9, an interface feature in 7, and illumination in 4. Smaller numbers used scene color, target color, or color as a retrieval cue. Target- and object-color designs made color a feature of the material to be remembered (Blase et al., 2024; Kawasaki & Yamaguchi, 2012; Kuhbandner & Pekrun, 2013). Background, interface, and illumination designs made color an ambient context during encoding or testing (Gnambs et al., 2010; Knez, 1995; Sinclair et al., 1998). These roles estimate different effects and cannot be treated as one manipulation.

Twenty-eight families used memory tasks. Eleven used other applied or learning-related tasks, four included decision or classification, and two used explicit learning tasks. Recognition and priming each appeared in one family. The studies covered encoding, working-memory maintenance, recognition, free and delayed recall, declarative knowledge tests, and multimedia learning. Behavioral outcomes appeared in 32 families, and self-report appeared in 22. Physiological measures were uncommon. EEG appeared in four families (Chai et al., 2019; Hosseini & Ghabanchi, 2022; Kawasaki & Yamaguchi, 2012; Qu & Kato, 2026), and autonomic recording appeared in two (Elliot et al., 2011; Le et al., 2018). Most families therefore measured memory accuracy or latency, together with subjective state, allowing performance and experience to diverge within a study.

#### 3.6.2. Supportive, Null, and Mixed Results

Of the 406 rows, 151 were positive under the recorded orientations, 37 were negative, 154 showed no detectable difference, 48 were mixed, and 16 were unclear. At the family level, 24 combined detected and null results. Five had mixed detected directions, two were null only (Blase et al., 2024; Olsen, 2010), and three were positive only (Cipollone, 2024; Jung et al., 2011; Kawasaki & Yamaguchi, 2012). Positive and null rows were nearly equal in number. Color effects on memory were therefore detected often but did not extend across all outcomes within a study.

Several families reported better later performance when color distinguished encoding, matched emotional meaning, or marked relevant information. Target color affected later memory for emotional material in a pattern consistent with affective congruency (Kuhbandner & Pekrun, 2013). Color-related memory differences were accompanied by EEG correlates in one family (Chai et al., 2019). Another found positive target-hue effects in both behavior and EEG (Kawasaki & Yamaguchi, 2012). Background and scene color changed memory alongside perceptual and affective measures (Y. Wang et al., 2021). Object- and background-color families reported only positive rows (Cipollone, 2024; Jung et al., 2011). In applied multimedia work, interface and object color affected affective and learning outcomes (Um et al., 2012). Positive affect improved from before to after a lesson across design groups ($\eta_{p}^{2}$ = 0.14, *p* = 0.03) (Le et al., 2018). Another family found mostly positive effects of background and scene hue on memory, with some opposing rows (Ab Jalil et al., 2021). Named object colors also produced positive affective-congruency patterns (R. Lucas, 2022).

The largest family shows why these effects do not generalize uniformly. It compared warm, cold, and achromatic grayscale tones in multimedia learning materials across 55 rows (Liew et al., 2022). Twenty-one rows were positive, and 28 showed no detectable difference. Extraneous cognitive load had a mixed effect ($\eta_{p}^{2}$ = 0.04, *p* = 0.02). Another multimedia family found that shape and color affected some affective and learning outcomes, while others were null or moved in opposite directions (Plass et al., 2014). Positive affect differed by design and mood group ($\eta_{p}^{2}$ = 0.07, *p* = 0.036), but the direction of the specific contrasts was unclear. Emotional design therefore changed selected outcomes in selected conditions, not all outcomes relative to achromatic design.

Null results clustered in particular roles. Named background color had no detectable effect across four recognition rows (Olsen, 2010). Target hue had no effect in a combined decision and memory design (Blase et al., 2024). Background colors associated with different self-reported arousal scores did not improve short-term discrimination of angry faces (Langeslag et al., 2011). This directly separated subjective arousal from memory performance. An e-commerce website-color family produced mostly null results across 15 rows, with mixed directions elsewhere (J.-É. Pelet & Papadopoulou, 2012). An interface family that varied hue, saturation, and lightness produced null, negative, and mixed rows without a consistent direction (Gnambs et al., 2010). One background-color family contained equal numbers of positive and negative rows (Prasetya, 2016). Another interface-hue family reported mostly null self-report outcomes (Heidig et al., 2015).

Five families contained mixed detected directions. All eight illumination-hue rows were mixed in one family (Knez, 1995). Another reported mixed object-hue effects across six rows (Puechbroussou & Rusinek, 2021). A background-color family combined positive and null effects (Sinclair et al., 1998). The only family with internally conflicting descriptive rows reported negative, null, and unclear outcomes for saturation, lightness, and brightness (Cömert & Samur, 2025). These rows remained descriptive and were excluded from quantitative analysis. Illumination luminance and object chroma also produced combinations of positive, null, or mixed rows (L. Chen et al., 2026; Thorstenson et al., 2021). Illumination hue yielded mainly positive effects with EEG measures, alongside null rows (Qu & Kato, 2026). Other families combined positive EEG and self-report results with one null row (Hosseini & Ghabanchi, 2022); positive object-color effects with null outcomes (Sivananthan et al., 2024); or positive, mixed, and null results for interface color coordinates (Pak et al., 2026). The earliest family already showed one detected, one null, and one unclear outcome for colored scenes (Farley & Grant, 1973).

#### 3.6.3. Memory Encoding Versus Later Stages

The relevant mechanism boundary is memory stage. Encoding differentiation, working-memory maintenance, and retrieval are separate processes, and studies placed color at different stages. Research on red and blue performance illustrates the distinction between encoding and retrieval. One family found task-dependent red and blue effects on detail-oriented and creative outcomes (Mehta & Zhu, 2009). In a representative contrast, approach-related anagrams were solved faster against blue than red backgrounds. The blue condition had M = 10.93 s and standard deviation (SD) = 5.51, while the red condition had M = 18.53 s and SD = 12.25 (d = 0.81, *p* < 0.01). Twenty-two of 31 rows were positive. A memory study that used red as a cue produced mainly negative and mixed results: 6 negative, 5 mixed, and 2 positive rows (Elliot et al., 2011). The same hue can therefore accompany worse performance when it signals avoidance at test.

The clearest stage-sensitive evidence came from a study that manipulated color at encoding and retrieval (Gnambs, Appel, & Kaspar, 2015). Color at retrieval reduced declarative knowledge scores relative to gray (B = −1.33, β = −0.29, *p* < 0.05). Participant sex further moderated the encoding color-by-test color interaction. Of 27 rows, 12 were null, and 12 unclear. A workplace-lighting family manipulated lighting during study and testing (Hawes et al., 2012). It reported 13 positive, 3 negative, and 5 null rows. These illumination results show detectable memory differences but do not identify hue as the active property.

Color may therefore affect encoding, retrieval, or the match between them. Direction depended on memory stage, the emotional relation between color and content, and participant moderators. In emotional-design studies, color also changed salience, layout, anthropomorphism, or instructional signaling (Le et al., 2018; Liew et al., 2022; Plass et al., 2014; Um et al., 2012). Any memory advantage in those studies cannot be attributed to color alone. Direct mediator tests were uncommon, and no family manipulated a proposed pathway independently of color.

#### 3.6.4. Candidate Mechanisms, Boundary Conditions, and Reporting Gaps

Mechanism labels were multi-label indicators of compatibility rather than established mediation. Affective congruency appeared in 14 families and arousal in 12. Both recurred across independent families. Memory prioritization appeared in 9 families, perceptual salience in 8, and embodied or metaphorical mapping in 2 (Jung et al., 2011; Sivananthan et al., 2024). Eight families had no recorded mechanism. Affective congruency and perceptual salience often occurred in the same family (Chai et al., 2019; Kuhbandner & Pekrun, 2013; Thorstenson et al., 2021; Y. Wang et al., 2021). Emotionally congruent colors were usually also salient, and few designs varied the two independently.

Effects differed by property, task role, comparator, and moderator. Hue dominated the evidence. Saturation, lightness, brightness, chroma, and luminance each appeared in no more than nine families, leaving sparse and heterogeneous evidence for non-hue dimensions (Cömert & Samur, 2025; Gnambs et al., 2010; Thorstenson et al., 2021). Background and illumination studies contained the highest proportions of null and mixed rows (Knez, 1995; Olsen, 2010; J.-É. Pelet & Papadopoulou, 2012). Target- and object-color studies detected differences more often (Cipollone, 2024; Kawasaki & Yamaguchi, 2012; Kuhbandner & Pekrun, 2013). This pattern is compatible with an encoding-feature account. It does not exclude arousal. Comparators also estimated different effects. Red versus gray and red versus blue are not equivalent. One large family also found sex moderation of the encoding-by-test interaction (Gnambs, Appel, & Kaspar, 2015).

Reporting gaps prevented a formal synthesis. Many reports omitted effect estimates or gave only direction and significance. Sixteen rows remained unclear, and one family contained unresolved internal conflicts (Cömert & Samur, 2025). Physiological evidence was rare. When EEG was collected, it was not always linked to a particular memory stage (Chai et al., 2019; Hosseini & Ghabanchi, 2022; Qu & Kato, 2026). The evidence supports effects under particular combinations of property, role, stage, and moderator. It does not support a general claim that color improves memory, and the tasks and comparators were too heterogeneous for pooling.

Contributing reports: (Ab Jalil et al., 2021; Blase et al., 2024; Chai et al., 2019; L. Chen et al., 2026; Cipollone, 2024; Cömert & Samur, 2025; Elliot et al., 2011; Farley & Grant, 1973; Gnambs et al., 2010; Gnambs, Appel, & Kaspar, 2015; Hawes et al., 2012; Heidig et al., 2015; Hosseini & Ghabanchi, 2022; Jung et al., 2011; Kawasaki & Yamaguchi, 2012; Knez, 1995; Kuhbandner & Pekrun, 2013; Langeslag et al., 2011; Le et al., 2018; Liew et al., 2022; R. Lucas, 2022; Mehta & Zhu, 2009; Olsen, 2010; Pak et al., 2026; J.-É. Pelet & Papadopoulou, 2012; Plass et al., 2014; Prasetya, 2016; Puechbroussou & Rusinek, 2021; Qu & Kato, 2026; Sinclair et al., 1998; Sivananthan et al., 2024; Thorstenson et al., 2021; Um et al., 2012; Y. Wang et al., 2021).

### 3.7. Environmental Lighting and Context

The environmental lighting and context theme contained 64 study families and 1760 result rows. It had the largest extraction volume, although it did not have the largest family count. These families represented 21.8% of all included families and covered five decades. The studies ranged from early office-lighting experiments (De Lucia, 1975) to glazing, classroom, and virtual-environment studies published through 2026 (T. Li et al., 2026; Peng et al., 2026; Sezer & Olguntürk, 2026; Shehab & Durmus, 2026; M. Xia et al., 2026). Settings included classrooms, offices, hospitals and care facilities, retail and exhibition spaces, restaurants, driving simulators, virtual reality, and full-scale lighting installations.

Most studies embedded color in an environment rather than a discrete stimulus. Sixty-one of 64 families therefore had an “other” task label. Here, that category covered alertness and vigilance, sustained attention, creativity such as Torrance Tests of Creative Thinking, emotional working memory, risk decisions, driving, and locomotion in virtual space. Five families also used memory tasks; two included decision or classification; and one each included go/no-go, recognition, and visual search. These studies rarely isolated one cognitive process. Their results mainly concern context-dependent changes in affect, appraisal, physiology, and performance.

Hue appeared in 37 families and named colors in 35. Color coordinates appeared in 22, luminance in 15, chroma in 12, lightness in 10, brightness in 6, and saturation in 6. Only two families were explicitly coded for correlated color temperature (K. Choi & Suk, 2020; Shishegar & Boubekri, 2022). Another 17 used other or insufficiently specified attributes. Color functioned as illumination in 36 families; scene color in 26; and a background—usually a wall—in 20. It appeared as an object in four, other in three, an interface in two, and a cue in one.

This role distribution separates two types of study. About half manipulated lighting itself by changing spectral composition, illuminance, or color temperature. Examples include correlated-color-temperature studies (K. Choi & Suk, 2020; Lasauskaite et al., 2023; Shishegar & Boubekri, 2022), a glazing-transmittance experiment (X. Chen et al., 2019), and a classroom-illumination contrast (Quiles-Rodríguez & Palau, 2024). Other studies changed the colored room, wall, or scene around an unrelated task (AL-Ayash et al., 2016; Hygge & Knez, 2001; Knez & Niedenthal, 2008; Stone & English, 1998). The first design estimates physical exposure to light. The second estimates chromatic context. The charted evidence does not justify combining them.

The measurement profile was broad. Self-report appeared in 56 families, behavior in 54, autonomic measures in 16, EEG in 9, eye tracking in 5, pupillometry in 3, and ERP in 2. Multimodal studies often found different results at different response levels. A lighting condition could change affect, comfort, or appraisal without changing performance—or the reverse. Immersive scene hue and chroma produced mainly detected EEG, autonomic, and behavioral effects in one family (Llinares et al., 2021). Other families found scene-color effects on selected neural or pupillary measures without a uniform behavioral counterpart (Bekhtereva & Müller, 2017; Pilarczyk et al., 2020). A family that combined eye tracking, autonomic measures, behavior, and self-report also produced divergent outcomes (Batistatou et al., 2022). These results cannot be summarized as environmental color improving or impairing cognition. Direction depended on the property, role, comparator, and especially the response level.

Of the 1760 rows, 593 were positive, 203 negative, 727 showed no detectable difference, 168 were mixed, and 69 were unclear. Null results were the most frequent. Detected results clustered in selected families. A full-scale workspace study of glazing color and transmittance was the largest extraction in the review, with 188 rows (X. Chen et al., 2019). It contained 128 positive rows. Glazing type had a seven-level main effect on Positive and Negative Affect Schedule (PANAS) positive mood (*p* = 0.001), but directions differed across glazing levels, and 17 rows showed no difference. After a stressor, differently colored walls produced 47 positive rows among 117 (Du et al., 2025). Subjective stress fell during restoration relative to the stressor stage (*p* < 0.01).

Other detected-effect families covered illuminance and color temperature. One contributed 29 positive rows among 45 behavioral and self-report outcomes (Shishegar & Boubekri, 2022). Another contributed 30 positive rows among 57 autonomic, self-report, and memory outcomes (M. Xia et al., 2026). A scene-hue and brightness family produced 28 positive rows among 35 (Tantanatewin & Inkarojrit, 2018). An illumination study combining self-report, pupillometry, behavior, and autonomic measures produced 23 positive rows among 43 (Son & Yeom, 2026). A family that crossed illuminance with lighting color contributed 96 rows (Zhou et al., 2025). At 300 lx, blue-accent rather than white lighting had a small positive effect on Torrance Tests of Creative Thinking (TTCT) elaboration (Cohen’s d = 0.17, *p* = 0.039). The same family contained 20 negative and 39 null rows. Four of its 96 rows had verified source conflicts and were restricted to description. The other non-conflict rows remained eligible for quantitative assessment. Smaller families found effects of warm versus cool light on affect (Knez & Niedenthal, 2008), light-dependent changes in emotional working memory (Weigand et al., 2021), and color-temperature effects on cardiovascular effort (Lasauskaite et al., 2023).

Null-dominant families were equally prominent. A classroom-illumination study was the second-largest extraction, with 130 rows (Quiles-Rodríguez & Palau, 2024). Of these, 122 showed no detectable difference. One detected result was a higher net-attention score under orange than natural illumination, with means of 47.2 and 33.8, respectively (Dunn *p* = 0.039). Other families produced 46 null rows among 58 named-color environmental outcomes (Hulshof, 2013); 26 among 36 hue and brightness outcomes (van Hagen et al., 2009); 27 among 29 outcomes spanning behavior, self-report, EEG, and eye tracking (Segal et al., 2016); and 23 among 42 luminance outcomes (de Vries et al., 2018). Another study found no difference in five of six rows (Burattini et al., 2019).

The early office-color studies of Kwallek and colleagues were also mainly null. They varied wall hue, chroma, and lightness and found that performance and mood were largely insensitive to wall color (Kwallek & Lewis, 1990; Kwallek et al., 1996, 1988, 1997). Unclear or mixed rows outnumbered detected results. These null studies show that realistic chromatic variation often left cognition and affect unchanged.

Many families contained detected, opposing, and null results together. A classroom-color family measured emotion, heart rate, and reading performance (AL-Ayash et al., 2016). It produced 12 positive, 15 negative, 8 mixed, and 23 null rows. Another family crossed illuminance and light color under different initial emotional states (T. Li et al., 2026). It produced 15 positive, 16 negative, 8 mixed, and 46 null rows. In an exhibition-space image study, hue produced 24 positive, 18 negative, and 24 mixed rows (L. Hu et al., 2024). Total saccade count differed by hue level, reported as *p* = 0.00 in the source, and the direction varied across hues. This family also had verified conflicts within the source. Other families contained 8 mixed rows among 17 (Stone, 2001) or 13 among 27 (Kwallek et al., 1996). Across the theme, 50 of 64 families combined detected and null results, 9 had mixed detected directions, 1 was null-only, and 4 were positive-only. Within-family mixedness was the usual pattern.

Several designs show why the effects were tied to context. Office-color effects followed task demands and the meaning of the setting rather than hue alone (Stone & English, 1998). Kombeiz and colleagues likewise found that colored environmental features changed motivation or behavior only under particular task framing (Kombeiz, 2017; Kombeiz & Steidle, 2017). Illumination hue effects on self-report and behavior depended on saturation and lightness levels within the same design (von Castell et al., 2018). During anger induction, ambient chromatic interventions produced mainly negative or null cardiovascular and EEG-asymmetry results (Spiridon, 2017). A study that crossed luminance with memory and other tasks produced positive and mixed rows together (Hygge & Knez, 2001). Room color shifted appraisal in another family, although eight rows remained unclear (Cha et al., 2020). Retail, exhibition, and virtual studies (Afifi et al., 2024; Shehab & Durmus, 2026; Tantanatewin & Inkarojrit, 2018), as well as classroom studies (AL-Ayash et al., 2016; Quiles-Rodríguez & Palau, 2024), yielded setting-specific results. Cross-context transfer was not tested. Environmental color effects varied with physical exposure, the activity, and the occupant’s state. They also differed by response level.

Arousal was the most common candidate mechanism, mapped in 32 families. Attentional capture appeared in 10, perceptual salience in 8, and affective congruency in 5. Embodied or metaphorical mapping appeared in three, conflict modulation in one, and memory prioritization in one. Twenty-eight families had no recorded mechanism. Arousal accounts were compatible with changes in alertness, autonomic tone, and mood under lighting (X. Chen et al., 2019; Shishegar & Boubekri, 2022; M. Xia et al., 2026). The row-level evidence did not have one arousal direction. The same nominal manipulation produced positive, negative, and null results within and across families. Arousal therefore remains an interpretive frame rather than an established pathway. Environmental manipulations also combined illuminance, spectral composition, correlated color temperature, exposure duration, glazing transmittance, and scene content. Only 22 families reported explicit color coordinates.

These design and reporting features restrict interpretation. A hue-specific claim about illumination requires independent photometric and chromatic control, which most studies lacked. Only two families were explicitly coded for correlated color temperature. Exposure duration, adaptation time, and baseline occupant state were charted inconsistently. Because most tasks were labeled “other,” the theme alone cannot support process-specific claims about orienting, conflict resolution, or memory encoding under colored light. Eleven families contained verified source-conflict rows, including (L. Hu et al., 2024; T. Li et al., 2026; Zhou et al., 2025). Only the conflicting rows were restricted to descriptive synthesis. Non-conflict rows from these families remained eligible for quantitative assessment. More informative environmental studies would separate illuminance from chromaticity, report color coordinates and exposure parameters, and prespecify the expected response level.

Contributing reports: (Afifi et al., 2024; AL-Ayash et al., 2016; Barba et al., 2018; Barkmann et al., 2011; Batistatou et al., 2022; Bekhtereva & Müller, 2017; Burattini et al., 2019; Cha et al., 2020; C. Chen et al., 2025; S. Chen et al., 2023; X. Chen et al., 2019; Y. Chen et al., 2026; K. Choi & Suk, 2020; Chung, 2015; De Lucia, 1975; de Vries et al., 2018; Du et al., 2025; L. Hu et al., 2024; Hulshof, 2013; Hygge & Knez, 2001; A. Jiang et al., 2023; T. Jiang et al., 2022; Knez & Niedenthal, 2008; Kocaoğlu, 2015; Kombeiz, 2017; Kombeiz & Steidle, 2017; Kwallek & Lewis, 1990; Kwallek et al., 1996, 1988, 1997; Ladeira et al., 2025; Lan et al., 2021; Lasauskaite et al., 2023; T. Li et al., 2023, 2026; Y. Li, Fang, Qiu, & Wang, 2024; Lipnitsky & Polyak, 2026; Llinares et al., 2021; McDonnell et al., 2025; Michels et al., 2022; Öztürk et al., 2011; Peng et al., 2026; Pilarczyk et al., 2020; Quiles-Rodríguez & Palau, 2024; Ru et al., 2019; Segal et al., 2016; Sezer & Olguntürk, 2026; Shao et al., 2024, 2025; Shehab & Durmus, 2026; Shishegar & Boubekri, 2022; Son & Yeom, 2026; Spiridon, 2017; Stone, 2001; Stone, 2003; Stone & English, 1998; Tantanatewin & Inkarojrit, 2018; Ursuţiu et al., 2017; Uzun, 2017; Uzun & Yıldırım, 2018; van Hagen et al., 2009; von Castell et al., 2018; Weigand et al., 2021; West et al., 2010; M. Xia et al., 2026; Zhou et al., 2025).

### 3.8. Emotion and Physiological Response

The emotion and physiological response theme contained 36 study families and 372 result rows, or 12.2% of all families. No family contained internally conflicting descriptive rows, so all 372 rows entered the descriptive synthesis. The evidence covered more than two decades. It ranged from early psychophysiological work with colored objects (Hatta et al., 2002) to recent EEG and applied studies (Ávila-Muñoz et al., 2026; Bharti et al., 2026; Dou et al., 2026; Nambiar, 2026). The paradigms differed, but each examined affective processing through neural responses, peripheral physiology, subjective experience, or behavior. These response levels often diverged within a study. The synthesis therefore separates color properties and roles, direction patterns, response levels, and interpretive boundaries.

#### 3.8.1. Color Properties, Roles, Tasks, and Outcome Modalities

Hue appeared in 29 families and named colors in 17. Other properties were uncommon: saturation appeared in six families, lightness in five, color coordinates in four, chroma in two, luminance in two, and brightness in one. Several studies combined hue with these properties rather than isolating them (Z. Cao et al., 2024; Désiron & Schneider, 2024; Guo et al., 2022; L. Song et al., 2024). Color served as an object property in 11 families, a background in 9, an interface feature in 5, and a target in 4. Cue, illumination, and scene color each appeared in three families. In object- and target-color designs, color belonged to the affective stimulus, such as a face, picture, or emotional word (K. Hu et al., 2020a; Nakajima et al., 2015; Schindler et al., 2019). Background, scene, and illumination studies instead placed color around the task (Frühholz et al., 2009; Küller et al., 2009; Vandewalle et al., 2010). These roles estimate different effects.

Thirty-five families used tasks coded as “other.” These included viewing faces, pictures, films, rooms, and scenes, as well as multimedia interaction and spectral light exposure. Only two families included explicit decision or classification tasks (Vandewalle et al., 2011, 2010). The measurement profile distinguished this theme. Behavioral outcomes appeared in 25 families and self-report in 21. EEG appeared in 11, ERP in 8, autonomic measures in 7, eye tracking in 5, and pupillometry in 3. Fourteen families combined a physiological or neural measure with self-report or behavior (Bilucaglia et al., 2019; K. Hu et al., 2020a; Küller et al., 2009; X. Li et al., 2025; X. Wang et al., 2026). These multimodal designs allowed for direct comparisons across response levels.

#### 3.8.2. Supportive, Null, and Within-Family Mixed Results

Of the 372 rows, 137 were positive under the recorded orientations, 58 were negative, 109 showed no detectable difference, 50 were mixed, and 18 were unclear. Twenty-two families combined detected and null results, five had mixed detected directions, and nine were positive only. Detected color effects were common, but most families also contained null outcomes. The balance depended on the response level.

Several large families found affective differentiation by color. An interface-color family varied hue, lightness, and color coordinates across 50 rows (Wu et al., 2022). Color harmony correlated with pleasure under high-load scheme observations (r = 0.397, *p* < 0.001). The same family also contained 13 mixed and 14 null rows. Another family tested frames that were congruent or incongruent with synesthetic color–sound correspondences (Hochel et al., 2009). Congruent frames produced higher arousal ratings (M = 6.0) than incongruent frames (M = 4.2; *p* < 0.01). Its 33 rows also included 12 mixed and 7 unclear outcomes.

Applied stress studies also found selected effects. A blue immersive environment reduced subjective stress relative to a control environment (M = 83.19 versus M = 90.38; mean difference = −7.19, *p* < 0.001) (Y. Zhao et al., 2025). The family contained eight positive and seven negative rows. The theme’s second-largest family compared a patterned multicolored room with a gray room across 40 rows (Küller et al., 2009). Appraisal differed strongly, including perceived complexity at *p* < 0.001. Twenty rows, including several physiological and performance measures, showed no detectable difference. Positive-only patterns occurred mainly in small target- and object-color ERP families (Ma et al., 2020; Stefanics et al., 2018) and applied object- or background-color studies with one or two outcomes (Bharti et al., 2026; Nambiar, 2026; Raudonis et al., 2017; X. Wang et al., 2026; Zhang et al., 2023). Their small row counts partly explain the uniformly positive pattern.

Null and mixed results appeared in every modality. Under blue light, angry prosody slowed speaker-gender discrimination relative to neutral prosody (*p* = 0.012), while 12 of 19 rows were null (Vandewalle et al., 2011). In an early object and interface color study, 8 of 14 rows showed no difference, 4 were negative, and 2 were unclear (Hatta et al., 2002). An interface-hue family divided 11 rows into 5 positive and 5 negative outcomes (Nissen, 2020a). Film color and editing affected selected affective and neuroimaging outcomes, while 8 of 17 rows remained unclear (Z. Cao et al., 2024). Emotional-design color in multimedia affected some affective and performance measures, but 10 of 16 rows were null (Lang et al., 2025). Background hue produced equal numbers of positive, negative, and null rows across EEG, self-report, and behavior (Ávila-Muñoz et al., 2026). Color-photism work (Hochel et al., 2009) and a scene-color EEG family with 5 mixed rows among 12 (Dou et al., 2026) showed the same within-family heterogeneity. The central pattern was therefore strong dependence on outcome, condition, and response level.

#### 3.8.3. Neural, Autonomic, and Subjective Response Levels

Response level provides the main boundary in this theme. The eight ERP families illustrate differences in timing. Color-related changes appeared in N170, P3, early posterior negativity (EPN), or late positive potential (LPP) components. Early P1 effects were often absent or depended on context (Frühholz, 2008; Frühholz et al., 2009; Hasegawa et al., 2025; Nakajima et al., 2015; Schindler et al., 2019). Target hue produced positive ERP and EEG effects in 10 of 12 rows in one family (K. Hu et al., 2020a). Target lightness and brightness produced positive ERP outcomes in another (L. Song et al., 2024). Named object colors produced mainly negative ERP rows (Schindler et al., 2019). Background hue produced five mixed rows among seven (Frühholz et al., 2009). Across these heterogeneous tasks, color effects appeared more often at later evaluative stages than at early sensory stages. This pattern does not establish a stage-specific mechanism.

The clearest differences emerged when studies measured physiology with behavior or self-report. Angry voices under blue light produced stronger neural responses without changes in gender-classification accuracy or reaction time (Vandewalle et al., 2010). Another colored-illumination family found physiological differences, while five of eight other rows were null (Alkozei et al., 2016). A hue study reported positive pupil, autonomic, and EEG rows but did not include behavioral outcomes (Bilucaglia et al., 2019). A comparatively rare family showed positive background-hue effects across self-report, eye tracking, autonomic measures, and behavior (X. Wang et al., 2026).

Appraisal studies showed similar separation. Colorful and gray rooms differed strongly in subjective appraisal, while activation and several performance or physiological outcomes did not differ (Küller et al., 2009). A background and interface color family produced positive self-report effects in 13 of 19 rows, with one mixed row (Nissen et al., 2024). The study did not establish physiological correlates of those experiences. Pupillometry and eye tracking added measure-specific findings. Interface color preferences were paired with pupil and gaze measures (Wu et al., 2022). Background hue affected pupil and gaze outcomes alongside self-report, with one negative row (X. Li et al., 2025). Autonomic effects of colored objects and cues appeared in some families (Bharti et al., 2026; Désiron & Schneider, 2024; Hochel et al., 2009) but were mainly null in others (Hatta et al., 2002).

An ERP amplitude difference does not measure experienced emotion. Autonomic arousal alone does not establish affective experience, and preference does not locate the processing stage. Behavioral, subjective, neural, and peripheral outcomes diverged within many multimodal families (Ávila-Muñoz et al., 2026; Z. Cao et al., 2024; Küller et al., 2009; Vandewalle et al., 2010). When response levels converged, studies usually described the correspondence without testing mediation (Guo et al., 2022; Ramos-Nuñez et al., 2018; X. Wang et al., 2022; Zhang et al., 2023). The evidence therefore cannot identify which level carries a causal pathway.

#### 3.8.4. Candidate Mechanisms, Boundary Conditions, and Reporting Gaps

Mechanism labels indicate compatibility rather than established mediation. Arousal appeared in 17 families and perceptual salience in 15. Both recurred across independent families. Attentional capture appeared in 12 families, affective congruency in 9, and conflict modulation in 4. Embodied or metaphorical mapping and memory prioritization each appeared in one family (Guo et al., 2022; X. Li et al., 2025). Five families had no recorded mechanism (Z. Cao et al., 2024; Hatta et al., 2002; Küller et al., 2009; Nissen, 2020a; Schindler et al., 2019). Arousal, salience, and capture often co-occurred in one family (Dou et al., 2026; Frühholz et al., 2009; K. Hu et al., 2020a). Arousing colors were usually also salient, and few designs varied these properties or pathways independently.

Reported patterns varied across color properties, response levels, comparators, and contexts. Hue accounted for most evidence. Saturation, lightness, chroma, luminance, and brightness each appeared in no more than six families, often with null or unclear rows (Z. Cao et al., 2024; Guo et al., 2022; L. Song et al., 2024). Subjective appraisal and late ERP components detected differences more often than autonomic, pupillary, or behavioral outcomes. Divergence across levels was common (Alkozei et al., 2016; Küller et al., 2009; Vandewalle et al., 2011, 2010). Multicolored versus gray, congruent versus incongruent, and hue versus hue contrasts estimate different effects. An immersive application (Y. Zhao et al., 2025) also differs from a single-stimulus ERP paradigm (Ma et al., 2020; Stefanics et al., 2018).

Incomplete reporting restricted synthesis. Many reports gave only direction and significance or omitted reconstructable effect estimates. For example, the complexity contrast in Küller et al. (2009) had no reconstructable estimate. Eighteen rows remained unclear, mainly in Hochel et al. (2009) and Z. Cao et al. (2024). Several studies reported physiology without linking it to a specific affective stage (Dou et al., 2026; Hasegawa et al., 2025; Nakajima et al., 2015). No family manipulated a proposed mediator independently of color. The evidence supports color-related modulation under particular combinations of property, role, and comparator. It does not support a general claim that color changes emotional experience, and the paradigms were too heterogeneous for pooling.

Contributing reports: (Alkozei et al., 2016; Ávila-Muñoz et al., 2026; Bharti et al., 2026; Bilucaglia et al., 2019; Z. Cao et al., 2024; Désiron & Schneider, 2024; Dou et al., 2026; Frühholz, 2008; Frühholz et al., 2009; Guo et al., 2022; Hasegawa et al., 2025; Hatta et al., 2002; Hochel et al., 2009; K. Hu et al., 2020a, 2020b; Küller et al., 2009; Lang et al., 2025; X. Li et al., 2025; Ma et al., 2020; M Nael, 2019; Nakajima et al., 2015; Nambiar, 2026; Nissen, 2020a; Nissen et al., 2024; Ramos-Nuñez et al., 2018; Raudonis et al., 2017; Schindler et al., 2019; L. Song et al., 2024; Stefanics et al., 2018; Vandewalle et al., 2011, 2010; X. Wang et al., 2022, 2026; Wu et al., 2022; Zhang et al., 2023; Y. Zhao et al., 2025).

### 3.9. Other Cognitive and Behavioral Tasks

The residual theme contained 17 study families and 286 result rows, or 5.8% of all families. Two families contained internally conflicting descriptive rows (A. N. Chen et al., 2018; G. Xia et al., 2021). These rows remained in the description only, leaving 282 non-conflict rows. The theme contains paradigms outside attention, perception, memory, emotion, and environmental context. They include creativity, time perception, online waiting, motor behavior, assistive cueing, broad performance batteries, and direct replications of color-priming claims. The studies range from an early applied performance family (Pressey, 1921) to recent research on time perception and interfaces (Chulvi et al., 2022; Kübel et al., 2021; G. Xia et al., 2021). Because the paradigms have no common estimand, they are grouped below by task.

#### 3.9.1. Color Properties, Roles, Tasks, and Outcome Modalities

Hue appeared in 14 families and named colors in 12. Brightness, color coordinates, and saturation each appeared in two, while chroma and lightness each appeared in one. Color served as a background in seven families; an object property in four; and illumination, target, or distractor in three each. Cue and interface roles each appeared in two. Sixteen families had an “other” task label. One family each was also labeled for creativity (Lichtenfeld et al., 2012), go/no-go (Luo et al., 2019), memory (Pressey, 1921), and decision or classification (Pressey, 1921). Behavioral outcomes appeared in 13 families and self-report in 10. Physiological measurement was rare. Two families used other physiological or imaging measures (Cooper et al., 1994; Stefanics et al., 2019), and one used autonomic recording (Pressey, 1921). The evidence is therefore mainly behavioral and subjective.

#### 3.9.2. Creativity, Time Perception, Waiting, and Performance Batteries

Of the 286 rows, 85 were positive under the recorded orientations, 52 were negative, 125 showed no detectable difference, 20 were mixed, and 4 were unclear.

The best-known positive result came from creativity research. Green backgrounds improved creative performance relative to other hues, with 7 positive and 11 null rows (Lichtenfeld et al., 2012). Mood and activation did not change, so the performance effect appeared without a measured affective mediator. Time-perception research was mostly null. A family that compared red and green Ganzfeld illumination contributed 41 rows, of which 25 were null (Kübel et al., 2021). Under red, a passage-of-time rating with volitional control correlated with duration judgment (r = −0.367, *p* = 0.030). The study used a Bonferroni threshold of *p* ≤ 0.001, so this result was coded as no detectable difference. Detected rows indicated relative duration overestimation under red. During online waiting, background color produced nine positive, two negative, and four null self-report rows (Gorn et al., 2004). This pattern was compatible with an arousal account of perceived waiting time.

Broad performance batteries yielded the weakest pattern. One of the theme’s two joint-largest families also contained a conflict within the source (G. Xia et al., 2021). It contributed 41 rows on background and interface hue, chroma, lightness, and coordinates. Thirty were negative under the recorded orientations. General task performance was slower against purple than a reference white background (least significant difference (LSD) mean difference = −15.01, *p* = 0.032). The overall pattern was task- and color-specific rather than a general performance cost. Another family found an affect valence-by-color interaction for PANAS scores (*p* = 0.006) amid 16 null rows among 26 (Williams et al., 2011). The review’s oldest family contributed 25 illumination-hue and brightness rows on performance, autonomic measures, and self-report (Pressey, 1921). None reached conventional significance, anticipating the null-heavy pattern found in recent work.

Applied object- and cue-color studies added mixed evidence. Object color in motor behavior produced seven positive and seven null rows (Rook, 2014). Colored cues and objects produced positive and null outcomes with physiological measures (Cooper et al., 1994). The second conflict-bearing family reported mainly null self-report outcomes for background and interface hue, with 6 of 11 rows null (A. N. Chen et al., 2018).

#### 3.9.3. Learned Color Effects, Priming, and Replication Boundaries

Another group examined learned or task-relevant color relations. One family used named colors as response features and distractors in a go/no-go task (Luo et al., 2019). It contained 13 positive, 1 negative, and 2 mixed rows, with color serving as an explicit task-relevant feature. Target hue produced eight positive and four null rows in another family (K. Hu, 2019). The only positive-only family found affective consequences of attending to hue and saturation across two rows (Goolsby et al., 2009). A target and distractor study reported one positive and one null hue row (Stefanics et al., 2019). These findings are compatible with learned relevance and selection history, but each came from a single family and seldom isolated the mechanism.

Replication provided the clearest limit. One study directly replicated the red-avoidance and blue-approach priming interaction predicted from Mehta and Zhu (2009) (Steele, 2014). The predicted interaction did not replicate. Red, white, and blue background still had a mixed-direction main effect on anagram solution time ($\eta^{2}$ = 0.015, *p* = 0.03). Ten of 22 rows were null. Thus, an effect found in one task can fail in a closely related design. Isolated positive families (Goolsby et al., 2009; K. Hu, 2019; Luo et al., 2019) do not support a general claim.

The other families support the same caution. An assistive-cueing family was null across all 13 rows and was the only null-only family (Larsson & von Stumm, 2015). Named background colors produced mixed directions in four of six rows (Ikeda, 2020). Another named-color family contained two positive, one mixed, and four null rows (Chulvi et al., 2022). Fourteen of 17 families combined detected and null results. One had mixed detected directions, one was null only, and one was positive only.

#### 3.9.4. Candidate Mechanisms, Boundary Conditions, and Reporting Gaps

Mechanism mapping was sparse. Seven families had no recorded mechanism (A. N. Chen et al., 2018; Chulvi et al., 2022; Ikeda, 2020; Larsson & von Stumm, 2015; Lichtenfeld et al., 2012; Pressey, 1921; G. Xia et al., 2021). Five used other specified mechanisms (Goolsby et al., 2009; K. Hu, 2019; Luo et al., 2019; Rook, 2014; Williams et al., 2011). Arousal and perceptual salience each appeared in three families. Memory prioritization appeared in two (Cooper et al., 1994; Stefanics et al., 2019). Each account therefore rests on one to three families using different tasks. None formed a repeated organizing pattern within the theme.

The theme has several strict boundaries. Its tasks do not share an estimand: duration judgments, anagram solutions, motor kinematics, wait-time ratings, and priming interactions measure different quantities. A directional synthesis across them is not warranted. Color-priming also failed under a close replication (Steele, 2014), which limits generalization from isolated positive families (Goolsby et al., 2009; K. Hu, 2019; Luo et al., 2019). Reporting further restricted reconstruction. Two families had unresolved internal conflicts (A. N. Chen et al., 2018; G. Xia et al., 2021). The oldest family predates modern inferential-reporting conventions (Pressey, 1921). Nearly half of the theme had no mechanism label. These studies identify claims, replications, and refutations outside the major paradigms. They do not support pooling or a common direction.

Contributing reports: (A. N. Chen et al., 2018; Chulvi et al., 2022; Cooper et al., 1994; Goolsby et al., 2009; Gorn et al., 2004; K. Hu, 2019; Ikeda, 2020; Kübel et al., 2021; Larsson & von Stumm, 2015; Lichtenfeld et al., 2012; Luo et al., 2019; Pressey, 1921; Rook, 2014; Steele, 2014; Stefanics et al., 2019; Williams et al., 2011; G. Xia et al., 2021).

### 3.10. Cross-Theme Mechanisms and Reporting Completeness

#### 3.10.1. Candidate Mechanisms as an Organizing Framework

Candidate mechanism labels were multi-label descriptors assigned during extraction. A family could fit several accounts, so the counts exceed 294 when summed. A label records compatibility between a design and an explanation. It does not establish mediation or a causal chain. Across the corpus, arousal appeared in 95 families, affective congruency in 77, perceptual salience in 69, attentional capture in 50, conflict modulation in 22, embodied or metaphorical mapping in 19, and memory prioritization in 17. Another 41 families had an “other” label, including 22 for which it was the only mechanism category. Eighty-five families had no recorded mechanism. One family also carried an “unclear” placeholder, which was excluded from the recurrent categories shown in Figure 6. These reports usually did not articulate or manipulate a mediator. Most studies measured outcomes without changing the proposed mediator, so the labels serve only to organize the evidence. Use of the same design logic by at least two independent families defined a repeated organizing pattern. This criterion did not establish the mechanism. A single family was described only as consistent with an account.

Arousal was the most common label, appearing in 95 families. It was concentrated in environmental lighting and context (32 families), emotion perception and decision-making (18 families), and emotion and physiological response (17 families). An arousal account predicts that colored light or surroundings change subjective state, autonomic activity, or cortical response, which then affects behavior. A direct test therefore needs a state or physiological measure alongside the outcome. Few families met this requirement. The closest designs combined colored illumination with autonomic or EEG recording (Ciccone, 2018; Küller et al., 2009; Zhou et al., 2025), Ganzfeld stimulation with duration judgments (Kübel et al., 2021), or short-wavelength light with non-visual physiology (Vandewalle et al., 2011). Their outcomes and comparators were too different for one arousal estimate. Many other families used self-report alone, which is also compatible with demand characteristics or evaluative labeling.

Affective congruency appeared in 77 families and was most common in emotion perception and decision-making (31 families). It predicts faster, more accurate, or more positive processing when the emotional connotation of a color matches the stimulus, judgment, or response. A direct test compares matched and mismatched emotional relations while holding color perception constant. Judgment and classification tasks most often provided this contrast. Examples include brightness-valence response mappings (Meier et al., 2004), chromatic valence categorization (Kawai et al., 2020), recognition of affectively matched colored materials (Mammarella et al., 2016), and emotional Stroop studies in which ink color agreed or conflicted with word valence (Perry & Henik, 2013; Xu & Zhang, 2019). Congruency effects recurred in judgment and lexical decision. The same families often produced null secondary outcomes, and facilitation did not extend to unrelated tasks.

Perceptual salience appeared in 69 families. This account attributes effects to physical distinctiveness, including saturation, luminance contrast, or singleton status, rather than emotional meaning. A direct test requires equal luminance and local contrast or a salience-matched control. Without these controls, salience and meaning remain confounded. Salience was most common in attention and cognitive control (19 families). Examples included colored targets or distractors competing during visual search (Santos et al., 2010) and chromatic detection cues combined with autonomic recording (Domínguez-Borràs et al., 2013). Only 186 of 294 families reported luminance control across the corpus (Section 3.10.2). Many salience-labeled families therefore lacked the comparisons needed to distinguish this account.

Attentional capture appeared in 50 families. It predicts that color draws or holds attention during competition or when cue validity changes. A direct test requires competition, cue validity, or distractor interference. ERP studies supplied the clearest evidence. Colored singleton distractors changed attention-related components during search (Moran & Moser, 2015). Combined eye tracking and ERP in emotional Stroop localized color-related interference to early selection (Xu & Zhang, 2019). These families tested capture directly. Most other families inferred capture from reaction-time costs, which could also reflect response interference.

Conflict modulation appeared in 22 families. It predicts that color changes interference or inhibition in Stroop, flanker, Simon, or go/no-go tasks. The account therefore requires a reported interference or inhibition estimand. Nine attention and cognitive-control families and seven emotion perception and decision-making families met this structural condition. Response-compatibility variants were classified here or under residual explanations according to the report’s framing (Goolsby et al., 2009; Huang et al., 2022). Mixed and null interference effects were common. Conflict modulation therefore did not form a consistent repeated organizing pattern.

Embodied or metaphorical mapping appeared in 19 families. These accounts connect darkness with negativity, lightness with positivity, or red with danger and avoidance. They were concentrated in evaluative judgments (Lunardo et al., 2021; Meier et al., 2015, 2004; Okubo & Ishikawa, 2011). The studies often manipulated brightness or hue as a cue and measured classification. They directly tested the proposed mapping in a narrow sense but did not separate conceptual metaphor from learned affective association. Memory prioritization appeared in 17 families, including 9 in memory and learning. It predicts preferential encoding or retrieval of emotionally or perceptually distinctive colored items. Examples included EEG-assisted memory tasks with colored targets (Chai et al., 2019; Kawasaki & Yamaguchi, 2012). Both accounts rest on small, task-specific groups of families and remain candidate explanations.

The labels define the evidence requirements more clearly than they resolve the mechanisms. Salience requires control of luminance and local contrast. Congruency requires matched and mismatched emotional relations. Capture requires competition or cue-validity contrasts. Arousal requires state or physiological measures, and conflict requires an interference or inhibition estimate. Many families met only one requirement, and several met none. Multi-label families were compatible with more than one account by design. The same red stimulus could be arousing, salient, attention-capturing, or threatening depending on the task. Most studies lacked the within-study contrasts needed to choose among these explanations. No mechanism can be claimed as a shared causal pathway across themes. Figure 6 maps the distribution of candidate labels by primary theme and by measurement modalities recorded in the same result row.

#### 3.10.2. Reporting Completeness

Reporting completeness was assessed at the study-family level. A field counted as reported when at least one report in the family provided usable information (Section 2.10). Basic stimulus documentation was common. Display equipment or presentation medium was reported in 293 of 294 families (99.7%), and concrete color values were reported in 264 (89.8%). Information needed to reconstruct a contrast or effect was less complete. A color space was stated in 192 families (65.3%), luminance control in 186 (63.3%), and contrast control in 150 (51.0%). Randomization was reported in 251 families (85.4%) and counterbalancing in 195 (66.3%). Blinding appeared in 78 (26.5%), a sample-size rationale in 89 (30.3%), and missing-data handling in 180 (61.2%). Funding or support information was reported in 126 families (42.9%). Conflict-of-interest or financial-relationship information was reported in 91 families (31.0%). Figure 7 summarizes completeness by thematic domain and shows joint documentation of related field groups. Missing information on color space, luminance, contrast, and sampling limited stimulus reconstruction and effect-size recovery. These gaps also affected the quantitative gates in Section 3.11.

Funding or support information was available for 126 families. Recorded sources supported 107 families, and one thesis acknowledged personal financial support. Another 17 families contained explicit no-funding statements, sometimes limited to external or specific grants. One family mentioned funding without naming the source in the charted field. Company support, including material support, appeared in nine families. Conflict-of-interest or financial-relationship information was available for 91 families. Of these, 86 declared no competing interests and three disclosed specific relationships involving financial support, patents, or professional and commercial interests. Two further families disclosed financial relationships: one reported Carl Zeiss support, supplied lenses, and a company affiliation; the other reported Philips Lighting financial support in a disclaimer. No separate conflict-of-interest declaration was identified in either report. Funding information was unavailable in 168 families, and conflict-of-interest or financial-relationship information was unavailable in 203. Unavailable charted information does not establish that an original report omitted a statement.

### 3.11. Meta-Analysis Feasibility

No pooled meta-analysis was performed. The protocol made pooling conditional on metric and homogeneity gates rather than the amount of available data. Figure 8 shows these gates, and Table 3 traces every result row. The descriptive synthesis used 4923 rows. Excluding 44 source-conflict rows left 4879 non-conflict candidates before metric assessment. The question was whether these effects could be weighted, grouped, and pooled without combining different estimands or treating dependent results as independent.

The metric gate reduced this pool. Directly weightable effects were available for 569 rows from 43 study families. Of these, 409 reported an effect with sampling variance through a standard error or confidence interval. Another 160 reported a correlation and sample size, allowing for derivation of Fisher-*z* variance. A further 1190 rows contained test statistics that might be converted under design-specific assumptions. The review did not impose those assumptions. Among the remaining rows, 566 reported design-specific effects without a portable metric, 375 reported direct effects without usable variance, and 2179 lacked enough quantitative information for reconstruction. Directly weightable effects therefore comprised 569 of 4879 candidates (11.7%). Including the 1190 potential conversions as an upper bound would yield 1759 of 4879 rows (36.1%). Those conversions were not admissible and did not enter the direct-effect gate.

The homogeneity gate removed the remaining option for pooling. A cluster needed at least three non-overlapping families with the same color contrast, outcome, and estimand, along with compatible designs and manageable dependence. Only one coarse cluster reached three families with directly weighted effects. Three was also the maximum number of directly weighted families in any coarse cluster. Manual review found that this cluster failed every homogeneity criterion. It combined correlations with mean differences, mixed unrelated environmental and affective outcomes, and covered 230 distinct color contrasts. One family supplied 138 of the cluster’s 157 direct rows (X. Chen et al., 2019). A pooled result would therefore have been dominated by dependent effects from one experiment. No homogeneous poolable cluster remained.

The final analysis used descriptive evidence mapping and structured narrative synthesis. Pooling without a multilevel dependence model would count correlated results from one family as independent. It would also combine correlations, mean differences, and ordinal judgments that do not share an estimand. The reporting gaps in Section 3.10.2 would require unverifiable assumptions about stimulus properties for many reconstructed effects. Meta-analysis will become feasible only when studies use common contrasts and outcomes, report sampling variances, and document colorimetric properties.

## 4. Discussion

### 4.1. Principal Findings

This scoping review mapped 299 unique reports published from 1921 to 2026. The reports formed 294 study families and 4923 descriptive result rows. The initial database search yielded 155 reports. Three rounds of citation searching added 143 unique reports: 90 from CCA-01/02, 50 from CCA-03, and 3 from the final backward search (CCA-04). The pre-submission update added one report and seven result rows. Citation searching increased the representation of older studies, applied research, and replications that the database queries had missed. The update left the main interpretation unchanged and added another example of task-dependent interface effects.

The themes were unevenly represented. Emotion perception and decision-making was the largest theme, with 97 families and 1620 rows. Most task-level evidence therefore concerned judgment, classification, and choice. Evidence on attention and cognitive control made up a smaller share. Citation searching produced the largest absolute increase in environmental lighting and context. This theme contained 64 families and the largest extraction volume, at 1760 rows. One full-scale workspace study contributed 188 rows (X. Chen et al., 2019). Attention and cognitive control, the focal theme, contained 46 families and 479 rows. These comprised 18 core cognitive-control families and 28 general-attention families. This theme is central because it has a clear synthesis gap. The latest systematic review of color and emotion excluded task-level cognitive effects (Jonauskaite & Mohr, 2025). Our map shows that this excluded literature exists, although it remains sparse and heterogeneous.

Variation across tasks and contexts was the main descriptive pattern. Hue was manipulated in 208 families, and named colors were manipulated in 168. Saturation appeared in 46 families, lightness in 41, brightness in 26, and luminance in 23. Reported directions varied across color roles, task demands, emotional relations, comparators, and outcome modalities. Among the 294 families, 210 contained both detected and null results. Only 32 were positive-only under the recorded outcome orientations, and 13 were null-only. These descriptive categories cannot establish consistent benefit or impairment across contexts for red, blue, green, or another named color. Findings changed across tasks, comparators, and response levels. A close replication of an influential color-priming claim also produced a null result (Steele, 2014), whereas the original study reported the predicted effect (Mehta & Zhu, 2009). Reports from achievement, lighting, interface, and conflict paradigms also described different patterns for red (Ciccone, 2018; Elliot et al., 2007; Hao & Wang, 2026; Kang et al., 2022; Michaelis, 2013).

The family-level unit of analysis reveals another feature of this literature. Large experiments generated many rows, so row volume differed sharply from the amount of independent evidence. The workspace study alone contributed 188 rows (X. Chen et al., 2019), and several other families contributed dozens of contrasts. Each still counted as one family in prevalence estimates. This rule prevented studies with many outcomes from dominating the synthesis. It also affected the meta-analysis feasibility audit because one experiment supplied most direct effects in the largest apparently poolable cluster (Section 3.11). Evidence volume and independent replication must therefore remain separate quantities.

### 4.2. Ecological Validity and Practical Relevance

The practical relevance of emotional attention and cognitive control depends on context. Color is embedded in warning labels, interfaces, educational materials, rooms, and illumination. Real settings rarely present color as an isolated stimulus. A useful effect must change detection, disengagement, interference, or response selection enough to affect the intended task. This criterion connects laboratory findings to practice without presuming transfer.

Applied reports described differences across outcomes. Website color schemes changed fixations, recall, and ratings of pragmatic quality. Full-color warnings increased gaze duration without a detected change in beliefs or intentions (Byrne et al., 2019; Venni & Bétrancourt, 2020). Dynamic office lighting changed facial-affect ratings and eye-contact avoidance. The study detected no difference in concentration or N-back performance (De Ruyter & Van Dantzig, 2019). A social robot’s headband color changed attention duration among autistic children. Selected colored overlays also interacted with diagnosis in their effects on reading speed and completion time (Fang et al., 2026; A. Ludlow et al., 2008). These task-specific findings do not establish a general functional benefit.

The 64-family environmental theme covered classrooms, offices, hospitals, retail and exhibition spaces, restaurants, driving simulators, virtual environments, and full-scale lighting installations. Reported results varied across settings and across self-report, physiological, and performance measures. Researchers and designers should validate color in the target population, device, illumination, and use context. Applied tests should include functional outcomes. Their comparison conditions should separate chromatic content from luminance, contrast, expectancy, and learned response mappings.

### 4.3. Relationship to General Color–Emotion Associations

The context-free synthesis of color–emotion correspondences addressed a different question (Jonauskaite & Mohr, 2025). It found robust associations in explicit judgments and excluded cognitive effects within tasks. We mapped reports in which color acted as a target, cue, background, object property, interface feature, scene, or illumination condition. The mapped task reports do not establish whether abstract associations translate consistently into attention, control, memory, physiological, or decision outcomes. Such associations may support many experimental paradigms, but they cannot, by themselves, explain task-level effects.

Studies based on color–emotion associations reported different patterns across tasks. Brightness-valence compatibility appeared in some judgment and response-selection tasks (Huang et al., 2018, 2022; Lakens et al., 2013). Related comparisons appeared in two other families (Meier et al., 2015, 2004). In the largest family, 6 of 46 rows reached significance and 25 showed no detectable difference (Meier et al., 2004). Some achievement studies reported red-related performance effects (Elliot et al., 2007; Maier et al., 2008). A comparable family was entirely null (Smajic et al., 2014), and a close replication did not reproduce the predicted red–blue priming interaction (Steele, 2014). Independent families repeatedly linked facial redness and pallor to expression judgments (Kato et al., 2022; Minami et al., 2018; Nakajima et al., 2017). Even these designs could not separate low-level discriminability from affective diagnosticity. These reports describe task-specific patterns consistent with color-emotion associations. They do not justify transfer from association to performance.

General association accounts also combine outcomes that behave differently. Self-reported experience, measured in 174 families, often diverged from behavioral outcomes within the same study (Küller et al., 2009; Langeslag et al., 2011; Lichtenfeld et al., 2012). Two families reported neural differences without statistically detected behavioral differences (Iwakiri et al., 1999; Vandewalle et al., 2010). Another reported a neural difference but no behavioral outcome (T. Zhu et al., 2025). Studies described cross-modal convergence but did not test it as mediation (X. Wang et al., 2026). Claims should therefore distinguish abstract association, experienced emotion, attentional orienting and disengagement, perceptual salience, conflict and inhibition, response selection, memory encoding and retrieval, and neural or autonomic response. An effect at one level does not establish change at another level.

### 4.4. Mechanistic Interpretation

Seven extracted mechanism labels organized the evidence. Arousal was recorded for 95 families, affective congruency for 77, perceptual salience for 69, and attentional capture for 50. Conflict modulation appeared in 22 families, embodied or metaphorical mapping in 19, and memory prioritization in 17. No extracted mechanism label was recorded for 85 families. Each label requires a different test. Arousal predicts a broad state change and calls for physiological or state covariates. Congruency predicts facilitation when color matches the emotional material and calls for a matched-versus-mismatched interaction. Salience predicts effects that follow physical discriminability and requires controls for luminance and contrast. Capture predicts selection under competition and requires a contrast based on competition, cue validity, or distractor interference. Conflict modulation predicts a change in a prespecified interference or inhibition estimand. Embodied mapping predicts metaphor-consistent evaluative shifts. Response compatibility is an additional task-level account. It predicts priming of a compatible or incompatible response channel. A single stimulus can be arousing, salient, congruent, and metaphorically loaded at the same time. Designs that manipulated or measured the proposed mediator were rare, so most studies could not distinguish these accounts.

We treated each mechanism label as compatibility between a study design and an explanatory account. The label did not establish that the study had tested a mechanism. Only a minority of families used the necessary discriminative comparison. The distractor-cost and anxiety-regression contrasts of Moran and Moser (2015) and the eye-tracking and ERP design of Xu and Zhang (2019) tested capture directly. The congruency-proportion analyses of Huang et al. (2022) tested strategic conflict modulation. The luminance-matched emotional Stroop task of Kang et al. (2022) separated hue from luminance. These labels follow directly from the recorded contrasts. Other labels came from authors’ interpretations, so we retained them only as candidate explanations. We described an account as a repeated organizing pattern when at least two independent families used the same design logic. Facial-color sensitivity in expression judgments and brightness-valence congruency met this standard. Single-family findings were described only as consistent with an account. The evidence does not establish a shared causal pathway across themes.

Three studies show why this restraint is necessary. Buechner et al. (2014) found a color × expression × target-face interaction for attentional adhesion without a color main effect. The effect was therefore tied to a specific emotional relation. Vandewalle et al. (2010) reported neural differences under blue light during affective prosody processing without a statistically detected behavioral difference. Any pathway claim then depends on the response level being measured. In the 46 rows reported by Meier et al. (2004), a few brightness-valence compatibility effects appeared within a predominantly null pattern. Those effects depended on particular stimulus–response assignments. These studies locate processing boundaries, but none identifies a general mechanism.

Differences between measurement modalities provide the most useful process evidence now available. ERP and EEG measures located color-related modulation in attentional and evaluative components (Frühholz et al., 2009; K. Hu et al., 2020a; Nakajima et al., 2015). Eye tracking located effects on allocation and adhesion (Buechner et al., 2014; W. Jiang & Liu, 2025). Autonomic and pupillary measures indexed state changes under colored illumination (Alkozei et al., 2016; Bilucaglia et al., 2019). Self-report measured experienced or evaluated states, which often differed from the other modalities (Küller et al., 2009). Cross-modal convergence strengthens a process claim, whereas divergence narrows its scope. Mechanistic claims should stay at the measured level. Physiological modulation alone does not establish cognitive benefit.

### 4.5. Methodological Implications

The evidence was heavily imbalanced across color dimensions. Hue appeared in 208 families and named colors in 168. Saturation appeared in 46, lightness in 41, brightness in 26, and luminance in 23. The literature mainly shows *which* color was presented. It says less about *how saturated, light, or bright* that color was. These studies do not support the estimation of hue-response functions or dose-response functions for saturation or lightness. Researchers rarely manipulated the non-hue dimensions as independent factors. Their use also differed by theme. Saturation appeared in 17 emotion-perception families, compared with 6 attention families and 6 environmental families. Studies often bundled saturation and lightness with hue instead of varying them orthogonally (Z. Cao et al., 2024; Guo et al., 2022; Thorstenson et al., 2021).

Named colors do not fully specify a stimulus. The same label can describe different chromaticities and luminances across displays, printers, and rooms. Concrete color values were reported in 264 families, but only 192 named a color space. Luminance control was documented in 186 families and contrast control in 150. Authors noted the confound when color and luminance varied together or luminance was uncontrolled (Ciccone, 2018; Park & Choi, 2025). Seven attention and cognitive-control families compared named colors without coordinates (Bortolotti et al., 2024; Fang et al., 2026; Kuniecki et al., 2015; Yu, 2024; T. Zhu et al., 2025). Their effects cannot be assigned to a particular photometric property. Better controlled studies yield more specific conclusions. The emotional Stroop stimuli of Kang et al. (2022) were spectrophotometrically matched at $100\;{\mathrm{cd/m}}^{2}$, which separates hue from luminance. The photometrically controlled null findings of Schakner (1981) and De Ruyter and Van Dantzig (2019) likewise cannot be dismissed as stimulus failure.

The largest methodological gap appeared in cognitive control. Among 46 families, 18 measured conflict, inhibition, interference, or response selection. Independent variation of color attributes occurred only in general-attention paradigms. These comprised a saturation series in virtual-reality search (A. Jiang et al., 2026), pretrial saturation levels in a dot-probe task (Thoksakis, 2022), and an eye-tracking series that varied hue, luminance, and saturation (W. Jiang & Liu, 2025). Within the core cognitive-control subset, only three metaphorical-congruency families varied brightness independently, and none used photometric calibration (Huang et al., 2018, 2022; Meier et al., 2004). No core family varied saturation independently. Only one family matched luminance photometrically (Kang et al., 2022). Explicit conflict experiments that vary saturation or lightness independently remain the missing intersection. Existing studies address parts of this question, but they estimate different quantities under different control standards.

Incomplete reporting added to the stimulus problems. Only 89 families reported a sample-size rationale, 78 reported blinding, and 180 described missing-data handling. Counterbalancing appeared in 195 families and randomization in 251. Task contrasts also need clearer definitions. A color main effect in an emotional task does not identify an emotional mechanism. A color-by-emotion interaction tests congruency more directly (Frühholz et al., 2011; Park & Choi, 2025). Salience-matched neutral controls can separate emotional meaning from discriminability. Conflict studies should prespecify their interference or inhibition contrast to reduce post hoc selection of significant effects. Authors should report means, variances, analyzed sample sizes, and within-participant correlations for every focal contrast. These statistics would support replication and later quantitative synthesis.

### 4.6. Implications for Quantitative Synthesis

The data structure ruled out meta-analysis even though the corpus contained many numeric results. Removing 44 human-adjudicated source-conflict rows from the 4923 descriptive rows left 4879 non-conflict quantitative candidates. Pooling required effects that could be weighted and clustered without combining unlike estimands or treating dependent results as independent. The evidence met neither requirement. The metric screen retained 569 directly weightable rows from 43 families. Of these, 409 reported an effect with sampling variance and 160 were correlations with derivable Fisher-z variances. Another 1190 rows might have been convertible, but reconstruction would have required design-specific assumptions that were outside this review. More than 3000 rows could not enter a weighting scheme. The homogeneity screen then removed every possible cluster. Only one broad cluster contained direct effects from three families. It mixed correlations with mean differences, combined unrelated outcomes, and contained 230 distinct color contrasts. One family supplied 138 of its 157 direct rows (X. Chen et al., 2019). The final number of homogeneous, poolable clusters was zero.

A broad pooled estimate would have created misleading precision. It would combine estimands with different scales and treat correlated rows from one experiment as independent. Reconstructed effects would also depend on unverifiable stimulus assumptions because of the reporting gaps described in Section 3.10.2. Descriptive synthesis was therefore the appropriate endpoint for this evidence. It preserves meaningful heterogeneity and locates the sparse parts of the literature.

Future meta-analysis will require a narrow protocol amendment. The amendment should define one task family, color contrast, emotional relation, outcome, time point, and effect metric. It should require at least three non-overlapping families with compatible estimates and reconstructable sampling variances. It should also prespecify a dependence model for multiple effects within a family. Facial-color expression judgments and brightness-valence congruency are the most mature candidates for targeted re-extraction. Even these subgroups need colorimetric reporting that can support pooling.

### 4.7. Strengths and Limitations

The review’s unit structure was a principal strength. We separated reports, study families, experiments, samples, contrasts, outcomes, and time windows. This prevented overlapping reports or samples from being counted as independent evidence, while distinct samples could form different families. It also kept variation in row granularity out of prevalence estimates. We validated all final thematic denominators against the registry of 294 families. The handling of contradictory information was another strength. We kept 44 source-internal conflict rows in the descriptive evidence but excluded them from quantitative candidacy. Documented inconsistencies could therefore not enter effect reconstruction without notice (Duan et al., 2018b; Ioan et al., 2007; G. Xia et al., 2021). Three rounds of citation searching broadened coverage beyond the database search and changed the evidence distribution. They recovered older studies, applied work, and replications, including the oldest family (Pressey, 1921) and an important failed replication (Steele, 2014). The completed pre-submission update provided a dated check against recent indexing changes.

Coverage remained incomplete despite the 17 August 2026 update. The evidence set required accessible English-language full texts, which under-represented non-English and inaccessible studies. Neither the initial database search nor the update included direct PsycINFO or Web of Science coverage. Of the 294 study families, 140 were identified only through citation searching. This reliance may have favored studies connected to the selected seed reports. Eligible studies outside those citation networks may have been missed. In the initial database branch, 213 of the 1053 reports sought for retrieval were not retrieved. The final backward-citation search (CCA-04) also retained 1803 records without abstracts under the documented stopping rule. Those records did not receive eligibility assessment. They form an explicit and bounded gap in the final backward-citation search.

Limitations in primary reports carried into the synthesis. Only 192 families identified a color space, 186 documented luminance control, and 150 documented contrast control. Calibration and ambient conditions were rarely reported. These omissions restrict attribution of a hue effect to a photometric property. Task heterogeneity was also substantial. The residual “other” task label contained 61 of 64 environmental families and 35 of 36 physiology families. Comparisons across families therefore often involved different estimands. Multiple results from the same participants could be statistically dependent. By design, we did not fit a statistical model to these dependencies.

The synthesis also depended on two simplifying devices. We assigned every family to one of six themes, although many families crossed tasks, roles, outcomes, and mechanisms. The assigned theme indicates the family’s main contribution and does not imply that secondary processes were absent. Mechanism labels came from report language and reviewer mapping. We did not conduct a formal risk-of-bias assessment or assess certainty of evidence. Frequencies of reporting fields describe reproducibility gaps and do not rank causal validity. Effectiveness claims would require a narrower systematic review with critical appraisal. The present review therefore maps reported findings without adjudicating their overall effectiveness.

Ecological validity was not a prespecified charting construct. The extraction form recorded populations, study locations, tasks, color roles, and outcomes. It did not place naturalism, field implementation, exposure duration, or transfer to downstream functional outcomes on a common scale. The practical interpretation therefore uses applied settings and outcomes already present in the charted data. It cannot provide a prevalence estimate of real-world effectiveness.

### 4.8. Implications for Future Research

Future emotion and cognitive-control experiments should specify the physical color manipulation. Studies should vary hue, saturation, and lightness independently instead of relying on named-color comparisons. Authors should report the color space and full coordinates for every stimulus. They should control and report luminance and local contrast, identify the display or light source, name the calibration instrument, and document ambient illumination. Screening for color-vision deficiencies is also needed. At least one family already meets each requirement (De Ruyter & Van Dantzig, 2019; Kang et al., 2022; Schakner, 1981; T. Xia et al., 2018). These practices are therefore feasible, although they are not yet conventional.

The role of color should also be defined before analysis. Color can be a target, cue, distractor, response feature, background, or illumination condition. Role was associated with the kind of effect observed in attention and cognitive control (Section 3.4.3). A color-by-emotion interaction can test congruency, while salience-matched neutral controls can test whether discriminability explains the result. Reaction time and accuracy should be reported together because several conflict families showed speed–accuracy trade-offs (Park & Choi, 2025; Xu & Zhang, 2019). Eye tracking and ERP can locate the processing stage at which an effect occurs. Conflict paradigms should prespecify the interference or inhibition estimand. A color main effect in an emotional task does not establish an emotional mechanism. Null results also need reconstructable statistics so that they can constrain the evidence (Michaelis, 2013; Schakner, 1981; Steele, 2014).

Applied studies need a direct transfer test. Researchers should prespecify a functional endpoint and measure it alongside proximal measures such as reaction time, gaze, or physiology. Tests should use the target population and intended device. They should also reproduce the intended illumination, exposure duration, and task demands. Claims about sustained benefit require repeated exposure or follow-up.

The candidate accounts produce competing hypotheses that can be preregistered. Under a perceptual-salience account, effects should track measured physical discriminability. Tests should match or model luminance, chromatic contrast, and other low-level differences. Under an arousal account, state changes should carry over to performance. They should covary with autonomic, pupillary, or EEG indices and persist after salience matching. Affective congruency predicts a color × emotion interaction, with matched relations facilitating and mismatched relations impairing performance. Attentional capture predicts that emotionally or physically diagnostic colors draw or hold selection under competition, cue-validity, or distractor-interference contrasts. Early ERP components and gaze can test this prediction. Conflict modulation predicts a change in the prespecified interference or inhibition effect. Overall performance does not test this account. Response compatibility predicts that a color’s connotation primes compatible or incompatible response channels. This account requires manipulation of stimulus–response assignments. Perceptual contrasts do not test it.

More than one account may operate in the same experiment. Most published designs could not separate them. Designs that independently manipulate the defining contrasts for two accounts, such as congruency and salience or capture and conflict, can distinguish the explanations more clearly.

Replication should carry more weight. The difference between the original finding (Mehta & Zhu, 2009) and its close replication (Steele, 2014) shows that an influential color-cognition claim may fail under a similar test. The mixture of detected and null results within families also means that another isolated positive study will not resolve the competing accounts. Replications should preserve stimulus coordinates, luminance, task timing, and analytic contrasts. They should report null results with reconstructable statistics. Across this review, reported patterns differed across manipulated properties, color roles, emotional relations, comparators, and response levels. Better controlled, preregistered experiments can test which of these conditions reflects a mechanism.

## 5. Conclusions

This scoping review mapped reported effects of experimental variation in color on emotional attention, cognitive control, and emotional information processing. The 294 study families yielded 4923 descriptive rows. Reported patterns varied across color properties, task roles, emotional relations, comparators, and outcome levels. These levels included abstract association, experienced emotion, attentional orienting and disengagement, perceptual salience, conflict and inhibition, response selection, memory, self-report, and neural or physiological response. This descriptive mapping does not establish a general performance benefit or impairment for any named color across tasks. Effects on one outcome often did not appear at other outcome levels within the same family. The observed heterogeneity warrants task-specific questions for future synthesis.

Rows with within-source conflicts informed only the descriptive synthesis, and rejected extractions were excluded. Effects without reconstructable sampling variance were ineligible for pooling. These restrictions limit synthesis but do not, by themselves, determine the causal status of the original designs. Future studies should prespecify the color property and task role, match luminance and contrast, and report complete quantitative results. These choices will allow researchers to distinguish candidate mechanisms. Applied use also requires validation in the target population, device, illumination, and context. Changes in gaze or physiology alone do not establish a real-world benefit without a corresponding functional outcome.

## Figures and Tables

**Figure 1:**
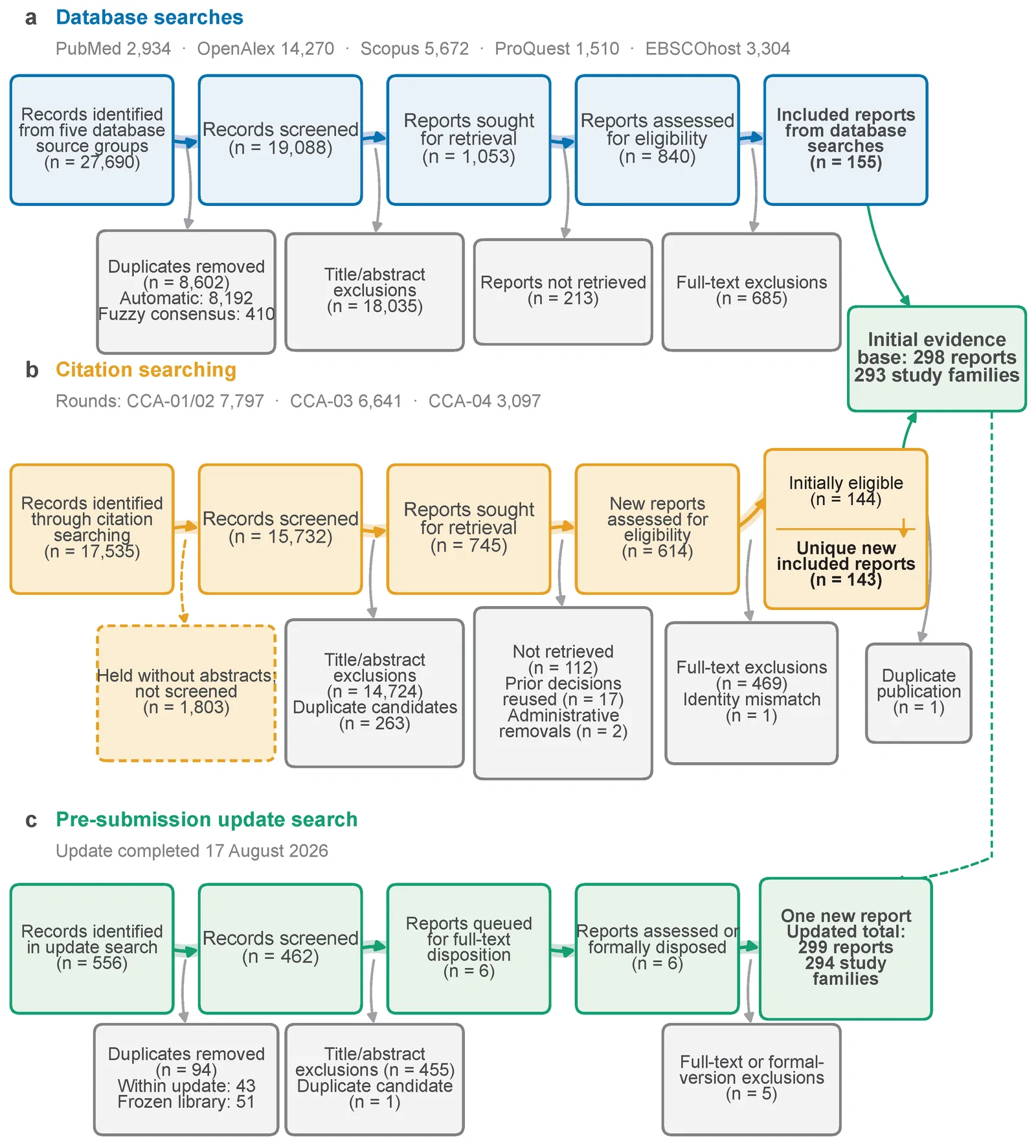
Preferred Reporting Items for Systematic Reviews and Meta-Analyses extension for Scoping Reviews (PRISMA-ScR) study-selection flow for the initial database searches, citation searching, and the 17 August 2026 pre-submission update. Blue, orange, and green connectors indicate the database, citation-searching, and update or inclusion pathways, respectively; gray connectors indicate removals or exclusions. Connector widths do not encode counts. The frozen initial evidence base contained 298 reports grouped into 293 study families. The update added one report and one family, yielding 299 reports grouped into 294 families. Table S7 lists the primary reasons for full-text exclusion. CCA denotes citation-chasing amendment.

**Figure 2:**
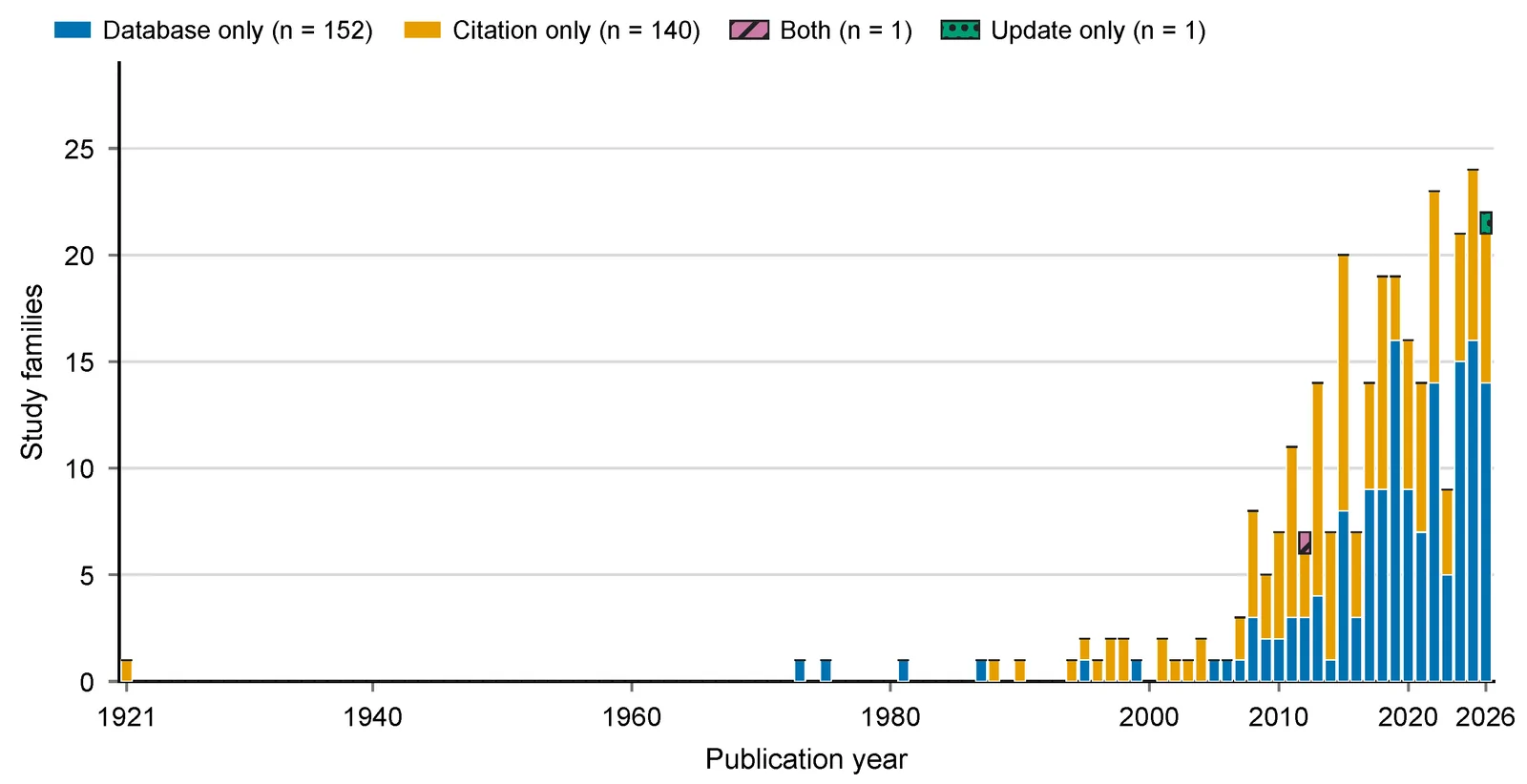
Publication years for the 294 study families. Each family is counted once in a mutually exclusive search-route category: 152 database-only, 140 citation-searching-only, one represented in both initial routes, and one added only by the pre-submission update. Publication year was assigned from the analysis-preferred report for each family.

**Figure 3:**
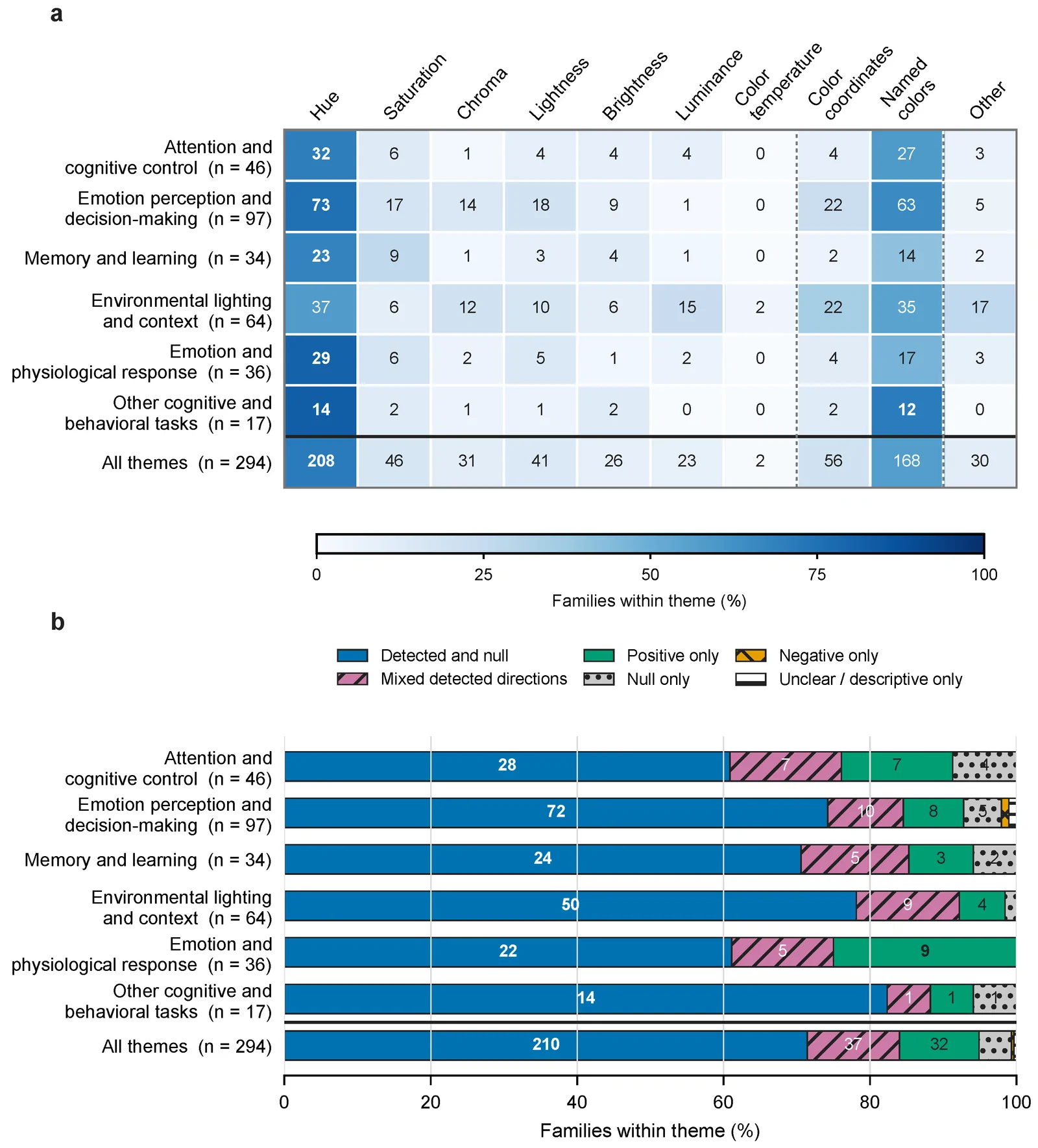
Family-level evidence across thematic domains. Panel (**a**) maps unique study families to each extracted color category by primary theme. Cell color gives the percentage of families within each theme, and labels give family counts. Color-category labels are non-exclusive. Panel (**b**) shows mutually exclusive family-level result patterns after removal of 44 source-conflict rows. Positive and negative retain the recorded comparison or interpretation and do not uniformly indicate benefit or harm.

**Figure 4:**
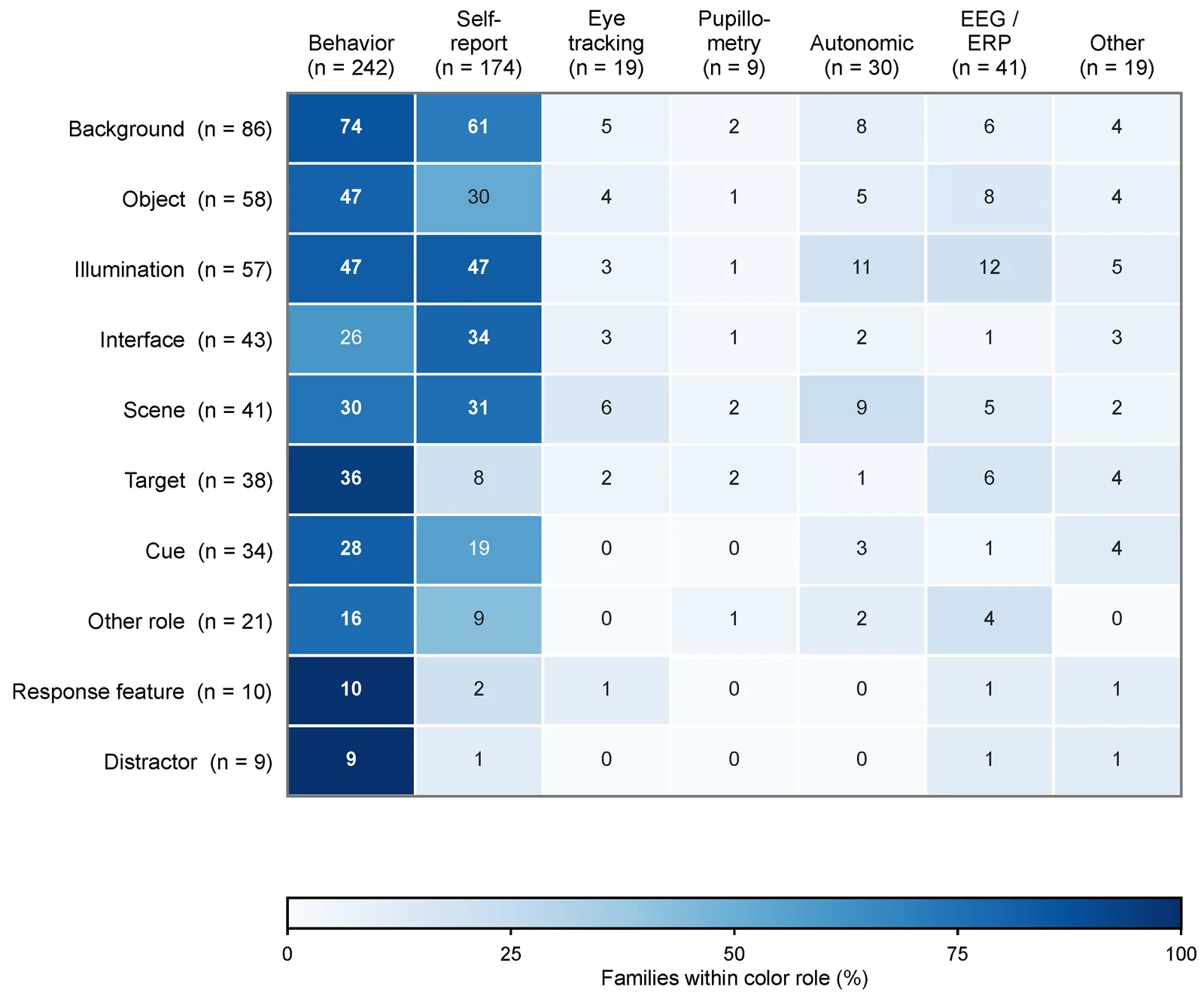
Result-linked measurement coverage by color role. Each cell gives the number of unique study families in which the color role and measurement modality occurred in the same extracted result row. Cell color gives the percentage of families carrying the row’s color-role label, and the number gives the family count after family-level de-duplication. Electroencephalography (EEG) and event-related potential (ERP) measures were combined for display. Color-role and modality labels were non-exclusive, so row totals, column totals, and cell counts are not additive. All 4923 descriptive result rows were considered; removing the 44 source-conflict rows left the displayed pairs unchanged.

**Figure 5:**
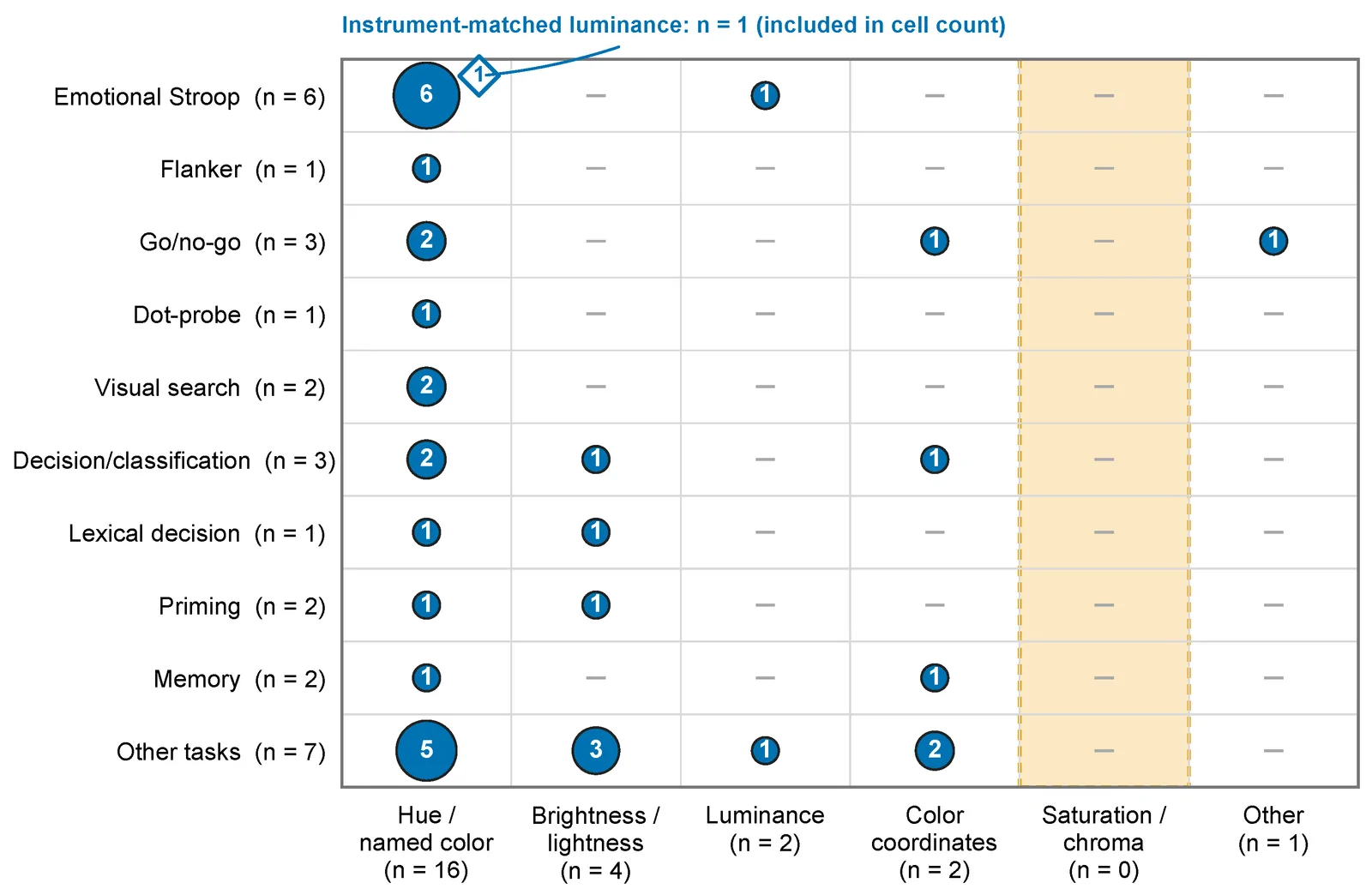
Evidence coverage in the core cognitive-control subset (*N* = 18 unique study families). Bubble area and center number show unique families in each task-by-attribute combination. Task and color-attribute labels are non-exclusive, so row totals, column totals, and cell counts overlap and must not be added. The outlined diamond marks one family (*n* = 1) within the Emotional Stroop-by-hue/named-color cell; it used a PR-650 spectrophotometer to match stimulus luminance at $100\;{\mathrm{cd/m}}^{2}$. This family is included in the cell count. No family (*n* = 0) was coded for saturation or chroma. The strict instrument-matching definition excludes studies that only fixed ambient illumination.

**Figure 6:**
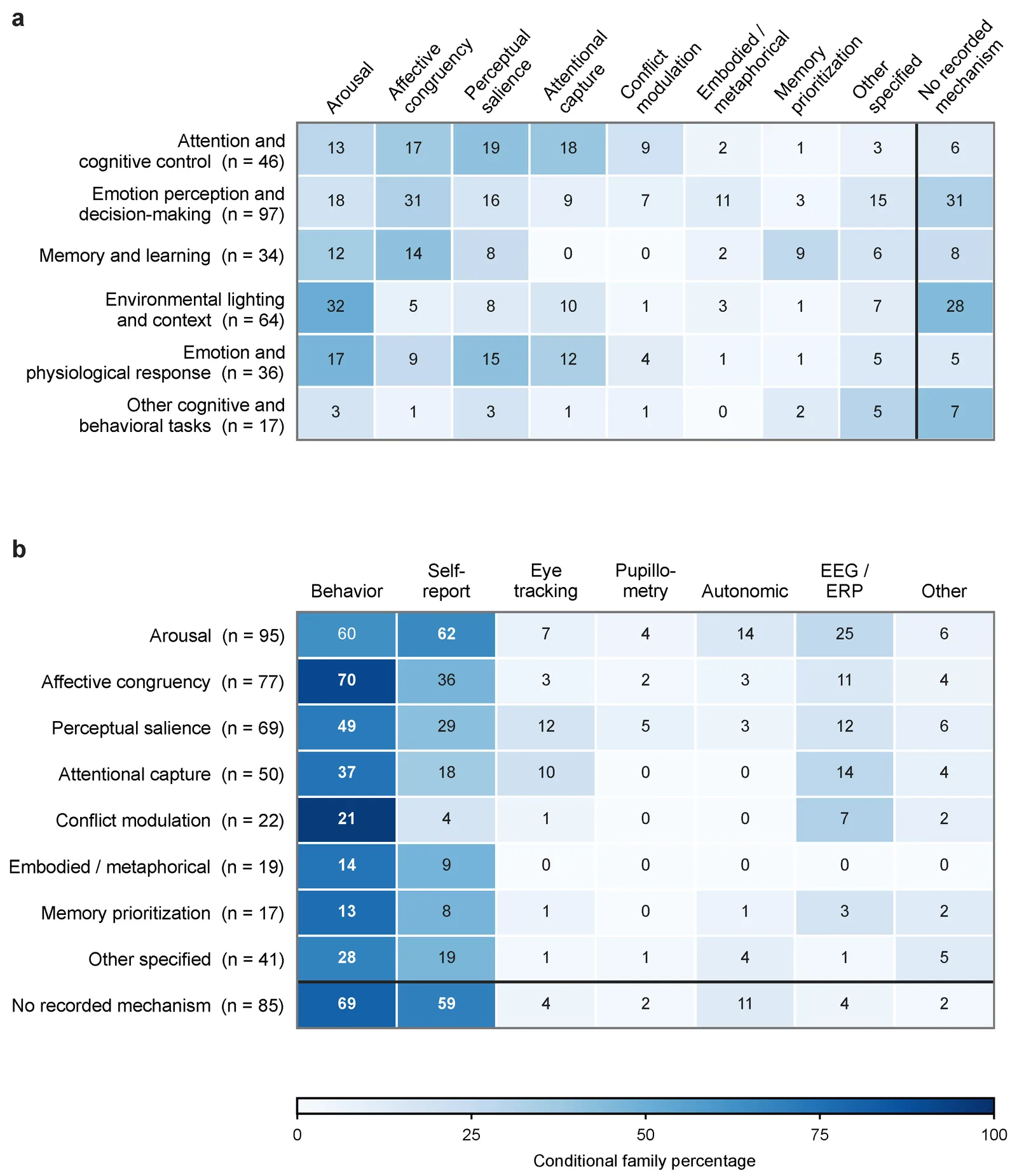
Distribution of candidate mechanism labels across thematic domains and measurement modalities. Panel (**a**) maps unique study families to each candidate mechanism by primary theme. Cell color gives the percentage of families within each theme, and labels give family counts. Panel (**b**) shows measurement modalities recorded in result rows carrying each candidate mechanism. Cell color gives the percentage of families mapped to that mechanism, and labels give unique family counts after within-row mechanism–modality pairing and family-level de-duplication. Electroencephalography (EEG) and event-related potential (ERP) measures were combined for display. Mechanism and modality labels were non-exclusive. Co-occurrence does not demonstrate mediation or a causal pathway.

**Figure 7:**
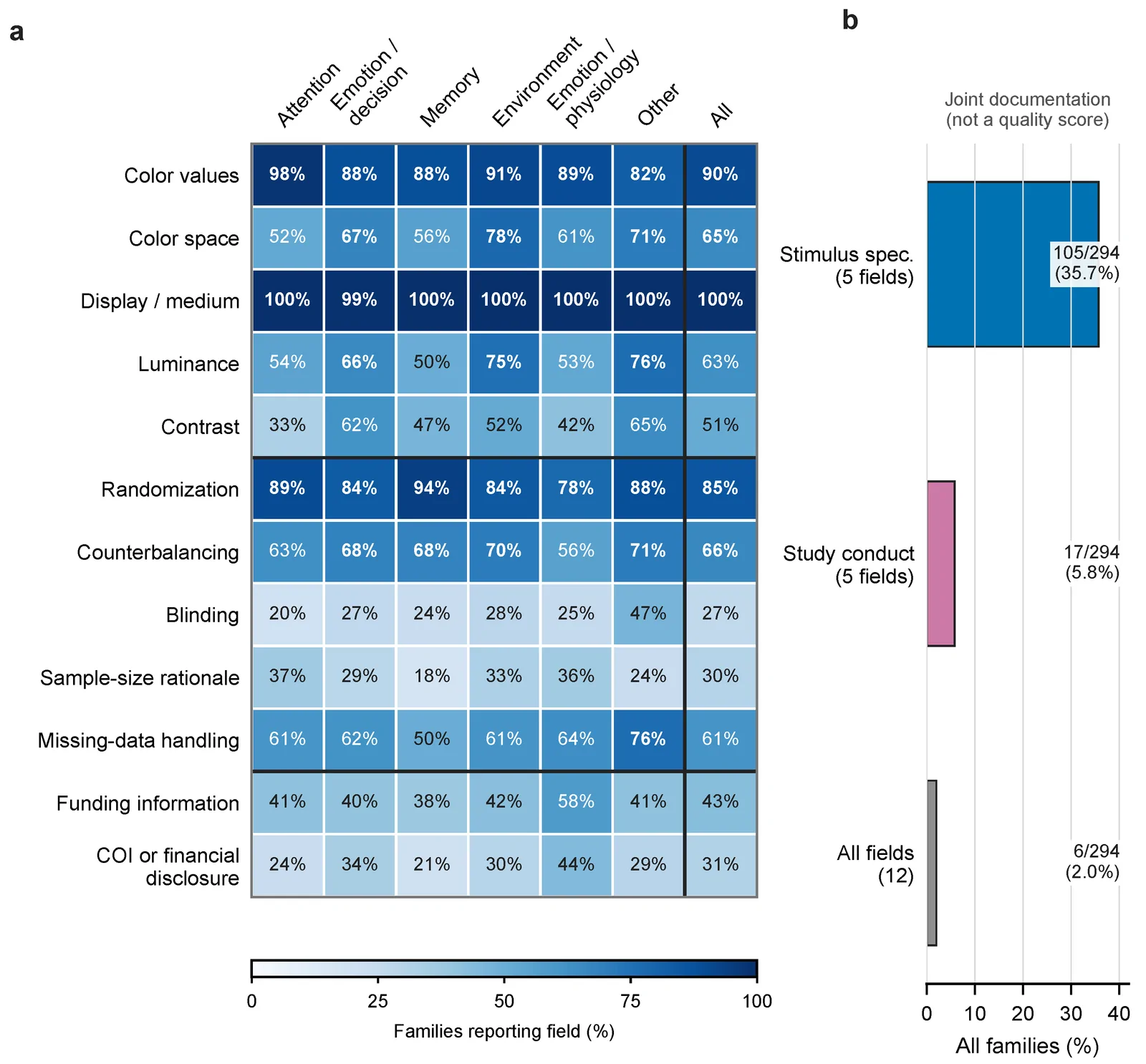
Family-level reporting completeness by thematic domain. Panel (**a**) counts a field as reported when at least one result row in the family contains usable information; cells show the percentage of families within each theme. Panel (**b**) shows joint documentation across all 294 families: 105 reported all five stimulus-specification fields, 17 reported all five study-conduct fields, and 6 reported all 12 fields. These values describe documentation and are not a methodological quality score. Abbreviations: COI, conflict of interest; spec., specification.

**Figure 8:**
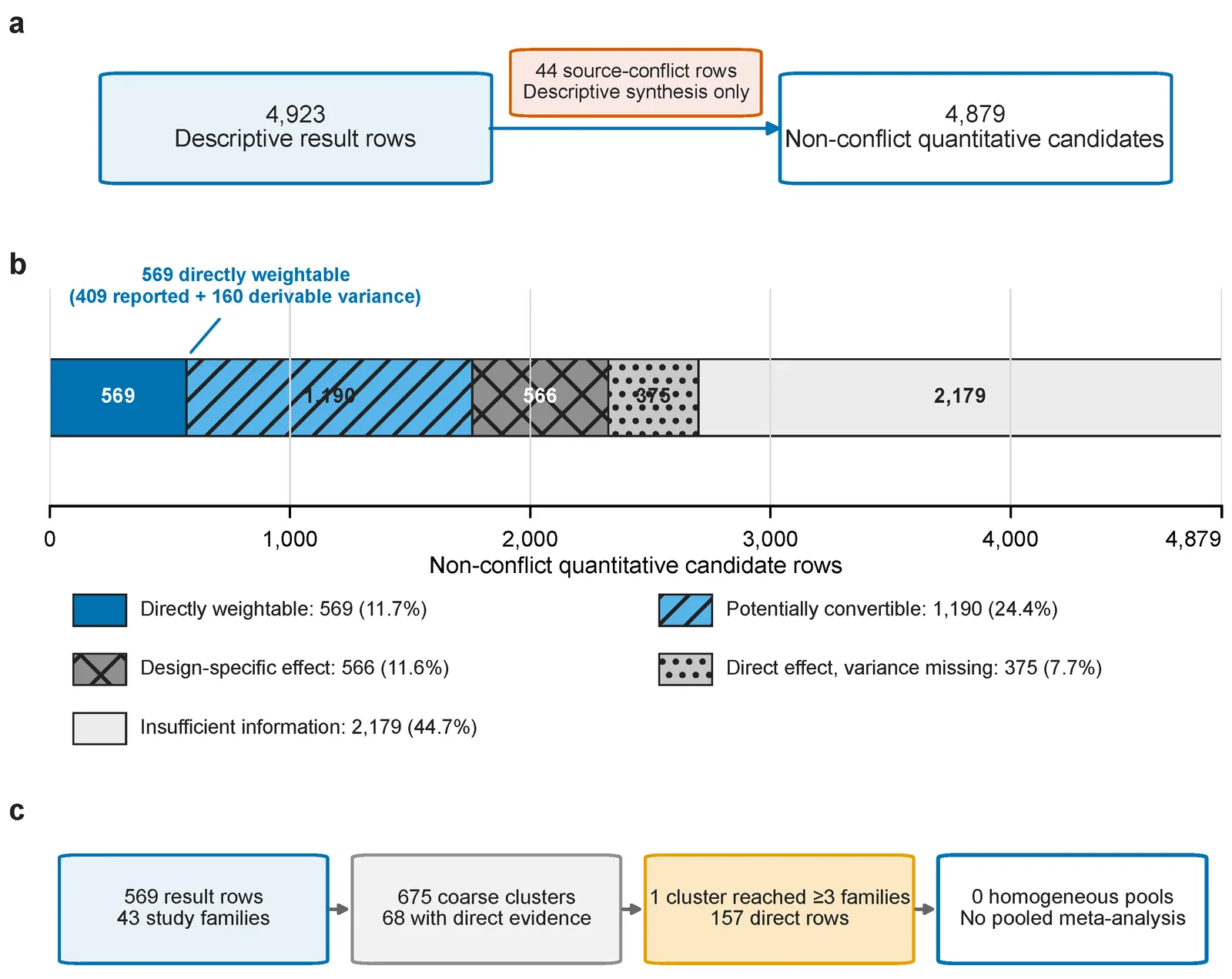
Gates for quantitative-synthesis readiness. Panel (**a**) shows that 44 of 4923 descriptive rows were retained for description only because of source conflict. Panel (**b**) partitions the remaining 4879 candidates into mutually exclusive readiness categories; the 569 directly weightable rows comprise 409 rows with reported sampling variance and 160 correlations with derivable Fisher-*z* variance. Panel (**c**) shows that the 569 rows came from 43 families, while all 4879 candidates formed 675 coarse clusters. Direct-effect families per cluster were distributed as 0 in 607 clusters, 1 in 63, 2 in 4, and 3 in 1. The sole threshold-reaching cluster contained 157 direct rows but failed the contrast, estimand, metric, and dependence audit, leaving no homogeneous pool. Units change across gates; box areas and arrow widths are not proportional to counts.

**Table 1:** Study families and descriptive result rows in the six mutually exclusive themes.

Theme	Study Families	Descriptive Result Rows
Attention and cognitive control	46	479
Emotion perception and decision-making	97	1620
Memory and learning	34	406
Environmental lighting and context	64	1760
Emotion and physiological response	36	372
Other cognitive and behavioral tasks	17	286
Total	294	4923

**Table 2:** Population, setting, and design characteristics recorded for the 294 study families.

Characteristic	Recorded Category	Families, *n* (%)
Reported sample mean age	At least one recorded mean below 18 years	13 (4.4)
	At least one recorded mean of 18 to 64 years	180 (61.2)
	At least one recorded mean of 65 years or older	5 (1.7)
	Usable mean age unavailable	100 (34.0)
Clinical status	Explicitly healthy or nonclinical	186 (63.3)
	Clinical or health condition recorded	16 (5.4)
	Other description; clinical status not established	68 (23.1)
	Clinical status unavailable	24 (8.2)
Country or region recorded	United States	67 (22.8)
	China	57 (19.4)
	Germany	32 (10.9)
	Japan	22 (7.5)
	United Kingdom	15 (5.1)
	France	10 (3.4)
	Other named countries or regions	96 (32.7)
	Country or region unavailable	12 (4.1)
Recorded setting	Laboratory or controlled room	218 (74.1)
	Online	31 (10.5)
	School or classroom	30 (10.2)
	Clinical or home setting	6 (2.0)
	Other field setting	5 (1.7)
	Other or insufficiently specified setting	22 (7.5)
	Setting unavailable	6 (2.0)
Design description	Within-participant or repeated-measures	127 (43.2)
	Between-participant or parallel	83 (28.2)
	Mixed within- and between-participant	45 (15.3)
	Several design descriptions within the family	19 (6.5)
	Single-case or reversal design	3 (1.0)
	Other design or allocation unclear	17 (5.8)
Analyzed sample size	One charted sample with an explicit analyzed N	149 (50.7)
	Multiple samples, varying N, or textual N	135 (45.9)
	Analyzed N unavailable	10 (3.4)

All percentages use 294 families. Mean-age, country, and setting categories may overlap. Age bands refer to reported sample means, not the ages of every participant. Country names describe the charted locations; institutional affiliation alone was not used to fill missing locations. Clinical groups may include comparison participants. Generic student or community descriptions were not classified as healthy. The analyzed N statistic is restricted to families with one charted experiment-sample key and one unambiguous analyzed participant count. Multiple-sample totals were not calculated. Raw descriptions and source locations appear in Table S2. Among 149 qualifying families, the median analyzed N was 42 (interquartile range of 24 to 100; range of 1 to 1500).

**Table 3:** Result-row lineage and quantitative-synthesis gates.

Stage	Rows or Families	Analytical Use
Raw extraction across the frozen initial sources and update	5088 rows	Audit source
Superseded duplicate or shared-sample results	112 rows	Removed from canonical analysis
Canonical all-status ledger	4976 rows	Includes rejected rows
Rejected extraction rows	53 rows	Excluded from synthesis
Descriptive synthesis	4923 rows	Evidence mapping and narrative synthesis
Source-conflict results	44 rows	Description only
Non-conflict quantitative candidates	4879 rows	Assessed for metric and homogeneity gates
Directly weighted effects	569 rows from 43 families	Entered cluster audit
Potentially convertible statistics	1190 rows	Require design-specific reconstruction
Homogeneous poolable clusters	0	No pooled meta-analysis

## Data Availability

The original contributions presented in this study are included in the article and Supplementary Materials. Versioned charting data, the study-family crosswalk, analysis code, validation files, and reporting checklists are publicly available in the OSF project at https://osf.io/ku458/, accessed on 22 September 2026.

## Supplementary Materials

The following supporting information can be downloaded at https://www.mdpi.com/article/10.3390/bs16101769/s1, Table S1: Included-report inventory; Table S2: Study-family characteristics; Table S3: Canonical result-level data; Table S4: Reporting completeness; Table S5: Meta-analysis feasibility; Table S6: Full search strategies actually executed (v2.1); Table S7: Primary full-text exclusion reasons by search branch (v2.1); Supplementary Methods S8: Interpretation of result codes and construction of family summaries. Copyrighted full-text reports are not redistributed.

## Abbreviations

CCA	Citation-chasing amendment
CI	Confidence interval
EEG	Electroencephalography
ERP	Event-related potential
JBI	Formerly the Joanna Briggs Institute
PRISMA-ScR	Preferred Reporting Items for Systematic Reviews and Meta-Analyses extension for Scoping Reviews
PRISMA-S	PRISMA literature-search reporting extension

## Appendix A. Included Sources of Evidence

All 299 included publication reports are cited in the groups below. Table S1 links each citation to its report identifier, study-family assignment, source type, inclusion status, and verified bibliographic metadata.

1. (Afifi et al., 2024; Barba et al., 2018; A. N. Chen et al., 2018; de Vries et al., 2018; Elliot et al., 2007, 2011; A. Ludlow et al., 2008; A. K. Ludlow et al., 2013; Richards & Fink, 2017; Santos et al., 2010; Segal et al., 2016; Soldat et al., 1997)
2. (AL-Ayash et al., 2016; Alkozei et al., 2016; Ávila-Muñoz et al., 2026; Batistatou et al., 2022; Cooper et al., 1994; Hulshof, 2013; A. Jiang et al., 2023; Nissen, 2020a, 2020b; Nissen & Riedl, 2021; Nissen et al., 2024; Ruba et al., 2021)
3. (Bekhtereva & Müller, 2017; Bertrams et al., 2015; Bharti et al., 2026; Bilucaglia et al., 2019; Blase et al., 2024; Blazhenkova et al., 2022; Bortolotti et al., 2024; De Ruyter & Van Dantzig, 2019; Krenn, 2014; Meier et al., 2015; Vyas et al., 2025; Zhou et al., 2025)
4. (Burattini et al., 2019; Byrne et al., 2019; Cano et al., 2009; Y. Cao et al., 2021; Chai et al., 2019; Chamaret, 2012; Chan & Park, 2015; C. Chen et al., 2025; L. Chen et al., 2026; Meier et al., 2004; Sezer & Olguntürk, 2026; Williams et al., 2011)
5. (Barkmann et al., 2011; Braun et al., 1995; J. Chen & Song, 2025; S. Chen et al., 2023; J. Choi et al., 2020; Chung, 2015; Cipollone, 2024; Conway et al., 2010; Kawai et al., 2020; Kuhbandner & Pekrun, 2013; Thorstenson, 2014; Tuffentsamer, 2018)
6. (De Lucia, 1975; Désiron & Schneider, 2024; Ding et al., 2022, 2023; Fang et al., 2026; Houtman & Notebaert, 2013; Lakens, 2010; Lakens et al., 2013; Larsson & von Stumm, 2015; Öztürk et al., 2011; Pak et al., 2026; Spiridon, 2017)
7. (Farley & Grant, 1973; Forrester et al., 2025; Fotios et al., 2017; Frühholz, 2008; Frühholz et al., 2009, 2011; Gil & Le Bigot, 2014; Goodhew & Kidd, 2020; Gorn et al., 2004; G. Li & Kawabata, 2021; Vandewalle et al., 2011, 2010)
8. (Goolsby et al., 2009; Guo et al., 2022; Hasegawa et al., 2025; Hawes et al., 2012; Hine & White, 2021; Hosseini & Ghabanchi, 2022; Hsieh et al., 2024; K. Hu, 2019; K. Hu et al., 2020a; Huang et al., 2018; Nguyen et al., 2023; G. Xia et al., 2021)
9. (Bedwell et al., 2011; Hui et al., 2024; Ikeda, 2020; Iwakiri et al., 1999; Jacobson et al., 2024; A. Jiang et al., 2026; Knez & Niedenthal, 2008; Moser et al., 2015; J.-E. Pelet et al., 2013; J.-E. Pelet & Papadopoulou, 2010; J.-É. Pelet & Papadopoulou, 2012; Plass et al., 2014)
10. (Domínguez-Borràs et al., 2013, 2020; W. Jiang & Liu, 2025; Jung et al., 2011; Kang et al., 2022; Kanojiya & Zinzuvadiya, 2025; Kaspar et al., 2015; Kato et al., 2022; Kawasaki & Yamaguchi, 2012; Quiles-Rodríguez & Palau, 2024; Shi et al., 2015; Venni & Bétrancourt, 2020)
11. (Ash et al., 2013; K. Choi & Suk, 2020; K. Hu et al., 2020b; Ishikawa et al., 2018; Killelea, 1987; Knez, 1995; Kocaoğlu, 2015; Kuniecki et al., 2015; Ladeira et al., 2025; Lan et al., 2021; Lang et al., 2025; Steele, 2014)
12. (L. Hu et al., 2024; Langeslag et al., 2011; Lasauskaite et al., 2023; C. Li et al., 2024; X. Li et al., 2025; Y. Li, Fang, Qiu, & Wang, 2024; Y. Li, Fang, Qiu, Yu, et al., 2024; Liao et al., 2022; Rook, 2014; Viegas et al., 2024; L. Wang et al., 2019; L. Zhao et al., 2024)
13. (Chittaro, 2016; Lipnitsky & Polyak, 2026; B. Liu et al., 2026; C. Liu et al., 2018; Llinares et al., 2021; N. Lucas & Vuilleumier, 2008; R. Lucas, 2022; Luo et al., 2019; Ma et al., 2020; Mammarella et al., 2016; Silic & Cyr, 2016; Whitaker et al., 2015)
14. (Du et al., 2025; Hochel et al., 2009; Maier et al., 2008; Martin-Kratzer, 2005; McDonnell et al., 2025; McMenamin et al., 2013; Michaelis, 2013; Okubo & Ishikawa, 2011; Silic et al., 2017; Stanković et al., 2024; Vandermause et al., 2022; van Hagen et al., 2009)
15. (Minami et al., 2018; M Nael, 2019; Moran & Moser, 2015; P. Müller & Wentura, 2023; S. Müller et al., 2016; Nakajima et al., 2015, 2017; Nakakoga, 2021; Nambiar, 2026; Stone, 2001; Stone, 2003; M. Xia et al., 2026)
16. (Ciccone, 2018; Hagemann et al., 2008; Kwallek & Lewis, 1990; Kwallek et al., 1996, 1988, 1997; Michels et al., 2022; Nie et al., 2026; Pontes & Hoegg, 2019; Pontes & Williams, 2020; Shishegar & Boubekri, 2022; Stone & English, 1998)
17. (Ab Jalil et al., 2021; Bengart & Vogt, 2023; Kombeiz, 2017; Kombeiz & Steidle, 2017; Olsen, 2010; Park & Choi, 2025; Peromaa & Olkkonen, 2019; Perry & Henik, 2013; Pilarczyk et al., 2020; Prasetya, 2016; Puechbroussou & Rusinek, 2021; Wu et al., 2022)
18. (Bagchi & Cheema, 2012; Westerman et al., 2011; Küller et al., 2009; Laureanti et al., 2021; Lunardo et al., 2021; Mehta & Zhu, 2009; Qu & Kato, 2026; Ramos-Nuñez et al., 2018; Raudonis et al., 2017; Rhodes, 2019; Ru et al., 2019; Sinclair et al., 1998)
19. (Cha et al., 2020; Gil & Le Bigot, 2015, 2017; Kübel et al., 2021; Miran et al., 2017; Schakner, 1981; Schindler et al., 2019; SEJIMA et al., 2016; Shehab & Durmus, 2026; Shibusawa et al., 2025; Smajic et al., 2014; Son & Yeom, 2026)
20. (Heidig et al., 2015; Hygge & Knez, 2001; Ioan et al., 2007; Lichtenfeld et al., 2012; Pressey, 1921; Simoës-Perlant et al., 2026; Sivananthan et al., 2024; L. Song et al., 2024; S. Song & Yamada, 2019; Stefanics et al., 2018, 2019; Young et al., 2013)
21. (Gnambs, Appel, & Oeberst, 2015; Hatta et al., 2002; Lemos et al., 2019; T. Li et al., 2023; Mao et al., 2018; Peromaa et al., 2025; Shavit et al., 2013; Sivananthan et al., 2021; Takei & Imaizumi, 2022; Thoksakis, 2022; Thorstenson et al., 2021; Yamada et al., 2025)
22. (Buechner et al., 2014; Chulvi et al., 2022; Gnambs et al., 2010; Gnambs, Appel, & Kaspar, 2015; T. Jiang et al., 2022; T. Li et al., 2026; Liew et al., 2022; Um et al., 2012; Ursuţiu et al., 2017; Uzun, 2017; Uzun & Yıldırım, 2018; T. Zhu et al., 2025)
23. (X. Chen et al., 2019; Dou et al., 2026; Tantanatewin & Inkarojrit, 2018; von Castell et al., 2018; F. Wang et al., 2025; X. Wang et al., 2022, 2026; Weigand et al., 2021; Wen et al., 2025; West et al., 2010; T. Xia et al., 2018; Xu & Zhang, 2019)
24. (Duan, 2017; Duan et al., 2018a, 2018b; Huang et al., 2022; Le et al., 2018; X. Li et al., 2026; Peng et al., 2026; Y. Wang et al., 2021; Yasuda, 2007; Yu, 2024; Y. Zhao et al., 2025; Zuo & Kawabata, 2024)
25. (Z. Cao et al., 2024; Y. Chen et al., 2026; Cömert & Samur, 2025; Sakuta & Gyoba, 2006; Shao et al., 2024, 2025; Zak, 2015; Zak et al., 2015; Zhang et al., 2023; Y. Zhu et al., 2017)
26. (Hao & Wang, 2026)
