# Supplementary material for: Beyond Associations Between Color and Emotion: A Scoping Review of Task-Based Evidence on Color Properties, Emotional Attention, and Cognitive Control
Source: Behav Sci (Basel). 2026 Sep 28;16(10):1769. doi: 10.3390/bs16101769 (PMC13701247; doi:10.3390/bs16101769)
Supplement: Supplementary file 1 [file behavsci-16-01769-s001.zip › Supplementary_Methods_S8.pdf]

Supplementary Methods S8. Interpretation of result codes and construction of family summaries

S8.1. The result being coded

Table S3 contains 4,923 result rows from 294 study families. A row identifies one combination of study family, report, experiment, sample, contrast, outcome, and time window. The `seven_field_key` preserves this combination. The examples below use this key so that readers can locate the exact records in Table S3.

The direction field describes the result recorded for that contrast. Its interpretation depends on the `contrast_definition`, `reference_condition`, `outcome_orientation`, and `curated_interpretation` fields. The associated statistic, model, uncertainty, and source location remain separate fields. A test statistic stored in `effect_estimate`, such as a t or F value, must be read with `effect_metric`; it is not automatically a standardized effect estimate.

S8.2. Meaning of the five codes

The charting vocabulary contains `positive`, `negative`, `no_detectable_difference`, `mixed`, and `unclear`. These labels retain the interpretation recorded during source-level extraction. They were not generated by applying one numerical sign convention across all outcomes.

Code	Meaning in the charted data
`positive`	The row records a positive direction under its stated comparison or interpretation. This can describe a numerical increase or a task-oriented result, such as facilitation.
`negative`	The row records a negative direction under its stated comparison or interpretation. This can describe a numerical decrease or the reverse of the specified relation.
`no_detectable_difference`	The source reports no detected difference or association for the recorded comparison. This includes results that did not meet the source's stated inferential threshold.
`mixed`	The retained result has conditional or multiple directions, or an omnibus pattern that is not represented by one positive or negative contrast. The interpretation field specifies the pattern.
`unclear`	The available result does not support a definite directional interpretation. A reported statistical test can still accompany this code.

The extraction retained both numerical and task-oriented descriptions. For example, faster responses received a positive code when charted as facilitation in R008347. A reduction in response latency received a negative code in CC20260804_03698. Positive and negative therefore do not provide a common measure of benefit, harm, or numerical increase across the corpus. The comparison and source interpretation determine what each label means. No code gives the magnitude, precision, or certainty of an effect.

`No_detectable_difference` does not mean that the effect is zero. A narrative statement of a difference can retain a directional code when exact statistics are not reported. Conversely, a statistically significant omnibus test can receive `unclear` when its direction cannot be resolved. Direction coding and statistical detection are related information, but they are not interchangeable fields.

S8.3. Reported inference and multiple comparisons

The summary script uses the stored direction labels. It does not recalculate them from p values using a common alpha threshold. Table S3 records source-specific inferential information in `p_value`, `confidence_interval`, `ci_includes_null`, `test_statistic`, `analysis_model`, and `multiplicity_handling`. A missing p value or adjustment remains missing; it is not converted into a new numerical value.

The multiple-comparison field can describe more than one procedure within a report. Its application must be matched to the relevant test and source location. In CC20260811_05757, 48 correlations involving the Phenomenology of Consciousness Inventory used a Bonferroni threshold of .001. Four directional color

comparisons used .0125. The same report also contained other follow-up tests. Applying the smallest threshold to every row in that report would misrepresent its analyses.

One correlation in that report had r = -.367 and p = .030. Table S3 codes it as `no_detectable_difference` under the .001 threshold for that set of correlations. The red versus green duration comparison had p = .011 and was coded `positive` under the .0125 threshold. These examples show why p < .05 alone is insufficient to reproduce the source-level coding.

Some rows record source conclusions without exact statistics. R015112 describes lower frustration under a yellow background, with p value and test statistic recorded as not reported. Its negative code retains that narrative direction. It does not establish that the review independently confirmed statistical significance. The source's reporting limits remain part of the evidence record.

S8.4. Main effects, interactions, and several color levels

A color main effect concerns the comparison across the other factor levels in its model. A color-by-emotion interaction concerns variation in a color effect across emotional conditions. A color comparison within one emotional condition is a simple contrast. These results answer different questions and occupy separate rows when independently interpretable results are available.

The `color_effect_scope` field distinguishes `color_main_effect_in_emotional_task`, `color_by_emotion_interaction`, `color_contrast_within_emotional_condition`, and `unclear`. The contrast and model identify the estimand. A main effect in an emotional task does not by itself establish emotion-specific modulation. A difference within one emotional condition also does not establish an interaction without a comparison across conditions.

An omnibus F test can show that several color conditions differ without specifying a signed pairwise effect. Its non-negative F value cannot supply a positive direction. The charted code instead follows the accompanying comparison and result description. A conditional pattern can receive `mixed`; an unresolved direction can receive `unclear`. Separately reported follow-up contrasts retain their own rows. The review does not infer unreported pairwise effects from an omnibus test or assume that every interaction is a crossover.

S8.5. Disagreements and conflicting source information

C.T. completed primary extraction, and T.G. checked the extracted content against the full texts. They compared their assessments and resolved discrepancies through discussion to reach consensus. Source passages and locators supported the retained values.

A disagreement between the reviewers is distinct from a contradiction within a source. When Results text, tables, figures, or supplements disagreed, the extraction preserved the relevant source statements. A correction required an identifiable reporting, label, or transcription issue. An unresolved source contradiction retained a conflict status. A Discussion statement did not overwrite a contradictory Results statement solely because it offered a clearer interpretation.

The canonical data include 44 rows with `analysis_eligibility=descriptive_only_source_conflict`. They remain in the result inventory and descriptive source discussion. The family-pattern calculation excludes them before collecting direction labels. Quantitative feasibility assessment also excludes them. Their presence does not remove the other rows from the same study family. A conflict row may retain its source-level direction code, so the conflict status must be checked separately from `effect_sign`.

S8.6. Family aggregation

The analysis script first groups rows by `study_family_id`. It removes rows marked `descriptive_only_source_conflict` from the direction-pattern calculation. It then collects the set of remaining direction labels. Repeated occurrences of a label do not give that label additional weight.

For this calculation, the internal set named `detected` contains `positive`, `negative`, and `mixed`. The family labels containing the word `detected` describe membership in this set. They do not mean that every

contributing row passed an independently applied significance test. The script uses the following conditions in the order shown.

First satisfied condition	Family label	Families
At least one positive, negative, or mixed code and at least one no-difference code	`detected_and_null_results`	210
No preceding condition; at least one mixed code, or both positive and negative codes	`mixed_detected_directions`	37
No preceding condition; positive is the only member of the internal detected set	`positive_only`	32
No preceding condition; negative is the only member of the internal detected set	`negative_only`	1
No preceding condition; at least one no-difference code	`null_only`	13
No preceding condition	`unclear_or_descriptive_only`	1

An `unclear` row does not change a classification supported by other labels. Thus, `positive_only` can coexist with unclear rows. It means that no retained negative, mixed, or no-difference code is present. Likewise, `null_only` can coexist with unclear rows. A family with positive, negative, and no-difference rows falls in `detected_and_null_results` because the first condition takes precedence.

These categories summarize the composition of a family's charted results. They do not average effects, select the most significant outcome, weight sample sizes, or determine an intervention's effectiveness. Their interpretation also depends on the number and type of outcomes reported by each family. The row-level inventory contains 1,665 positive, 590 negative, 1,961 no-difference, 492 mixed, and 215 unclear codes, including the 44 conflict rows. The family calculation applies the exclusions described above.

S8.7. Traceable examples

The companion file `S8_traceable_examples.csv` contains the exact Table S3 values for the following examples. Data-row numbers exclude the header and are supplied for convenience; the seven-field keys identify the records.

Positive direction despite a negative test statistic. In Park and Choi's facial-color study (R008123), red faces had higher anger-recognition accuracy than yellow faces. The charted code is positive, while the reported paired-test value is t = -2.570, p = .014. The direction follows the reported comparison, not the sign of the test statistic. Source: Section 3.1, accuracy follow-up paired t tests. S3 data row 481.

Key: `SF0077|R008123|E1|S1|C_red_vs_yellow_anger|O_accuracy_anger|T_judgment_task`.

Task-oriented positive code. Kang et al. (2022; R008347) reported faster responses to sad faces after blue than red primes. The positive code records facilitation. Source: Experiment 1 Results; Bonferroni-corrected paired comparison, t = 6.214, p < .001. S3 data row 487.

Key: `SF0080|R008347|E1|S1|C_blue_sad_vs_red|O_RT_blue_sad|T_color_priming`.

Numerical negative code for shorter latency. Domínguez-Borràs et al. (2020; CC20260804_03698) reported 1,526 ms with a red-tinted cue and 2,344 ms with a neutral identity cue. This reduction in latency is coded negative. Source: Table 4, PDF pages 43 to 44; control-group Results, PDF page 19; t = -7.46, p < .0001. S3 data row 4045.

Key: `SF0211|CC20260804_03698|E02_visual_search|S03_controls_VS|C008|O008|TW_overall_RT`.

Mixed conditional direction. Minami et al. (2018; R006593) reported an interaction between color location and hue in the Color Effect Index. Red and blue shifted judgments toward different expressions. The row is coded mixed. Source: Results, Color Effect Index, Figure 2; F(1,11) = 8.55, p = .014. S3 data row 4.

Key: `SF0004|R006593|1|1|Locus_x_Hue|CEI_interaction|NA`.

Significant test with unclear direction. Ciccone (2018; R000624) reported an omnibus color effect on total theta activity, F(4,16) = 5.63, p = .005. The direction remains unclear in the charted row. Source: page 138, Table 26 and Figure 50. S3 data row 99.

Key: `SF0023|R000624|E3|S3|C4|O5|T1`.

Narrative direction with missing statistics. Wang et al. (2025; R015112) described lower frustration under a yellow interface background. The row is coded negative. Exact statistics are not reported. Source: page 378, Results. S3 data row 640.

Key: `SF0117|R015112|Exp1|S1|C05|O_frustration|TW_all`.

No detected association under the source threshold. Kübel et al. (2020; CC20260811_05757) reported an association between the red-condition passage-of-time rating and volitional control, r = -.367, p = .030. The .001 threshold for the correlation set gives the stored no-difference classification. Source: PDF pages 7 to 9, Table 2. S3 data row 3657.

Key: `SF0279|CC20260811_05757|E01|S01|C002|O002|TW01`.

Different threshold within the same report. The red versus green duration comparison in Kübel et al. used a one-sided paired test with alpha = .0125. Estimated duration was greater under red, with p = .011. The row is coded positive. Source: PDF pages 9 to 10, Table 3. S3 data row 3688.

Key: `SF0279|CC20260811_05757|E01|S01|C033|O033|TW01`.

Conflict status takes precedence in aggregation. The depression and red-color Stroop-interference row for women in Ioan et al. (2007; CC20260804_05368) retains a no-difference code and a verified-conflict status. Its analysis eligibility restricts it to description. The family-pattern script therefore excludes it regardless of the direction label. Source: Results Section 3.5, PDF page 5, Figure 4B. S3 data row 4699.

Key: `SF0225|CC20260804_05368|E01_color_word_stroop|S01|C017|O017|TW_color_naming_response`.

S8.8. Reproduction files

`S8_family_pattern_reproduction.csv` lists the non-conflict label counts and resulting category for every family. `reproduce_S8_family_patterns.py` reproduces the categories from Table S3 without changing its data. The script uses the Python standard library. The command below writes a family table and a summary with the input hash. The rule order corresponds to the family-pattern calculation in the combined analysis script, which the pre-submission update reused.

python reproduce_S8_family_patterns.py Table_S3.csv --output-dir S8_reproduced

Replace `Table_S3.csv` with the name or path of the accompanying canonical result table.
